# Analysis of Cell Wall-Related Genes in Organs of *Medicago sativa* L. under Different Abiotic Stresses

**DOI:** 10.3390/ijms160716104

**Published:** 2015-07-16

**Authors:** Marc Behr, Sylvain Legay, Jean-Francois Hausman, Gea Guerriero

**Affiliations:** 1 Environmental Research and Innovation (ERIN), Luxembourg Institute of Science and Technology (LIST), 5, Avenue des Hauts-Fourneaux, L-4362 Esch/Alzette, Luxembourg; E-Mails: marc.behr@list.lu (M.B.); sylvain.legay@list.lu (S.L.); 2 Groupe de Recherche en Physiologie végétale, Earth and Life Institute—Agronomy, Université catholique de Louvain, 5 (bte 7.07.13) Place Croix du Sud, B-1348 Louvain-la-Neuve, Belgium

**Keywords:** abiotic stresses, gene expression, peroxidases, dirigent proteins, cellulose synthases, cell wall

## Abstract

Abiotic constraints are a source of concern in agriculture, because they can have a strong impact on plant growth and development, thereby affecting crop yield. The response of plants to abiotic constraints varies depending on the type of stress, on the species and on the organs. Although many studies have addressed different aspects of the plant response to abiotic stresses, only a handful has focused on the role of the cell wall. A targeted approach has been used here to study the expression of cell wall-related genes in different organs of alfalfa plants subjected for four days to three different abiotic stress treatments, namely salt, cold and heat stress. Genes involved in different steps of cell wall formation (cellulose biosynthesis, monolignol biosynthesis and polymerization) have been analyzed in different organs of *Medicago sativa* L. Prior to this analysis, an *in silico* classification of dirigent/dirigent-like proteins and class III peroxidases has been performed in *Medicago truncatula* and *M. sativa*. The final goal of this study is to infer and compare the expression patterns of cell wall-related genes in response to different abiotic stressors in the organs of an important legume crop.

## 1. Introduction

Exogenous constraints of biotic and abiotic nature are major threats to crop productivity. They can impact plant growth and development at different levels, with strong biomass penalties, consequent losses in agriculture and biodiversity [1]. In nature, plants are exposed to different exogenous stresses, often in combination. Studies have been devoted to understanding the effects of multiple stresses on plants. What emerged from these analyses is that different, as well as common response mechanisms, are present among the various constraints applied. Moreover the response to multiple stresses is not predictable by considering the single treatments [2], since the effects are not always additive [3,4]. Therefore the study of plant response to environmental stresses can help understand better the mechanisms underlying adaptation [5] and devise strategies improving plant tolerance to exogenous stresses, for example by targeting specific metabolic pathways [6] or by advanced breeding programs [7]. Much of the knowledge so far acquired on the response of plants to abiotic stress has been generated via integrative studies using different *–omics*, namely genomics, transcriptomics, proteomics, metabolomics [2,5,8,9]. These studies have reported significant changes in the expression/abundance of genes/proteins involved in cell wall metabolism and have shown how mutations in cell wall-related genes can alter the response of plants to abiotic stresses [10,11,12,13]. For example, *Arabidopsis thaliana* cellulose synthase 3 (*CesA3*) mutants show constitutive response to stress because they have an enhanced production of jasmonate and ethylene [10], while mutations in secondary cell wall *CesA*s (e.g., *CesA4*, *CesA7*, *CesA8*) activate constitutive ABA signaling and confer enhanced resistance to drought and osmotic stress [11,12]. More recently an expansin-like gene (*EXLA2*) from *A. thaliana* was shown to be involved in the response to necrotrophic fungi, as well as abiotic stresses. A mutation in this gene causes ABA-mediated hypersensitivity to salt and cold stress [13].

It is now widely demonstrated that the plant cell wall maintains cell wall integrity (CWI) in response to exogenous stresses by triggering modifications in the cell wall and the metabolism [14,15]. Although the detailed mechanisms and components have not yet been fully unveiled, plant CWI maintenance shares similarities with the yeast system [14,15]. The sensing of the cell wall status is carried out by receptor-like-kinases (RLKs) (reviewed extensively in [15,16]), comprising, among other members, THESEUS1 [17], FERONIA [18], HERKULES1/2 [19], which unleash a signaling cascade involving reactive oxygen species (ROS), as well as plant growth regulators (namely abscisic acid, salicylic acid, jasmonic acid). Therefore besides enveloping the living protoplasts, cell walls are important structures that actively take part in the cross-talk with the environment, via the *continuum* cell wall-plasma membrane-cell interior. Upon abiotic stress this *continuum* can be affected, for example by displacing the plasma membrane with respect to the cell wall, following alterations of turgor pressure on a weakened cell wall [15].

Upon abiotic stress, an organ-specific cell wall remodeling takes place [4], which ultimately leads to changes in structure and composition. An emblematic example is represented by the shoot and the roots under abiotic stress: while the former has to limit growth (as for example under drought stress), the latter relies on cell walls ensuring continued growth in order to explore the soil for water [4]. Understanding more about the cell wall-related processes taking place in plant organs subjected to abiotic stress can therefore not only provide a clearer picture of this complex phenomenon, but also disclose important information that can be used to develop engineering strategies focused on the cell wall and aimed at improving plant tolerance to exogenous stresses.

We have here analyzed the expression of genes involved in cell wall biosynthesis, *i.e.*, cellulose synthases (*CesA*s), as well as genes involved in lignin deposition, namely phenylalanine ammonia-lyase (PAL), cinnamyl alcohol dehydrogenase (CAD), three dirigent-like proteins and three class III peroxidases. This analysis is preceded by a bioinformatic survey of the putative dirigent/dirigent-like proteins and class III peroxidases in *Medicago sativa* and the closely related model plant *Medicago truncatula*, since studies have proven the suitability of using barrel medic transcripts to address molecular studies in alfalfa [20]. We recently published data concerning *CesA*, PAL, CAD, sucrose synthase (SuSy) and a cellulose synthase-like gene expression in stems of alfalfa subjected to cold, heat and salt stress treatment [21]. The purpose of this study is to complement these data by extending the analysis to genes involved in monolignol biosynthesis and polymerization, and provide further insights into the organ-specific expression dynamics of cell wall-related genes upon abiotic stresses in an economically relevant legume.

## 2. Results and Discussion

### 2.1. Dirigent and Dirigent-Like Protein Sequences in Medicago truncatula

Dirigent proteins are proteins lacking catalytic activity and implicated in plant secondary metabolism. They were shown to be involved in lignan production [22], as well as lignification [23,24] and (a)biotic stress response [25]. Dirigent proteins confer stereo-selectivity to the oxidative coupling of coniferyl alcohol, thereby favouring the production of either (+)- or (−)-pinoresinol [26]. Mining of the *M. truncatula* genome for dirigent proteins led to the identification of 45 genes. BLAST analysis of the identified *M. truncatula* dirigent proteins against the alfalfa EST database at NCBI led to the identification of three ESTs, namely GenBank accessions CO514440.1, CO515037.1 and EX525320.1 from glandular trichome libraries [27]. The first two ESTs are orthologs of Medtr8g073770.1 and Medtr8g073850.1 respectively, while the third EST is the ortholog of Medtr1g054525.1.

**Figure 1:**
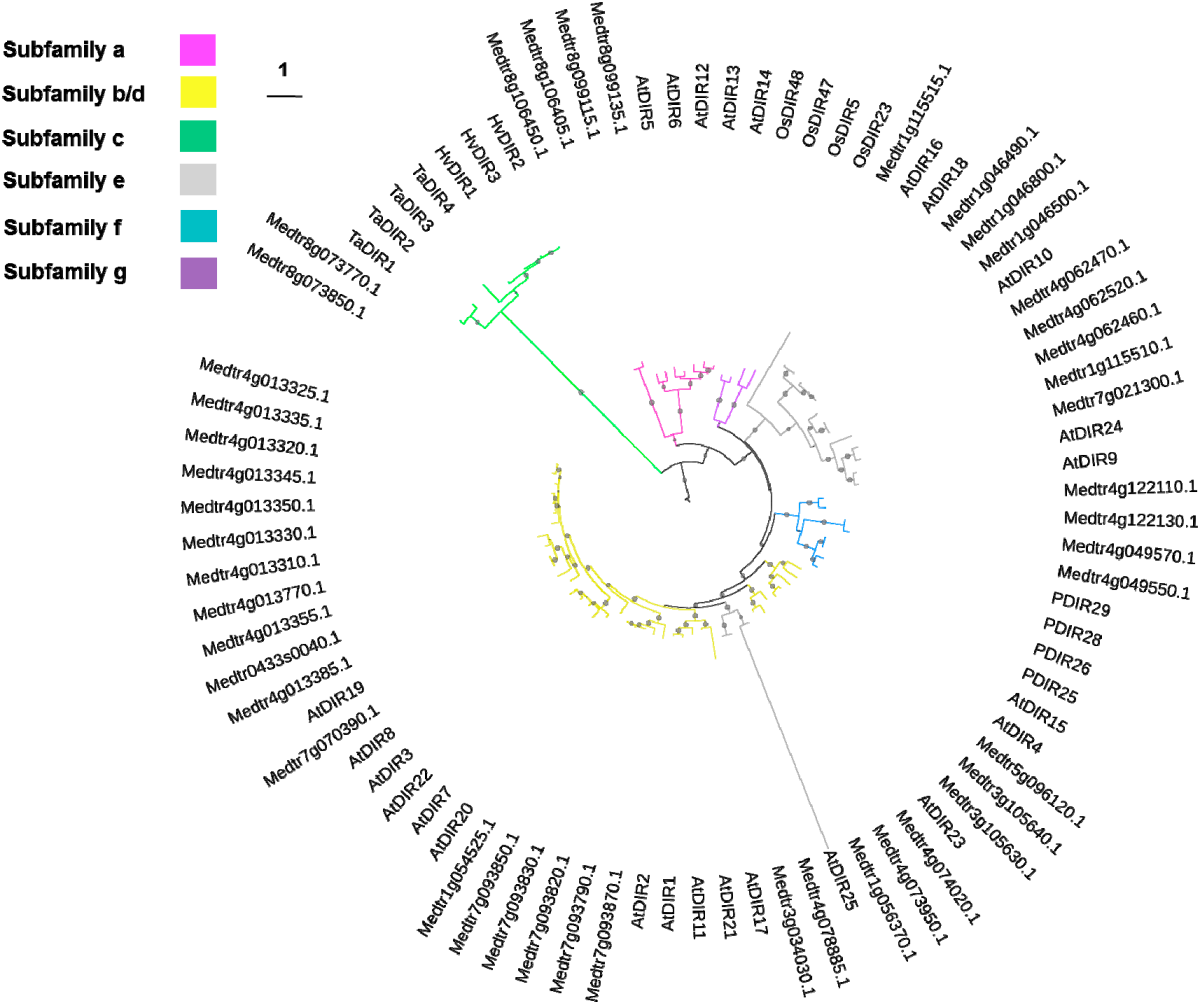
Phylogenetic relationships of dirigent and dirigent-like protein from *Medicago truncatula*, *Arabidopsis thaliana*, *Picea sitchensis*, *Oryza sativa*, *Hordeum vulgare* and *Triticum aestivum*. The scale bar indicates an evolutionary distance of 1 amino acid substitution per position. The different subfamilies are indicated with different branch colours (according to [28]). Only bootstrap values >0.90 are indicated (small gray circle) for visual clarity. At: *A. thaliana*, P: *P. sitchensis*, Ta: *T. aestivum*, Hv: *H. vulgare*, Os: *O. sativa*. The accession numbers used to build the tree are indicated in the Materials and Methods section.

The number of putative dirigent/dirigent-like proteins in *M. truncatula* is quite high and no phylogenetic analysis has been carried out so far on this important legume. To provide a phylogenetic classification of the *M. truncatula* dirigent/dirigent-like proteins, a comparison was carried out with proteins from other species. The phylogenetic analysis of *M. truncatula*, *A. thaliana*, *Picea sitchensis*, *Oryza sativa*, *Hordeum vulgare* and *Triticum aestivum* dirigent proteins showed that the identified barrel medic sequences branch into subfamilies a, b/d, e and f (Figure 1). However two genes, Medtr8g073770.1 and Medtr8g073850.1, could not be assigned to any specific subfamily (Figure 1). Subfamily a contains members that are being studied for their biochemical role [28], and four barrel medic sequences, *i.e.*, Medtr8g099115.1, Medtr8g099135.1, Medtr8g106450.1 and Medtr8g106405.1 belong to this cluster. Members of subfamily a are referred to as dirigent proteins, while the others are named dirigent-like proteins [28,29].

The clade with the highest number of *M. truncatula* sequences is clade b/d with 23 sequences, while no alfalfa sequences branch within clade g (Figure 1), which is represented by rice dirigent-like proteins.

The majority of the genes were predicted to code for secreted proteins (Supplementary Table S1) with TargetP ([30]; Available online: http://www.cbs.dtu.dk/services/TargetP/). Moreover all the sequences, except Medtr7g021300.1 and Medtr4g074020.1, were predicted to contain *N*-glycosylation sites (Supplementary Table S1) with the NetNGlyc 1.0 server ([31]; Available online: http://www.cbs.dtu.dk/services/NetNGlyc/). The genes Medtr1g046800.1, Medtr4g062520.1 and Medtr4g062470.1 were predicted to contain a putative *N*-glycosylation site followed by a proline, which is very unlikely to be modified (Supplementary Table S1).

Alignment of the *M. truncatula* sequences revealed the presence of the five conserved motifs described by Ralph and colleagues [29] (Supplementary Figure S1).

All of the identified genes code for mature proteins whose predicted 3D structures have as top-scoring hit (confidence > 90%) the disease resistance response protein 206 from *Pisum sativum* (DRR206, PDB code 4REV). All of the genes, except Medtr4g074020.1 which lacks the 3rd, 4th and 5th conserved domain described in dirigent proteins (Supplementary Figure S1), belong indeed to the *all* β*-protein* fold class and are characterized by β-strands connected by coils [32]. In Supplementary Figure S2 a representative model is shown, which corresponds to the mature dirigent-like protein Medtr1g054525.1 (aa 27–192), one of the targets analysed via RT-qPCR in this study.

**Figure 2:**
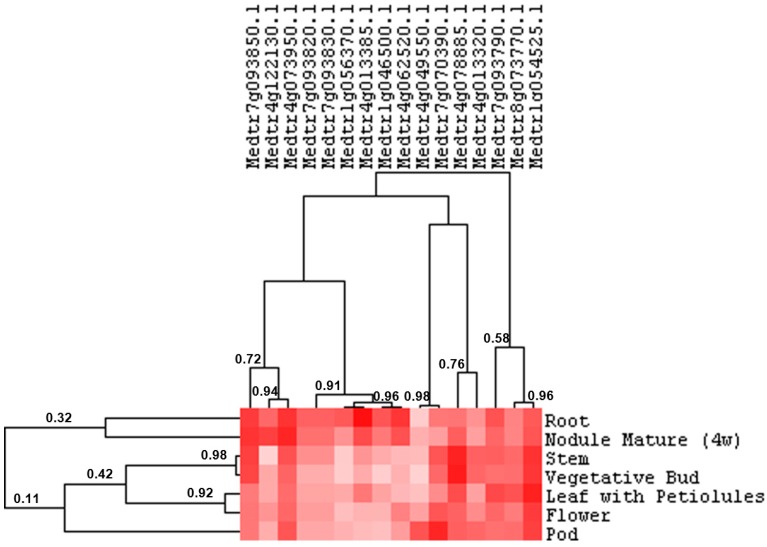
Organ-specific expression profiles of 16 *M. truncatula* genes coding for dirigent and dirigent-like proteins (retrieved from the *M. truncatula* eFP browser [33]). The heat map is drawn on the values retrieved from the eFP browser (the values ± standard deviations are shown in Supplementary Table S2). Pixel colour intensity is proportional to the actual expression values. The length of the branches, represented by the numbers, refers to the Pearson correlation coefficients among tissues and genes. Nodule mature (4w) refers to nodules samples from plants aged four weeks. Petiolules refer to the stalk present at the base of each of the three leaflets composing the trifoliate leaf of *M. truncatula*.

To shed light on the potential role of the identified dirigent and dirigent-like proteins, their expression profiles in different *M. truncatula* organs were retrieved from the *Medicago* eFP browser (Available online: http://bar.utoronto.ca/efpmedicago/cgi-bin/efpWeb.cgi) [33]. Organ-specific expression profiles could be retrieved for 16 genes. The hierarchical clustering shows two main groups, one characterized by genes expressed preferentially in roots/nodules and one group represented by genes expressed more homogeneously in the different organs (Figure 2).

To get information concerning the differential expression in alfalfa organs, we designed primers on those genes showing expression in roots, stems and leaves. These targets are Medtr7g093850.1 and Medtr4g073950.1 within cluster one and Medtr4g078885.1, Medtr8g073770.1 and Medtr1g054525.1 within cluster two. However only the primers designed on Medtr7g093850.1, Medtr1g054525.1 and Medtr4g078885.1 gave one specific peak after melt curve analysis (Supplementary Figure S3). The expression of these genes was analysed via RT-qPCR (see Section 2.3, Section 2.4 and Section 2.5).

### 2.2. Class III Peroxidase Protein Sequences in M. truncatula and M. sativa

**Figure 3:**
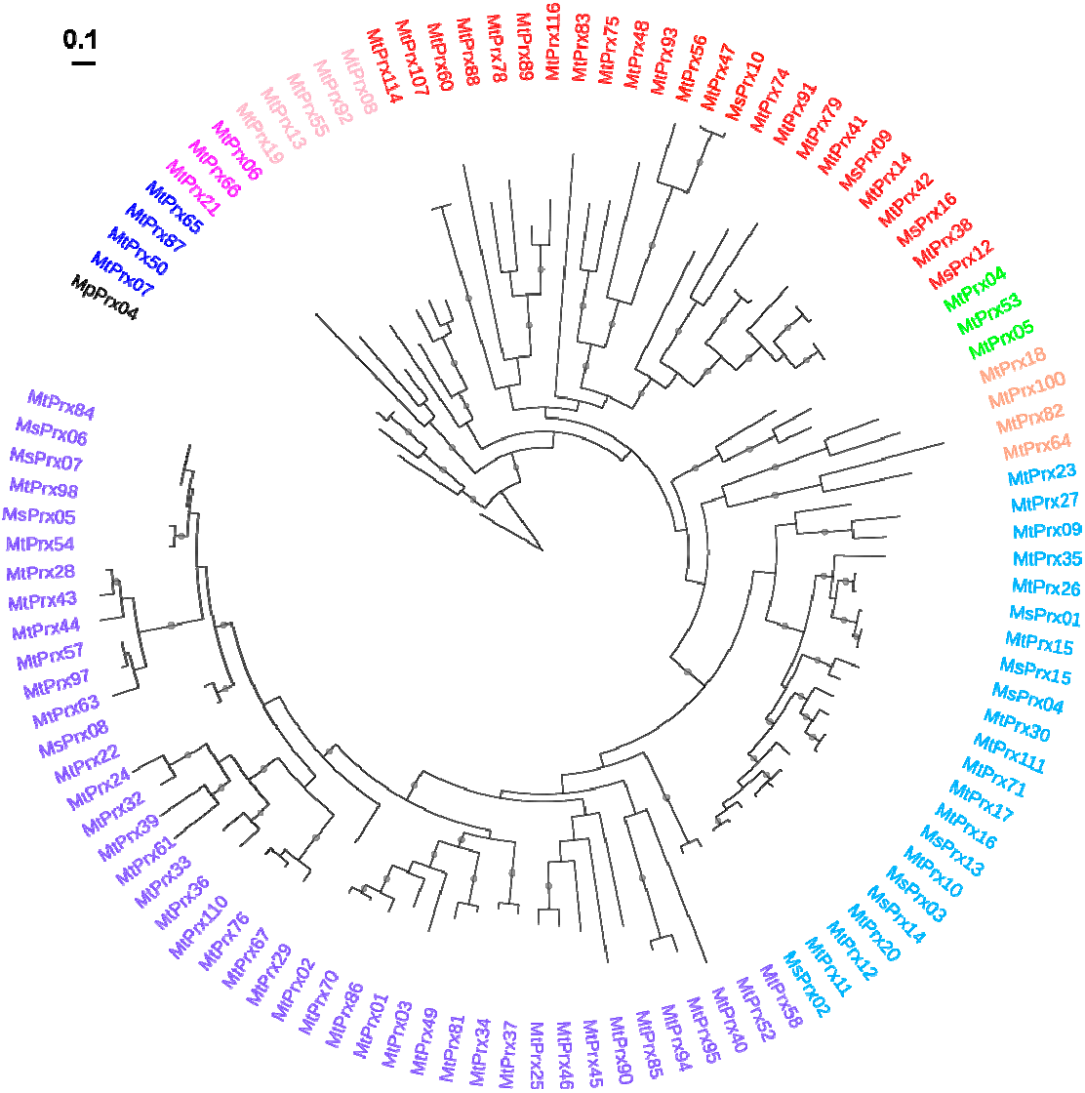
Phylogenetic relationships of class III peroxidases from *M. truncatula* and *M. sativa*. The peroxidases belonging to the same class are indicated with the same name colour (class I: blue, class II: fuchsia, class III: pink, class IV: red, class V: green, class VI: orange, class VII: turquoise, class VIII: violet). The peroxidase from *Marcanthia polymorpha* (MpPrx04, GenBank Accession BJ842248) was used to root the tree. The scale bar indicates an evolutionary distance of 0.1 amino acid substitutions per position. Only bootstrap values >0.90 are indicated (small gray circle) for visual clarity. *M. truncatula* gene accessions are indicated in Supplementary Table S3. *M. sativa* gene accessions are indicated in the Materials and Methods section.

Class III peroxidases are plant-specific haem-containing enzymes belonging to a large multigenic family and involved in several processes, namely (a)biotic stress response, suberization, lignification and auxin catabolism [34,35]. The redundancy of class III peroxidases in plants makes it challenging to study their role from a functional point of view, since often no clear phenotypes are visible when the expression of a specific gene is manipulated [36]. In *A. thaliana* 73 class III peroxidases have been reported [37] and this number goes up to 138 in rice [38] and 119 in maize [39]. Search in the PeroxiBase database [40] led to the identification of 15 and 102 class III peroxidases for *M. sativa* and *M. truncatula* respectively. Of the 102 genes, one is incomplete (MtPrx116) and nine are pseudogenes (namely Medtr2g008730.1, Medtr5g058120.1, Medtr5g074770.1, Medtr2g029880.1, Medtr6g048160.1, Medtr5g074700.1, Medtr4g114220.1, Medtr7g072490.1, Medtr1g098320.1). Phylogenetic analysis of the putative peroxidases (not including the pseudogenes) shows the presence of eight main groups (Figure 3), in particular groups I to III, V, VII and VIII are well supported by bootstrap values (>0.85).

**Figure 4:**
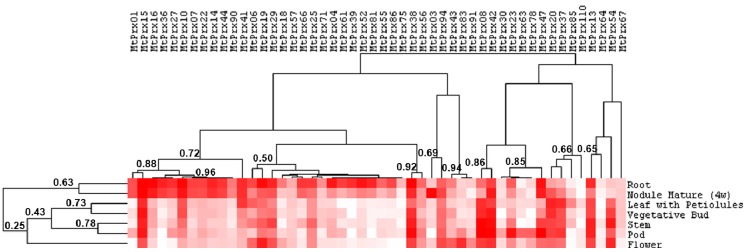
Organ-specific expression profiles of *M. truncatula* class III peroxidases (retrieved from the *M. truncatula* eFP database [33]). The values ± standard deviation retrieved from the eFP browser are shown in Supplementary Table S4. Pixel colour intensity is proportional to the actual expression values. The length of the branches, represented by the numbers, refers to the Pearson correlation coefficient among tissues and genes. Nodule mature (4w) refers to nodules samples from plants aged 4 weeks. Petiolules refer to the stalk present at the base of each of the three leaflets composing the trifoliate leaf of *M. truncatula*.

The identified genes code for proteins showing the conserved motifs spanning the two conserved His and eight conserved Cys described in [38], with the exception of MtPrx32, MtPrx60, MtPrx61 and MtPrx91, which lack the first conserved His in the motif F/YHDC and show instead a Gln (MtPrx32 and MtPrx61) or a Ser (MtPrx60 and MtPrx91) (Supplementary Figure S4). Expression profiles were retrieved for 50 genes from the *M. truncatula* eFP database (Figure 4). The hierarchical clustering analysis of *M. truncatula* class III peroxidases identifies two main groups: the first is characterized by genes showing a preferential expression in roots and nodules, the second by genes expressed also in aerial organs (Figure 4). Within the second cluster some of the genes show a tissue-specific expression, namely MtPrx83 in the flower, MtPrx78 and MtPrx63 in the pod (Figure 4).

Since some of the identified genes show a high expression in the stem (e.g., MtPrx08, MtPrx13, MtPrx42, MtPrx47, MtPrx54), an organ characterized by tissues undergoing secondary growth, an analysis of the promoter regions (2000 bp upstream the start codon) was carried out to look for the occurrence of specific motifs linked to the deposition of secondary cell walls. As can be seen from Supplementary Table S3, some of the identified class III peroxidases (even those that are not strictly stem-specific) show the presence of the XYLAT element (ACAAAGAA), which is a sequence found in the promoters of genes belonging to the so-called “*core xylem gene set*” [41]. This might indicate a role of these genes in the genetic program controlling secondary cell wall deposition in *M. truncatula*.

On the basis of the expression pattern in *M. truncatula*, we decided to target peroxidases showing expression in roots, leaves and stems for the RT-qPCR analysis on alfalfa plants. This approach enables comparison of the expression patterns in the different organs under the various abiotic stresses applied, as previously discussed for the dirigent/dirigent-like proteins. The peroxidases retained for the analysis are MtPrx13, MtPrx38 (whose ortholog in alfalfa is MsPrx12) and MtPrx42 (whose ortholog in alfalfa is MsPrx16), because the primers designed on these genes gave one specific peak after melt curve analysis (Supplementary Figure S5). MsPrx16 gave however two melt curve peaks in the leaves (Supplementary Figure S5), therefore this gene was not retained for analysis in these organs.

### 2.3. Cell Wall-Related Gene Expression in Alfalfa Roots under Abiotic Stress

**Figure 5:**
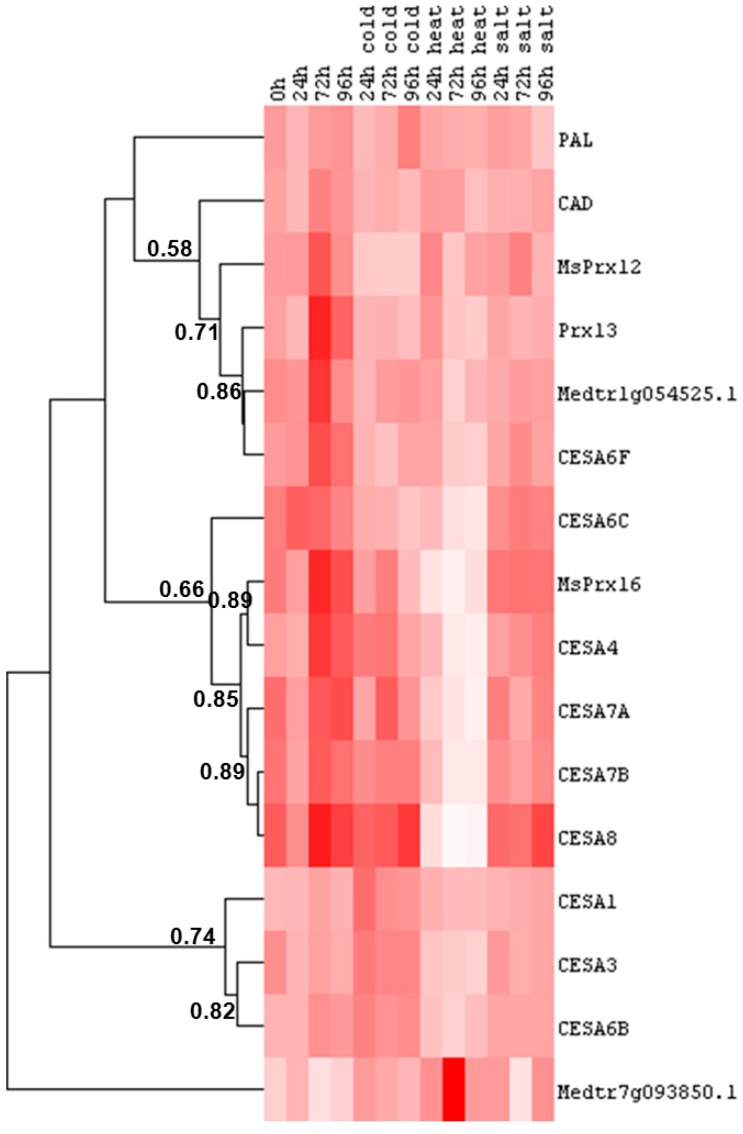
Heat map representation of the data reported in Table S5 and Table S6 showing the hierarchical clustering of cell wall-related genes in response to abiotic stresses at different time points in alfalfa roots. The numbers indicate the Pearson gene correlation coefficient. Pixel colour intensity is proportional to the actual expression values.

The hierarchical clustering analysis in alfalfa roots of the cell wall-related genes here studied shows three main groups: the first is characterized by genes displaying decreased expression under cold and heat stress, the second group is represented by genes showing a marked down-regulation under heat stress and the third one includes genes that do not show significant differences in expression under the different stress conditions studied (Figure 5). Genes involved in monolignol biosynthesis and polymerization, namely PAL and CAD (with a lower coefficient of correlation), Prx13, MsPrx12, belong to the first group, together with the dirigent-like protein Medtr1g054525.1 and a *CesA* putatively involved in primary cell wall biosynthesis (*CesA6F*). The values for CAD, PAL, are not statistically significant, while the decrease observed at 72 h of cold and heat stress for the dirigent-like protein Medtr1g054525.1 is significant (Supplementary Table S6). Likewise the decrease in *CesA6F* expression at 72 and 96 h of heat stress is statistically significant (Supplementary Table S5). Prx13 shows a significant decrease in expression at 72 and 96 h of cold stress; MsPrx12 is significantly down-regulated at 72 h of cold stress (Supplementary Table S6).

Chilling stress was previously reported to affect the cell walls of cucumber root tips which appeared thinner and distorted and became disintegrated in several places at more advanced stages of injury [42]. However, in plants subjected to cold stress, an increased thickness and rigidity of the cell walls has also been observed [43]. Cold induces the formation of ice which, by propagation, exerts mechanical stress at the cell wall level [43]. One strategy to limit the propagation of ice is to rigidify the cell wall and decrease the size of the cell wall pores [44]. A direct consequence of increased cell wall rigidity is organ growth reduction: indeed, reduced growth of roots and aerial organs has been observed in plants subjected to chill stress ([43] and references therein). In alfalfa roots however genes involved in cell wall strengthening (*i.e.*, those involved in lignin monomer synthesis and polymerization) showed decreased expression under cold stress (Figure 5). This result can be interpreted as a general strategy of alfalfa root cell walls to maintain flexibility under temperature stress. This conclusion is supported by the marked decrease in expression observed under heat stress for the cluster of genes involved in secondary cell wall deposition (Figure 5). Secondary *CesA*s, as well as a class III peroxidase (MsPrx16) and a primary *CesA* (*CesA6C*) showed indeed a statistically significant decrease in expression (Table S5 and Table S6). In *Nicotiana tabacum* an enhanced flexibility of the cell wall increased heat stress tolerance: indeed the heterologous expression in tobacco of an α-expansin from the perennial grass species *Poa pratensis* reduced the structural damages, lipid peroxidation, electrolyte leakage and hydrogen peroxide production at 42 °C, compared to wild-type plants [45]. However care should be taken in drawing general conclusions, as the cell wall response to heat stress can be different among plant species and organs. Expansin genes were indeed down-regulated in *Populus simonii* and rapeseed seedlings under heat stress [5,46,47].

The primary *CesA*s *CesA1*, *CesA3*, *CesA6B* belong to the third group of genes and do not show significant changes in expression under the various stresses studied (apart from a mild, but statistically significant decrease in *CesA6B* expression upon heat stress at 72 h and a slight significant increase in *CesA1* expression at 24 h of cold stress; Supplementary Table S5) (Figure 5). The dirigent-like protein Medtr7g093850.1 branches alone and is characterized by a statistically significant up-regulation at 72 h of heat stress (Figure 5 and Supplementary Table S6).

### 2.4. Cell Wall-Related Gene Expression in Alfalfa Leaves under Abiotic Stress

**Figure 6:**
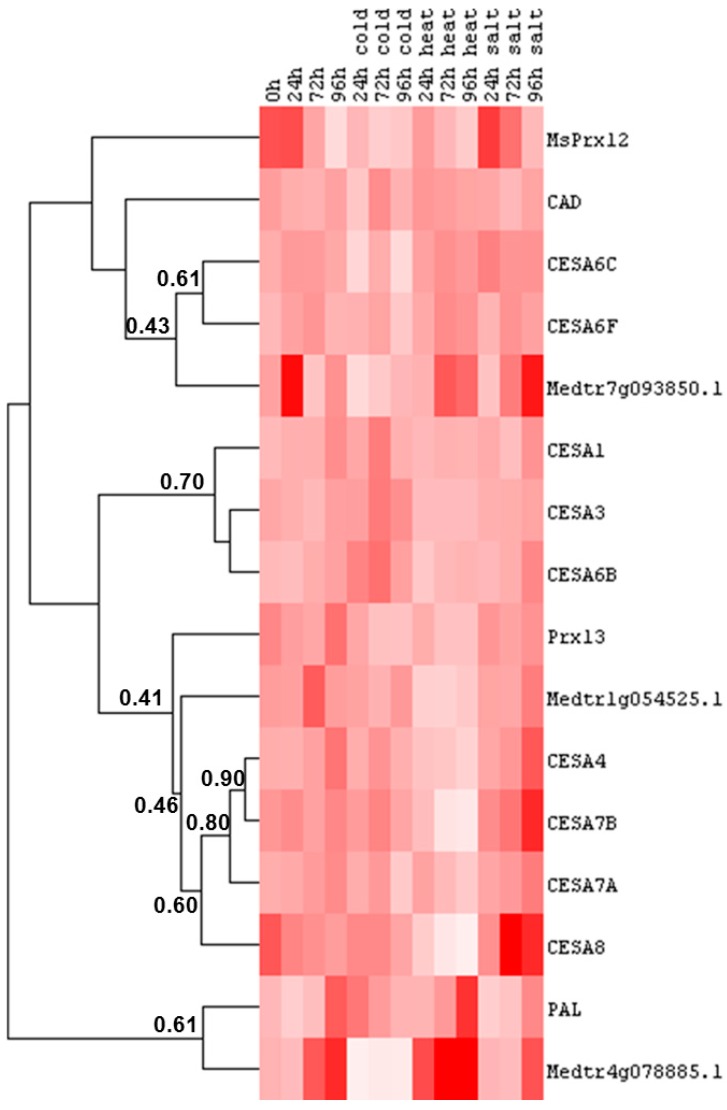
Heat map representation of the data reported in Supplementary Tables S7 and S8 showing the hierarchical clustering of cell wall-related genes in response to abiotic stresses at different time points in alfalfa leaves. The numbers indicate the Pearson gene correlation coefficient. Pixel colour intensity is proportional to the actual expression values.

In alfalfa leaves subjected to abiotic constraints it is possible to recognize three main groups of genes and a fourth cluster composed of PAL and a gene coding for a dirigent-like protein (Figure 6). The correlation among the genes within a cluster is however lower than the one observed in roots, with the exception of *CesA4* and *CesA7B* (Figure 6). The first group is characterized by genes whose expression decreases in response to cold stress; the second cluster shows an increase in expression at 72 h of cold stress; the third group is characterized by genes with lower expression levels under heat stress and the fourth cluster groups two genes showing the same tendency to increase under heat stress and later stages of salt stress (Figure 6).

The first cluster of genes is represented by *CesA6C*, *CesA6F* and the dirigent-like protein Medtr7g093850.1. To this first group belong also CAD and MsPrx12, but with a lower correlation (Figure 6). The decrease under cold stress is statistically significant only for *CesA6C* at 24 and 96 h and for MsPrx12 at 24 h (Supplementary Tables S7 and S8).

The primary *CesA*s *CesA1*, *CesA3* and *CesA6B* belong to the second group (Figure 6). The peak in expression after 72 h of cold stress is significant for the three genes (Supplementary Table S7).

The third group of genes comprises the secondary cell wall *CesA*s *CesA4*, *CesA7A*, *CesA7B*, *CesA8*. To this cluster, but with a lower correlation, belong also the peroxidase Prx13 and the gene coding for the dirigent-like protein Medtr1g054525.1 (Figure 6). Within this cluster, *CesA7B* and *CesA8* show a statistically significant decrease in expression at 72 and 96 h of heat stress (Figure 6, Supplementary Tables S7 and S8). *CesA4* decreases significantly at 96 h of heat stress and Medtr1g054525.1 shows a significant down-regulation at earlier stages of heat stress (*i.e.*, 24 and 72 h) (Supplementary Tables S7 and S8). Although not statistically significant, the secondary *CesA*s *CesA7B* and *CesA8* show a tendency to up-regulation under salt stress. These results are interesting if one considers the data recently published on salt-stressed maize leaves [48]. This study indeed revealed that salinity stiffens the cell walls of leaf epidermal cells in salt-sensitive maize thereby impairing organ growth. However in our experimental set-up the monolignol biosynthetic genes do not show a statistically-significant increase in expression (Supplementary Table S8). It remains to be elucidated whether a cell wall stiffening mechanism occurs in alfalfa leaves under salt stress and whether it involves genes implicated in the biosynthesis of other secondary cell wall components, as for instance xylan.

PAL and the gene encoding the dirigent-like protein Medtr4g078885.1 cluster on a separate branch (Figure 6). The decrease in expression observed for Medtr4g078885.1 under cold stress is statistically significant for all the time-points considered (Supplementary Table S8). This trend is opposed to its behaviour under heat stress, which is similar to the one observed for PAL. Although statistically not significant, this tendency to increase for both genes suggests the occurrence of alterations in secondary metabolism in stressed alfalfa leaves. PAL transcripts were shown to increase in response to heat in mandarin and banana fruits and to confer enhanced chill tolerance [49,50]. It is indeed documented that thermal stress induces the accumulation of phenolics (and inhibits their oxidation), which are involved in acclimation to heat stress [51].

### 2.5. Cell Wall-Related Gene Expression in Alfalfa Stems under Abiotic Stress

**Figure 7:**
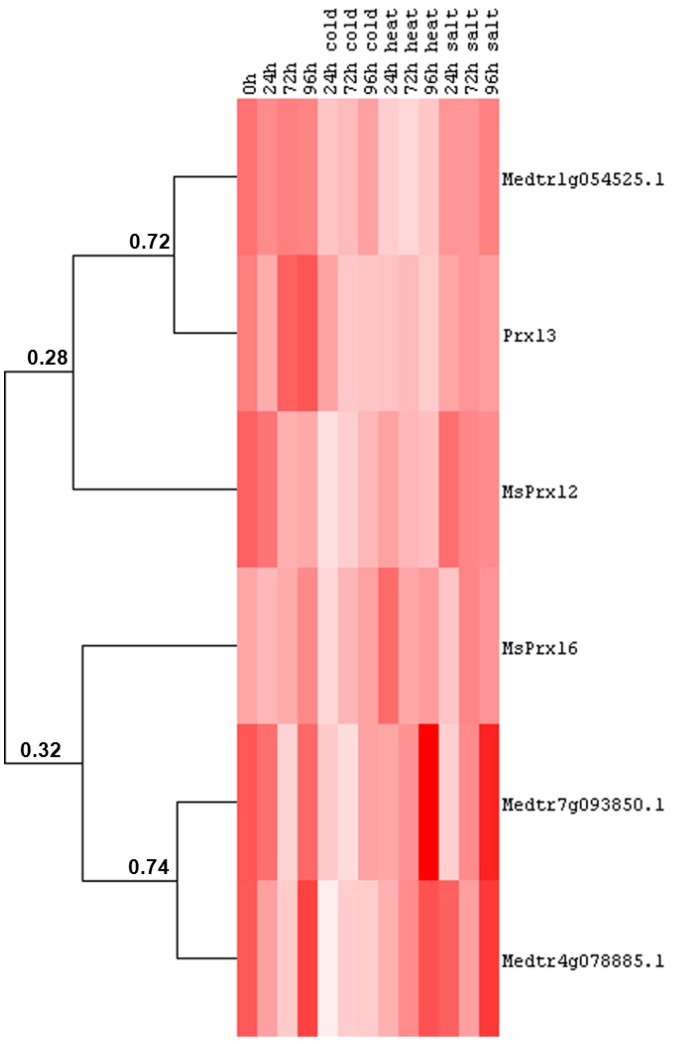
Heat map representation of the data reported in Supplementary Table S9 showing the hierarchical clustering of cell wall-related genes in response to abiotic stresses at different time points in alfalfa stems. The numbers indicate the Pearson gene correlation coefficient. Pixel colour intensity is proportional to the actual expression values.

In stems of alfalfa subjected to abiotic stresses we previously identified the expression of *CesA*s, a cellulose synthase-like gene, sucrose synthase, PAL and CAD [21] and showed the presence of two main patterns, *i.e.*, a salt/heat-induced and a cold/heat-repressed group of genes [21]. We here provide data concerning the expression dynamics of other cell wall-related genes in alfalfa stems under different abiotic stresses. As can be seen from Figure 7, the gene coding for the dirigent-like protein Medtr1g054525.1, Prx13 and MsPrx12 belong to the cluster of genes showing decreased expression under cold/heat stress treatment. The decrease observed is not significant for MsPrx12 under heat stress (Supplementary Table S9). The two genes encoding the dirigent-like proteins Medtr7g093850.1 and Medtr4g078885.1 can be assigned to the salt/heat induced group previously reported (Figure 7), however the statistical analysis revealed that the increase under the two abiotic stresses is not significant (Supplementary Table S9). Medtr4g078885.1 shows a decrease in expression after one day of cold treatment, which is statistically significant (Figure 7 and Supplementary Table S9). This expression pattern is reminiscent of the one described previously in the leaves (Figure 6) and suggests that this dirigent-like protein might participate in a biochemical branch of alfalfa secondary metabolism controlling the response to multiple stresses.

### 2.6. CesA Genes Expression in Alfalfa under Temperature Stresses: Major Differences in Roots vs. Leaves

Two main differences are obvious and significant in roots and leaves of alfalfa plants subjected to temperature stress and involve the *CesA* genes.

**Figure 8:**
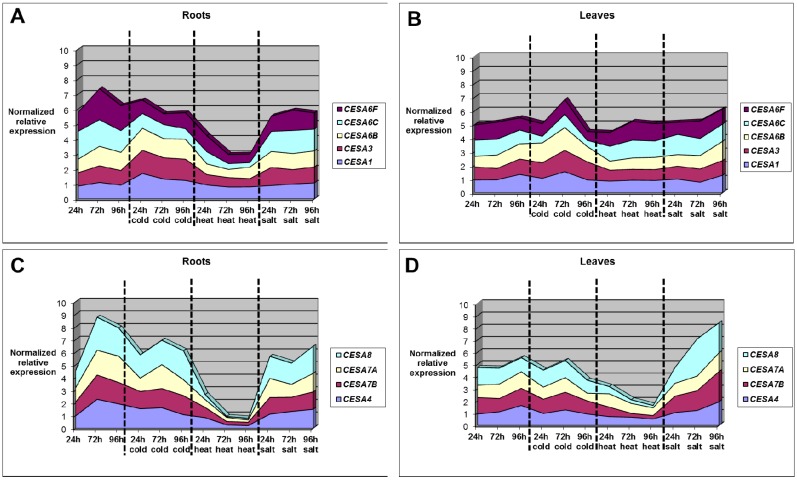
Graphs showing the change in expression of primary and secondary *CesA*s in roots (**A**,**C**) and leaves (**B**,**D**) of alfalfa plants subjected to different abiotic stresses. The data correspond to the values reported in Supplementary Tables S5 and S7. The control condition and the different stress treatments are boxed. The standard error of the mean is not represented in the graphs.

(1) Primary *CesA*s do not show significant changes in expression under cold/heat stress in the roots, with the exception of *CesA6F* (which decreases as heat stress continues), while in the leaves their expression peaks at 72 h of cold stress, but is stable under heat stress (Figure 8, panels A and B).

(2) Heat stress induces a decrease in secondary *CesA*s expression in both roots and leaves, as previously described in stems [21]. In the roots the decrease is however more important and gradual than the leaves (Figure 8, panels C and D).

Our results indicate that in alfalfa roots heat stress markedly reduces the expression of all the *CesA* genes belonging to the secondary cell wall clade, probably to ensure flexibility to the stressed root cell walls. In the leaves the down-regulation of secondary *CesA*s is less dramatic than the roots and the primary *CesA*s show a peak in expression at 72 h of cold stress. These genes might support cell primary growth under cold stress.

Taken together our results confirm literature evidences showing that roots are more sensitive to heat stress than aerial organs [47,52].

## 3. Materials and Methods

### 3.1. Identification of Cell Wall-Related Genes in Alfalfa

The identification of putative *CesA*s from *M. sativa* has been described in [21]. The identification of putative dirigent/dirigent-like proteins in *M. truncatula* was carried out by querying the database Phytozome ([53]; v10.2; Available online: http://phytozome.jgi.doe.gov/pz/portal.html), by using both the keyword “dirigent” and BLASTp searches with the *A. thaliana* protein sequences as query.

The phylogenetic tree of *M. truncatula* dirigent/dirigent-like proteins was built by aligning the full length amino acid regions of *M. truncatula*, *A. thaliana*, *Picea sitchensis*, *Oryza sativa*, *Hordeum vulgare* and *Triticum aestivum* protein sequences using MUSCLE [54]. The maximum-likelihood phylogenetic tree (bootstrap = 100) was obtained using PhyML [55]. The tree was visualized using iTOL ([56,57]; Available online: http://itol.embl.de/). The accession numbers used to build the tree are the following: AtDIR1, ABR46205.1; AtDIR10, AAU90058.1; AtDIR11, AAQ65106.1; AtDIR12, AEE82982.1; AtDIR13, AAP88352.1; AtDIR14, AEE82984.1; AtDIR15, AEE86966.1; AtDIR16, AAP37695.1; AtDIR17, CAB67637.1; AtDIR18, AEE83298.1; AtDIR19, AAO39937.1; AtDIR2, AAP37801.1; AtDIR20, AAU15178.1; AtDIR21, AEE34435.1; AtDIR22, AAU15153.1; AtDIR23, AAT71988.1; AtDIR24, AEE79355.1; AtDIR25, AAP49521.1; AtDIR3, AED95765.1; AtDIR5, AAQ65109.1; AtDIR6, AEE84795.1; AtDIR7, AAQ89609.1; AtDIR8, AEE75389.1; AtDIR9, AAR20779.1; AtDIR4, AEC07124.1; HvDIR1, AAA87042.1; HvDIR2, AAA87041.1; HvDIR3, AAB72098.1; TaDIR1, AAC49284.1; TaDIR2, AAM46813.1; TaDIR3, BAA32786.3; TaDIR4, AAR20919.1; PDIR25, ABR27721.1; PDIR26, ABR27722.1; PDIR28, ABR27724.1; PDIR29, ABR27725.1; OsDIR47, BAF27863.1; OsDIR48, BAF27866.1; OsDIR23, BAF26452.2; OsDIR5, BAF26451.1.

For the identification of *M. sativa* and *M. truncatula* class III peroxidases, the PeroxiBase database ([40]; Available online: http://peroxibase.toulouse.inra.fr/) was used. A total of 15 class III peroxidases were retrieved in *M. sativa* and 102 in *M. truncatula*. The phylogenetic tree of *Medicago* class III peroxidases was built as described above, by aligning the conserved amino acid region of *M. truncatula* and *M. sativa* protein sequences (namely the region encompassing the two conserved His and the eight conserved Cys; [38]). *M. sativa* gene accessions used to build the tree are the following: MsPrx01, AJ306689.1; MsPrx02, X90693; MsPrx03, X90694.1; MsPrx04, CO515178.1 (partial sequence); MsPrx05, CO512465.1 (partial sequence); MsPrx06, CO51624.1 (partial sequence); MsPrx07, CO515766.1 (partial sequence); MsPrx08, CO513054.1 (partial sequence); MsPrx09, CO512914.1 (partial sequence); MsPrx10, CO511956.1; MsPrx12, AJ306690.1; MsPrx13, CO513985.1 (partial sequence); MsPrx14, L36156.1; MsPrx15, L36157.1; MsPrx16, L36158.1.

Probe Set IDs for *M. truncatula* dirigent/dirigent-like proteins and class III peroxidases were retrieved at http://mtgea.noble.org/v3/. Available expression data deriving from electronic fluorescent pictographs (eFP) were obtained at http://bar.utoronto.ca/efpmedicago/cgi-bin/efpWeb.cgi and used for hierarchical clustering [33]. Hierarchical clustering was generated with Cluster 3.0 [58] using uncentered correlation similarity metric and complete linkage clustering method. The distance matrix was visualized using Java TreeView ([59]; Available online: http://jtreeview.sourceforge.net/).

Sequence alignments of the dirigent/dirigent-like proteins and class III peroxidases were performed using Kalign (Available online: http://www.ebi.ac.uk/Tools/msa/kalign/) and shading of conserved residues was done using BoxShade (Available online: http://www.ch.embnet.org/software/BOX_form.html). Prediction of the *M. truncatula* dirigent-like proteins 3D structure was performed using the Phyre2 server ([60]; Available online: http://www.sbg.bio.ic.ac.uk/~phyre2/html). Visualization of the Medtr1g054525.1 3D model was performed with SwissPdbViewer ([61]; Available online: http://www.expasy.org/spdbv/). The occurrence of XYLAT elements in the regions encompassing 2000 bp upstream of the ATG was predicted using PLACE-A Database of Plant Cis-acting Regulatory DNA Elements ([62,63]; Available online: http://www.dna.affrc.go.jp/PLACE/signalscan.html).

### 3.2. Salt, Cold and Heat Stress Treatments of Alfalfa Plants

*Medicago sativa* L. (variety “Giulia”) plants were grown for four weeks under controlled greenhouse conditions (*i.e.*, with a photoperiod of 13 h light/11 h darkness, *T*_min_ 20 °C, *T*_max_ 27 °C) [21], then subjected to the different stress treatments for 4 days. In particular, for the mild salt stress treatment, plants were supplemented with 100 mM NaCl; for the cold stress condition, plants were grown in incubators (providing the same light/dark cycle) set at a constant temperature of 5 °C; for the mild heat stress condition, alfalfa was grown at 28 °C/32 °C (night/day). At harvest the plants were 32 days old. Three independent biological replicates were analyzed per treatment (each replicate was composed of a pool of 15 plants). For each time point studied (0, 24, 72 and 96 h), controls consisting of plants grown without any treatment for 24, 72 and 96 h were kept for appropriate comparisons.

### 3.3. RNA Extraction and cDNA Synthesis

Alfalfa tissues were collected and processed as described in [21]. One hundred mg of finely-ground sample were weighed on a balance and total RNA was extracted using the RNeasy Plant Mini Kit with the on-column DNase I treatment (Qiagen, Leusden, The Netherlands). The integrity of the extracted RNA was checked with an Agilent Bioanalyzer (Santa Clara, CA, USA) (all the RINs were ≥8) and the purity/concentration measured using a NanoDrop ND-1000 spectrophotometer (Thermo scientific, Villebon-sur-Yvette, France) (A260/280 and A260/230 ratios between 1.9 and 2.2). Subsequently, 1 µg of extracted RNA was retro-transcribed using the Superscript II cDNA Synthesis kit (Invitrogen, Carlsbad, CA, USA), according to the manufacturer’s instructions.

### 3.4. Primer Design, Quantitative Real-Time PCR and Statistical Analysis

All the primers were designed using Primer3Plus [64] and analysed with OligoAnalyzer 3.1 (Available online: http://eu.idtdna.com/analyzer/Applications/OligoAnalyzer/). The primers for the *M. sativa CesA* genes, the reference genes EIF5A, PAB4, ADF1 and TFIIA, CAD and PAL have been previously reported [21]. The primers for the genes encoding dirigent-like proteins were designed on the identified *M. truncatula* sequences, namely Medtr4g078885.1 and Medtr7g093850.1; the primer for the dirigent-like protein orthologous to Medtr1g054525.1 was designed using the alfalfa EST GenBank accession EX525320.1 (Supplementary Table S10). The primers for the class III peroxidases analysed were designed on the *M. truncatula* genes named MtPrx13, MtPrx38 and MtPrx42 according to the PeroxiBase database. It should however be noted that for two of these barrel medic peroxidases, alfalfa orthologs are known, namely MtPrx38 is the ortholog of *M. sativa* MsPrx12, MtPrx42 of MsPrx16. For the sake of clarity, these peroxidases are referred to using the alfalfa nomenclature in the Figures and Tables showing the RT-qPCR results. For the alfalfa gene orthologous to MtPrx13, the barrel medic nomenclature was kept (*i.e.*, Prx13). The primer efficiencies can be found in Supplementary Table S10.

For qPCR analysis, 10 ng of cDNA were used as template; the reactions were set up in 384 well-plates and prepared using a liquid handling robot (epMotion, Eppendorf, Hambourg, Germany). The cDNA was amplified using the Takyon Low ROX SYBR MasterMix dTTP Blue Kit (Eurogentec, Liège, Belgium) on a ViiA 7 Real-Time PCR System (Thermo Fisher, Waltham, MA, USA) in a final volume of 10 µL.

The reactions were performed in technical triplicates and repeated on the above-mentioned three biological independent replicates. The PCR conditions consisted of an initial denaturation at 95 °C for 10 min, followed by 45 cycles of denaturation at 95 °C for 15 s, annealing/extension at 60 °C for 60 s. A melting curve analysis was performed at the end of the experiment to check the specificity of the amplified products. The targets displayed melt curves with one clear peak, with the exception of MsPrx16 in the leaves, where a second peak could be observed (Supplementary Figure S5). This gene was therefore not retained for analysis in these organs. The expression relative to the dirigent-like protein Medtr4g078885.1 was very low in the roots in all the conditions tested (*C*_t_ > 30), therefore this target was not included in the results (Supplementary Figure S6).

The RT-qPCR data were analyzed using the qBase^PLUS^ version 2.5 software (Biogazelle, Ghent, Belgium; [65]) and normalized taking into account the most stable reference genes among 4 tested. For the roots the reference genes are EIF5A, PAB4, ADF1 and TFIIA, for the stems TFIIA, GAPDH, PAB4 and EIF4A, for the leaves TFIIA, UBC13, PAB4 and EIF4A (chosen according to [21]). Data for the roots were normalized using PAB4/TFIIA, for the leaves eif4A/TFIIA and for the stems eif4A/PAB4.

The expression levels are here indicated as “Normalized relative expression”. A one-way ANOVA (with Tukey’s HSD post-hoc test) was performed on the log_2_ transformed calibrated normalized relative quantities (CNRQs), using IBM SPSS Statistics (version 19), after having checked the normal distribution of the variances with a Levene test. For gene expression values presenting variances not normally distributed, a Kruskal-Wallis analysis was performed. The hierarchical clustering was performed as described above.

## 4. Conclusions

The present study complements our previous results on the expression of *CesA*s, PAL, CAD, SuSy and a cellulose synthase-like gene in alfalfa stems under abiotic stresses [21]. Common, as well as different response patterns have been identified in roots and leaves of alfalfa plants. To summarise, two main features differentiate the response of alfalfa roots and leaves to abiotic stresses:

(1) In the roots, the differences in gene expression are more pronounced as compared to that in the leaves (Figure 5 and Figure 6 and Supplementary Tables S5–S8).
(2) While in the roots primary *CesA* genes are stable under cold stress, they peak at 72 h in the leaves. Under heat stress, primary *CesA*s do not show significant changes in expression, neither in roots nor in leaves (Figure 8, panels A and B). On the contrary, heat stress induces a decrease in secondary *CesA* expression both in roots and in leaves. The decrease is more marked and progressive in the roots (Figure 8, panels C and D).

In addition to these main results, we have here provided an *in silico* analysis of *Medicago* dirigent/dirigent-like proteins and class III peroxidases, and have identified a response to multiple stresses of the alfalfa dirigent-like protein orthologous to Medtr4g078885.1. Functional studies are needed to verify the role of this gene in the response to multiple stresses in alfalfa.

## Supplementary Materials

Supplementary materials can be found at http://www.mdpi.com/1422-0067/16/07/16104/s1.
