# Supplementary material for: Analysis of Cell Wall-Related Genes in Organs of Medicago sativa L. under Different Abiotic Stresses
Source: Int J Mol Sci. 2015 Jul 16;16(7):16104–24. doi: 10.3390/ijms160716104 (PMC4519941; doi:10.3390/ijms160716104)
Supplement: Supplementary file 1 [file ijms-16-16104-s001.pdf]

Supplementary Information

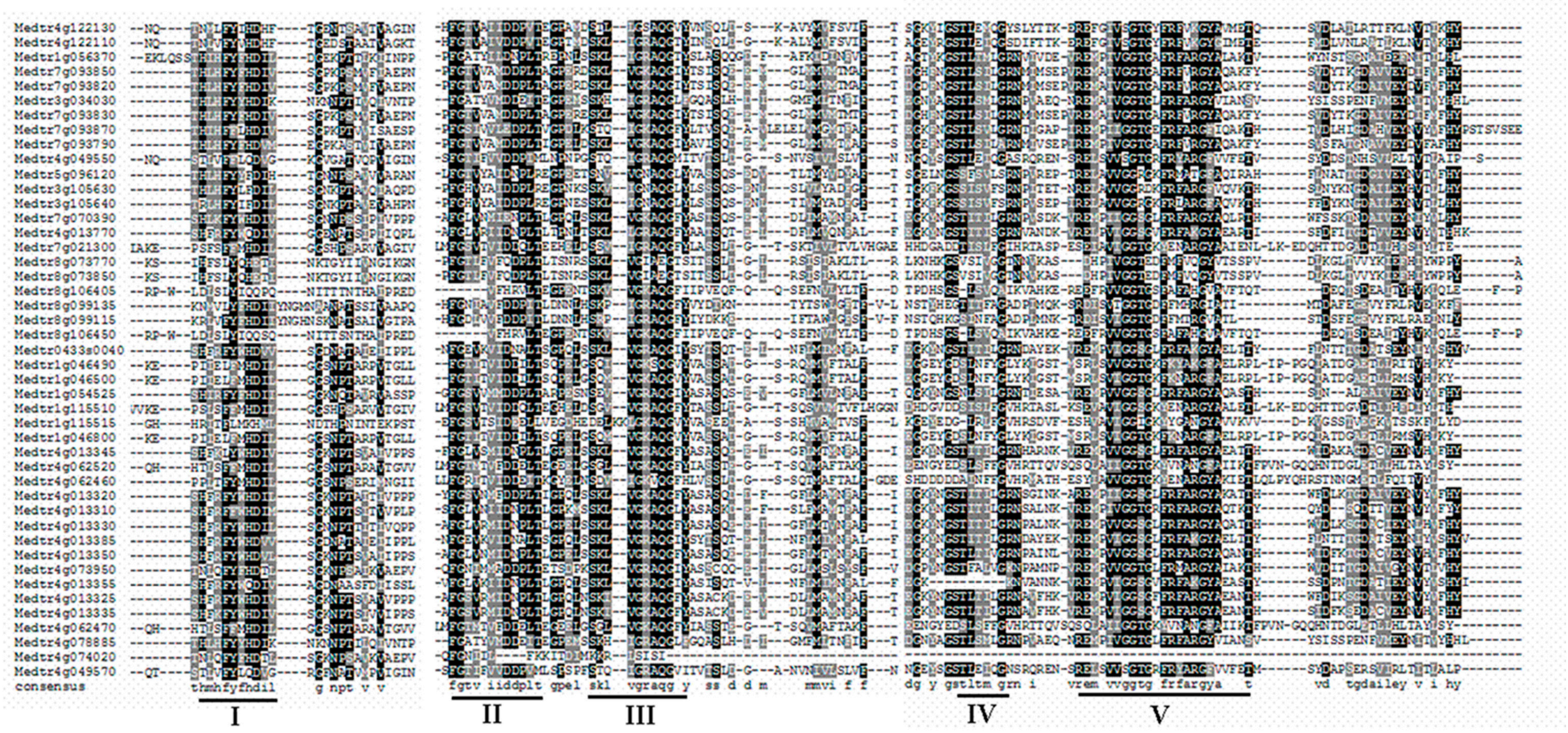

Figure S1. Partial alignment of *M. truncatula* dirigent and dirigent-like proteins showing the conserved five motifs (I–V) described in [29]. The alignment was performed with Kalign and the conserved residues were shaded using BoxShade (see Materials and Methods section).

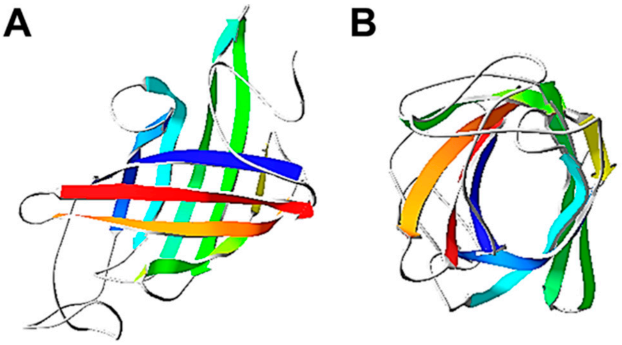

Figure S2. Putative 3D structure of the dirigent-like proteins Medtr1g054525.1 from *M. truncatula*. (**A**) lateral view showing the β-barrel; (**B**) the same structure viewed from the top to show the hydrophobic core (*i.e.*, the cavity where the substrate binds).

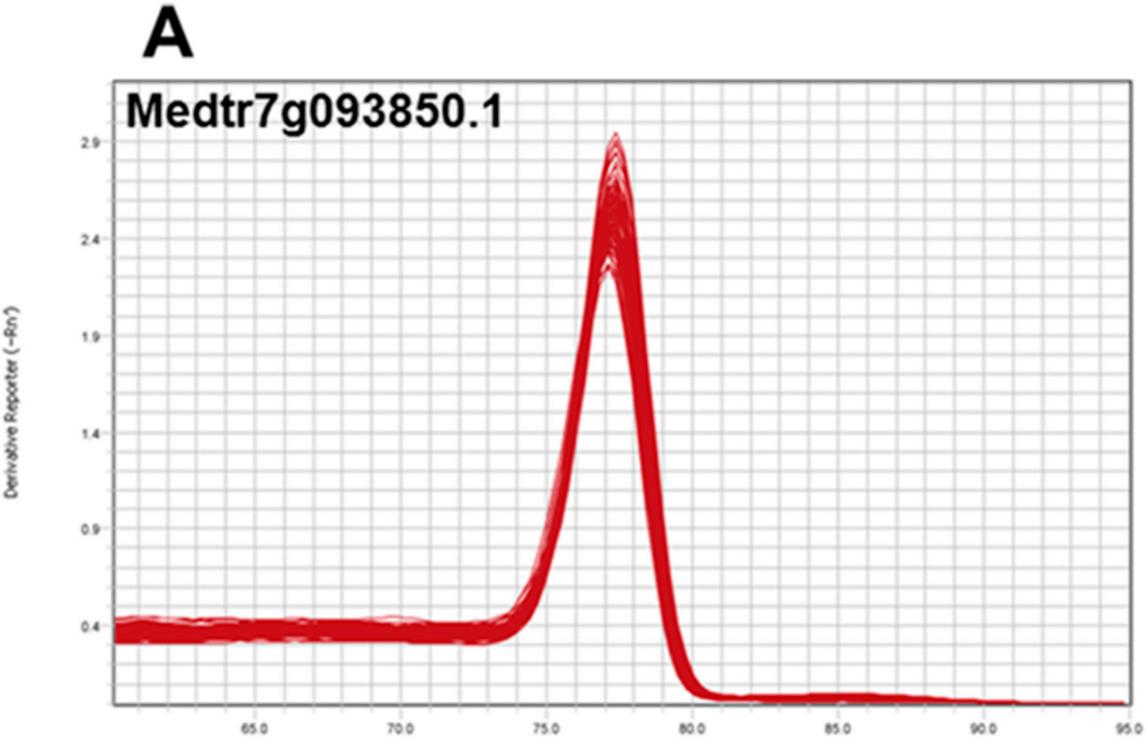

Figure S3. *Cont.*

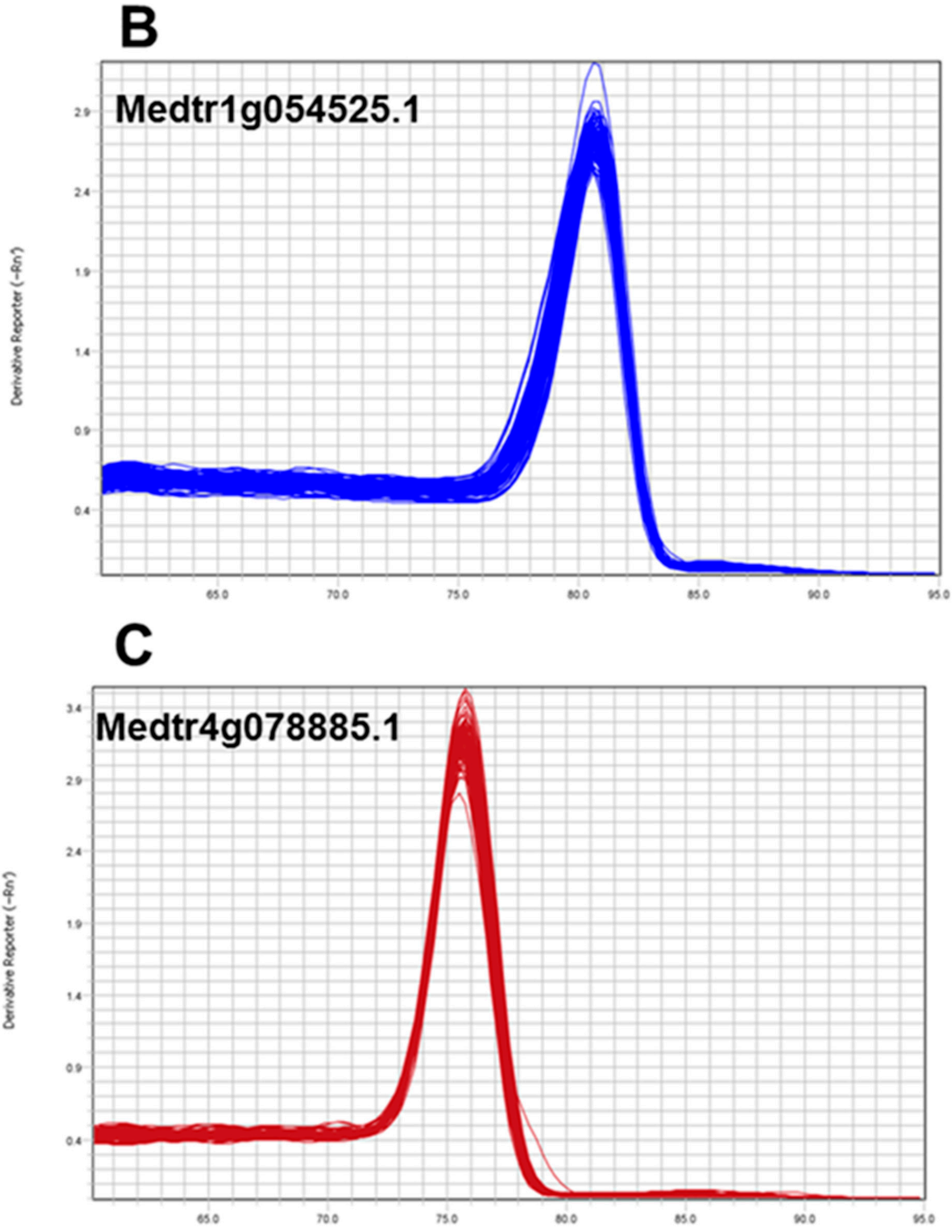

Figure S3. Melt curve analysis of the three genes coding for the dirigent-like proteins Medtr7g093850.1 (**A**), Medtr1g054525.1 (**B**) and Medtr4g078885.1 (**C**) in roots, stems and leaves of alfalfa.

```
MtPrx01     29 -LTPHFYDNV-CPQALPTIKSVVLHAILREKRIGASLLRLHFHDCFV-NGCDGSVLLDDT-PNF-T-GEKTALPNIN---
MtPrx02     25 -LTPNYYDRI-CPKALPIINSIVKQAIIREPRMGASLLRLHFHDCFV-NGCDGSVLLDDT-PTF-I-GEKTAFPNIN---
MtPrx03     27 -LTPDFYNDV-CPQALSIINTIVRQKLDFKPRMGAHLLRLHFHDCFV-NGCDGSVLLDDT-PNF-I-GEKTAKGNID---
MtPrx04     25 -LRKNFYKKS-CPQAEEIVKNITLQHVSSRPELPAKLIRLHFHDCFV-RGCDASVLLEST-AGN-T-AEKDAIPNL----
MtPrx05     50 -LELGFYTKS-CPKAEQIVANFVHEHIRNAPSLAAALIRMHFHDCFV-RGCDASVLLNST-N-Q-Q-AEKNAPPNL----
MtPrx06     27 -TRVGFYSST-CSQAESIVKSTVASHVNSDSSLAPGLLRMHFHDCFV-QGCDASVLVAGS-G-----TEKTAFPNL----
MtPrx07     26 -LEVGFYTYS-CGMAEFIVKDEVRKSFNKNPGIAAGLVRMHFHDCFI-RGCDASVLLDST-LSN-I-AEKDSPANKP---
MtPrx08     23 -LSLNYYEKS-CHDLEYIVLKTVTDATARDKTVPAALLRMHFHDCFV-RGCDASVLLNSK-GKN-K-AEKDGPPNI----
MtPrx09     28 -LTTDFYKSS-CPNLTKIVRKEVVKALKNEMRMGASLLRLHFHDCFV-NGCDGSILLDGG-DDF----EKSAFPNIN---
MtPrx10     29 -LDPSFYRDT-CPKVHSIIREVIRNVSKTDPRMLASLVRLHFHDCFV-LGCDASVLLNKT-DTI-V-SEQEAFPNIN---
MtPrx100    50 -LSFSYYRKS-CPQFESILHSKVKEWIKKDYTLAASLLRLHFHDCSV-RGCDGSILLNHE-G-----SERKSQVSK----
MtPrx107    25 -LAFDFYAAS-CPTAELFIRNAVSTSSSNDPSIPGKLLRMVFHDCFV-EGCDASLMLQGN-N-----TEQSDPGNR----
MtPrx11     29 -LDPSFYRNT-CPNVSSIVREVIRSVSKKDPRMLGSLVRLHFHDCFV-QGCDASVLLNKT-DTV-V-SEQDAFPNRN---
MtPrx110    25 -LSSTFYDST-CPNALSTIRTSIRTAISKERRMAASLIRLHFHDCFV-QGCDASILLDDS-STI-E-SEKSARPNVN---
MtPrx111    24 -LSPSFYSQT-CPFLYPIVFRVIFEASLTDPRIGASLIRLHFHDCFV-QGCDGSVLLNNT-NTI-V-SEQDALPNIN---
MtPrx114    25 -LAFDFYAAS-CPTAELFIRNAVSTSSSNDPSIPGKLLRMVFHDCFV-EGCDASLMLQGN-N-----TEQSDPGNR----
MtPrx116    16 -LQLGLYAFS-CQKAELIVPQVVQKRFNRDKSVIAALICMHFHDCFV-RGCDASLLIDST-NNT-T-AEKDSGTNE----
MtPrx12     40 -LNPSFYSKT-CPNVSSIVREVIRNVSKTDTRMLASLVRLHFHDCFV-QGCDASVLLNNT-ATI-V-SEQDAFPNRN---
MtPrx13     26 ELHAHYYDQT-CPQLDKIISETVLTASIHDPKVPARILRMFFHDCFI-RGCDASVLLDST-ATN-Q-AEKDGPPNI----
MtPrx14     24 -LTRGFYNNV-CPNVEQLVRSAVNQKFQQTFVTAPATLRLFFHDCFV-RGCDASILLATP-----K-AEREHPDDIS---
MtPrx15     29 -LDNSFYRDT-CPNVHSIVREVLRNVSKTDPRILASLIRLHFHDCFV-QGCDASILLNTT-STI-T-SEQTAFGNNN---
MtPrx16     29 -LSPDFYAKT-CPQLQSIVFQILEKVSKTDSRMPASIIRLHFHDCFV-QGCDASVLLNKT-STI-A-SEQDAGPNIN---
MtPrx17     29 -LDPYFYGKT-CPKLHSIAFKVLRKVAKTDPRMPASIIRLHFHDCFV-QGCDASVLLNNT-ATI-V-SEQDAFPNIN---
MtPrx18     41 -LSIGHYHST-CPDAEGIISQKVFAWVKKDPTLAPSIIRLHFHDCAV-RGCDGSILLNHV-G-----SERTAFASK----
MtPrx19     38 -LNMNYYLMS-CPFVEPVVKNIVNRALDNDPTLAAALIRMHFHDCFI-QGCDGSILLDST-KDN-T-AEKDSPANL----
MtPrx20     28 -LSPTFYSKT-CPTVSSIVSNVLTNVSKTDQRMLASLVRLHFHDCFV-LGCDASVLLNNT-ATI-V-SEQQAFPNNN---
MtPrx21     24 -LHVGFYSNT-CPQVESTVHDVVREAVLFDRTKAAVLLRLHFHDCFV-EGCDGSILINTTQN-----PEKTAFPHA----
MtPrx22     31 -LSKDYYYSS-CPKLFETVKCEVQSAISKETRMGASLLRLFFHDCFV-NGCDGSILLDDT-SSF-T-GEKTANPNKN---
MtPrx23     69 -LTSTFYSNT-CPSVSSIVRNVVQQALQNDPRITASLTRLHFHDCFV-NGCDASILLDQG-GNITL-SEKNAVPNNN---
MtPrx24     46 -LSSTFYGTR-CPRALYIIRREIIAAVSRDRRLGASLLRLHFHDCFV-QGCDASVLLKDT-PTF-Q-GEQNARPNAN---
MtPrx25     30 -LYPQFYDYS-CPQAQNIVKSILANAVAKEPRIAASLLRLHFHDCFV-KGCDASILLDNS-GSI-I-SEKGSNPNRN---
MtPrx26     29 -LDPSFYNST-CSNVDSIVRGVLTNVSQSDPRMLGSLIRLHFHDCFV-QGCDASILLNDT-ATI-V-SEQSAPPNNN---
MtPrx27     29 -LTPYFYRTT-CPDVFTIVRREVLNAINEEIRMAASLLRLHFHDCFV-NGCDASILLDGD-EDI----EKFATPNIN---
MtPrx28     25 -LVNNFYGTT-CPSLQTIVRREMTKAINNEARIGASILRLFFHDCFV-NGCDGSILLDDT-STF-T-GEKNAGPNKN---
MtPrx29     30 -LSENFYAKK-CPNVFKAVNSVVHSAVAREPRMGGSLLRLHFHDCFV-NGCDGSVLLDDT-PSN-K-GEKTALPNKD---
MtPrx30     25 -LSPSFYSQT-CPFLYPIVFRVIYEASHTDPRIGASLIRLHFHDCFV-QGCDGSVLLNNT-DTI-V-SEQDAFPNRN---
```

S5

```
MtPrx32      45 -LRPSFYLLHGCPLAVQTIRTAITTAVLKDPRLGASLLRLHFQDCFV-QGCDASVLLKDT-PTF-K-GEQNARPNAN---
MtPrx33      24 -LSSNFYFRT-CPLVLSTIKKEVISALINERRMGASLLRLHFHDCFV-QGCDASVLLDDT-SSF-R-GEKTAGPNAN---
MtPrx34      26 -LSKDFYCSS-CPELLSIVNQGVINAIKKETRIGASLLRLHFHDCFV-NGCDASILLDDT-SSF-I-GEKTAAANNN---
MtPrx35      30 -LDPNFYKDT-CPNVTSIVQSILANVSQTDPRILASFIRLHFHDCFV-QGCDASLLLNDS-DTI-V-SEQGALPNRN---
MtPrx36      20 -LSTDFYSTT-CSDVLSTIKREIDSAVGNEARMGASILRLHFHDCFV-QGCDASVLLDDT-SSF-T-GEKTAGANAN---
MtPrx37      26 -LCTDFYCNS-CPELLSIVNQGVVNALKKETRIGASLLRLHFHDCFV-NGCDASILLDDT-SSF-I-GEKTAAANNN---
MtPrx38      27 -LSPNHYANI-CPNVQSIVRSAVQKKFQQTFVTVPATLRLFFHDCFV-QGCDASVLVASS-GNN-K-AEKDHPENLS---
MtPrx39      27 -LSENFYGKT-CPKAVRTIRKAVQDAVMNERRMGASLLRLHFHDCFV-QGCDASALLDDT-SNF-T-GEKNAFPNAN---
MtPrx40      33 -LTLDYYAST-CPTVFDIVRKQMECSVLSEPRNAASVVRLHFHDCFI-QGCDGSILLDDT-YTL-K-GEKKAATNIH---
MtPrx41      25 -LVENFYVSS-CPNVELVVAQAVTNKFTQTITTGQATLRLFLHDCFV-EGCDASVMIASP--NG-D-AEKDAKENLS---
MtPrx42      27 -LSRHHYKNS-CPNVENIVREAVKKKFHQTFTTVPATLRLFFHDCFV-QGCDGSILVSST-PHN-R-AERDHPDNLS---
MtPrx43      25 -LFPNFYGRT-CPSLQTIVRREMTKAINNEARIGASILRLFFHDCFV-NGCDGSILLDDT-STF-T-GEKNAGPNKN---
MtPrx44      26 -LSPNFYAKT-CSNLQTIVRNEMIKVIQKEARMGASILRLFFHDCFV-NGCDASILLDDK-GTF-V-GEKNSGPNQG---
MtPrx45      45 GLSPQFYQFS-CPQANDIVMSVLEKAIAKDIRIAASLLRLHFHDCFV-QGCDASILLDDS-ATI-V-SEKNGGPNKN---
MtPrx46      30 -LYPQFYDES-CPKVEEIVKSVVAKAVTKEPRMAASLLRLHFHDCFV-KGCDASVLLDSS-GTI-I-SEKRSNPNRN---
MtPrx47      32 -LVMNYYKEA-CPQAEEIIKEQVKLLYKRHKNTAFSWLRNIFHDCAV-QSCDASLLLTST-RRS-L-SEQ-EHDRSF---
MtPrx48      42 -LSYGFFAQT-CPNLENIVRKHLTKVFKSDNGQAPGLLRIFFHDCFV-QGCDGSVLLDGK-----P-GERDQPQNGG---
MtPrx49      27 -LTTNYYDYT-CPNALSTIKSVVKAAVQRENRMGASLLRLHFHDCFV-NGCDGSILLDST-PSM-D-SEKNANPNIN---
MtPrx50      29 -LQVGFYSYS-CPSAEAIVRSAIDKAVSLNPGIGAGLIRMHFHDCFV-RGCDASVLLAST-PGN-PIAEKDNFINNP---
MtPrx52      27 -LYYNFYNRT-CPNLNKIVKDNILSAIANDSRIAASLLRLHFHDCFV-NGCDGSVLLDDT-DTL-K-GEKNALPNKN---
MtPrx53      27 -LQPGFYAKS-CPKAEQIVLKYVHDHIPNAPSLAAALIRLHFHDCFV-KGCDASVLLNST-QTN-Q-AEKDAIPNL----
MtPrx54      28 -LSTNFYSKT-CPKLSSIVQRQVQSAISKEARIGASILRLFFHDCFV-NGCDGSILLDDT-SNF-T-GEKNALPNKN---
MtPrx55      24 -LSVNYYEHT-CPQVESIVAGAVHKATMNDKTVPSALLRMHFHDCFV-RGCDGSVLLKTK-GKN-K-AEKDGPPNI----
MtPrx56      27 -LQVNYYSKS-CPKAEEIIKQQVNELYNKHGNTAISWVRNLFHDCIV-KSCDASLLLETV-HGV-V-SEQ-TAERSF---
MtPrx57      25 -LVNNFYGTT-CPSLQTIVRNKMTSAIKTEPRIGASILRLFFHDCFV-NGCDGSILLDDT-ATF-T-GEKNAAPNKN---
MtPrx58      27 -LYYNFYIRT-CPNLNRIVKNNILSAIANDSRIAASLLRLHFHDCFV-NGCEGSVLLDDT-DTL-K-GEKNALPNKN---
MtPrx60      36 -LQWHYYHNT-CRDAEVYVRHQVKIHWDQDKSITAKLLRLVYSDCFV-NGCDASILLDEGPN-----PEKRAPQNR----
MtPrx61      31 -TNEMYYDNT-CPNALVAIQQAVQNAVLGEARIGASLLRLHFQDCFV-QGCDGSVLLDDT-SSF-K-GEKNSLQNAN---
MtPrx63      25 -LIDNFYDQT-CPCLQTIVRNTMTSAIKKEARIGASILRLFFHDCFV-NGCDGSILLDDT-DTF-I-GEKKAQPNNN---
MtPrx64      77 -LEYDFYRDS-CPHAEHIVRSTLHLLYKTNPALVPALIRLVFHDCFI-QGCDASILLDND-EYI-D-SEKDSPXND----
MtPrx65      30 -LKYGFYKTT-CSSVEAIVRRAVNKAVSLNPGIAAGLIRMHFHDCFV-RGCDGSVLLDSI-PGI-Q-SERDHPANNP---
MtPrx66      21 -LKTGFYSNS-CPTAESIVRSTVVSYFNKDPTIAPGLLRLHFHDCFV-QGCDGSILIAGS-S-----SERSALPNL----
MtPrx67      24 -LSSTFYDST-CPNALSTIRTVIRTAVSKERRMAASLIRLHFHDCFV-QGCDASILLDDT-STI-E-SEKSALPNIN---
MtPrx70      25 -LTPNYYDRI-CPKALPVIKSIVKQAIIREPRMGASLLRLHFHDCFV-NGCDGSVLLDDT-PTF-I-GEKTAFPNIN---
MtPrx71      29 -LDPAFYSKT-CPNLNSIVREIVRNFTKIEPRMPAILIRLHFHDCFV-QGCDASILLNNT-ATI-V-SELQALPNIN---
MtPrx74      42 -LRVDYYAKS-CPQVEQLVGSVTSQQFKQSPVSGPATIRLLFHDCFV-EGCDASILIASK-PGS-KELAEKDAEDNK---
```

```
MtPrx75     36 -LDYDFYRLS-CPRIERIVRKHLEDVFEKDSGQAPGILRLFFHDCFS-QGCDASILLNGV-----D-GEDDEKQHDANFA
MtPrx76     24 -LSSTFYDST-CPNALSTIRTSIRTAVSKECRMAASVIRLHFHDCFV-QGCDASILLDDS-PTI-E-SEKNALPNIN---
MtPrx78     21 -LRFGFYSFT-CPQVEEIVGEVVQSYYQRDITIAGALIRLHYHDCIV-NGCDASILIDSR-VNR-T-AEKDAKPNK----
MtPrx79     34 -LTLDYYKQT-CPQFQQIIQQTVTSKQIQSPTTAAATLRLFLHDCLLPNGCDASVLLSST-PFN-K-AERDNDINLS---
MtPrx81     27 -LTTNYYDYT-CPNALSTIRSVVEAAVEKENRMGGSLLRLHFHDCFV-NGCDGSILLDST-PSM-D-SEKNANPNIN---
MtPrx82     60 -LRYNFYKDS-CPEAENIVRSAVTDIYSDHRDLAPSLLRLFFHDCFI-QGCDASLLLE-D-RNI-S-YEKQAIPNQ----
MtPrx83     73 -LKQGFYSES-CPTAEKIVADALVEITKTNPNAIANIIRLQFHDCFV-VGCDSSVLLDYT-PTG---DKVEKSSMLN-GQ
MtPrx84     28 -LSTNFYSKT-CPKLSTTVKSTLQTAISKEARMGASILRLFFHDCFV-NGCDGSILLDDT-SSF-T-GEKNANPNRN---
MtPrx85     38 -LGTDIYQYS-CPEAEAIIFSWVEQAVSSDPRMAASLLRLHFHDCFV-NGCDASVLLDDT-ENF-V-GEKTAAPNVN---
MtPrx86     25 -LSSNYYDEI-CPQALPIIKSVVKQAIIREPRMGASLLRLHFHDCFV-NGCDGSILLDDT-PTF-I-GEKTAIPNIN---
MtPrx87     30 -LKYGFYKTT-CPSAEAIVRRAVNKAVSLNPGITAGLIRMHFHDCFV-RGCDGSVLLDSI-PGI-R-SERDHPANNP---
MtPrx88     22 -LKVGFYSSS-CPRAELIVRQVVERSFNQDRSMTAALLRMHFHDCFV-RGCDASILIDSK-KGN-E-SEKAARANL----
MtPrx89     20 -LELGFYASS-CRKAESIVKQVVQKRFNRDKSITAALLRMHFHDCFV-RGCDASLLIDST-KNN-I-SEKDTGAND----
MtPrx90     26 -LVHEYYKEK-CPLAEDIVRHNVAVAVLKDPRLAASLLRLHFHDCFV-MGCDASVLLDSV-EGM-T-SEKQAGPNVN---
MtPrx91     23 -LIPNYYQKS-CPKFEDIVKQTVTDKQKTTPSTAGAALRLFFSDCMI-GGCDASVLVSSN-SFN-K-AERDADINLS---
MtPrx92     26 -LSYNYYEKT-CPDVEFIVAKTVKAATASDKTVPAALLRMHFHDCFI-RGCDASVLLNSK-GSN-K-AEKDGPPNA----
MtPrx93     29 -LSYNYYKNS-CPNLESLVERELMSVFMTDIRAPSAFLRLMFHDCQV-QGCDASILLDTI-YAT-Q-SSEIASSGNF---
MtPrx94     40 -LRPGFYSKT-CPKAETIVRDVMRKALIREPRSVASVMRLQFHDCFV-NGCDGSVLLDDT-PTM-L-GEKLALSNIN---
MtPrx95     28 -LRPGYYSKT-CPQAETTVRDVMRNALKKEPRSVASVMRFQFHDCFV-NGCDGSVLLDDT-PTM-L-GEKLALSNIN---
MtPrx97     25 -LVNNFYGRT-CPSLQTIVRREMANAIKTEARIGASILRLFFHDCFV-NGCDGSILLDDT-ATF-T-GEKNAAPNKN---
MtPrx98     28 -LSTSFYSSS-CPKLSSTVQSTVQSAISNEARMGASILRLFFHDCFV-NGCDGSILLDDT-SNF-T-GEKNANPNRN---
consensus  241  l   fy  t Cp l  ivk  v  ai  e rigasllrlhfhDCfv ngCdaSvlld t  t    gek a pn n
```

```
MtPrx01    100 -SIR--GFSVVDEIKAAVIKV--CKGPVVSCADILATAARDSV-AILGG-PQFFYNVLLGRRDARTASKAAA-N-A-N-L
MtPrx02     96 -SIR--GFEVVDQIKEAVTKA--CKRDVVSCADILAIAARDSV-AILGG-KQYWYQVLLGRRDSRFASRDAA-N-T-N-L
MtPrx03     98 -SLK--GFMLVDIIKAELDKA--CKGPVVSCADILAVIARDSV-SILGG-PSYWYQVLLGRRDARNASMKDA-E-S-Y-L
MtPrx04     95 -SLA--GFDVIEDIKEALEEK--CPG-IVSCADILTLATRDAF-K--NK-PN--WEVLTGRRDGTVSRSIEALI-N---I
MtPrx05    119 -TVR--GFDFIDRIKSLVEAE--CPG-VVSCADIIALSARDSI-AATGG-PY--WKVPTGRRDGVVSNLLEANQ-N---I
MtPrx06     94 -GLR--GFEVIEDAKTKLEAA--CPG-VVSCADIVALAARDSV-VLSGG-LS--WQVPTGRRDGRVSQASDV-N-N---L
MtPrx07     97 -SLR--GFEVIDNAKAKLEEE--CKG-IVSCADIVAFAARDSV-ELAGG-LG--YDVPAGRRDGKISLASDT-R-T-E-L
MtPrx08     93 -SLH--AFYVIDEAKKALEAK--CPG-VVSCADILALAARDAV-YLSGG-PK--WNVPKGRKDGRTSKASET-R-Q---L
MtPrx09     97 -SVR--GFDVVDTIKTAVESA--CSG-VVSCADILAIAARDSV-LLSGG-PS--WSVMLGRRDGTISNGSLA-N-V-V-L
MtPrx10    100 -SLR--GLDVVNQIKTAVEKA--CPN-TVSCADILALSAQISS-ILAIG-PN--WKVPLGRRDGLTANQSLA-N-Q-N-L
MtPrx100   117 -TLR--GYEIIDDIKAEVEKQ--CPK-TVSCADILTTASRDAT-VELGG-PY--WSVPYGRKDGKVSIDKEA-E-M---V
MtPrx107    92 -SVG--GFSVIETAKRVLEMF--CPG-TVSCADIIALAARDAV-EIAGG-PR--VQIPTGRRDGMVSIASNV-RPN---I
MtPrx11    100 -SLR--GLDVVNQIKTAVEKA--CPN-TVSCADILALSAELSS-TLAIG-PD--WKVPLGRRDGLTANQLLA-N-K-N-L
MtPrx110    96 -SVR--GFEIIDKAKSEVEKV--CPG-VVSCADILAVAARDAS-FAVGG-PS--WTVKLGRRDSTTASKSLA-N-T-D-L
MtPrx111    95 -SLR--GLDVVNQIKTAVENE--CPA-TVSCADILTIAAQVAS-VLGGG-PS--WQIPLGRRDSLTANQALA-N-Q-N-L
MtPrx114    92 -SVG--GFSVIETAKRVLEMF--CPG-TVSCADIIALAARDAV-EIAGG-PR--VQIPTGRRDGMVSIASNV-RPN---I
MtPrx116    86 -CVR--GYDLIDNVKEAIKVV--CPL-TVSCADIVALATRDVV-PLSGG-PT--YGVPTGRLDGLVSN-NEV---N---I
MtPrx12    111 -SLR--GLDVVNQIKTAVEKA--CPN-TVSCADILALAAELSS-TLSQG-PD--WKVPLGRRDGLTANQSLA-N-Q-N-L
MtPrx13     97 -SVR--SFYVIDEAKAKLELA--CPG-VVSCADILALLARDVV-AMSGG-PY--WKVLKGRKDGRVSKASDT-A-N---L
MtPrx14     92 -LAG-DGFDTVVKAKAAVDRDPKCRN-KVSCADILALATRDVV-NLAGG-PF--YNVELGRRDGRVSTIASV-Q-R-S-L
MtPrx15    100 -SIR--GLDVVNQIKTAVENA--CPN-TVSCADILALAAEISS-VLANG-PD--WKVPLGRRDSLTANLTLA-N-I-N-L
MtPrx16    100 -SLR--RLDVINQIKTEVEKV--CPN-KVSCADILTLAAGVSS-VLSGG-PG--WIVPLGRRDSLTANQSLA-N-R-N-L
MtPrx17    100 -SLR--GLDVINQIKTKVEKA--CPN-RVSCADILTLASGISS-VLTGG-PG--WEVPLGRRDSLTANQSLA-N-Q-N-L
MtPrx18    108 -TLR--GFQLIDEIKAELERR--CPR-TVSCADILTAATRDAT-ILAGG-PF--WEVPFGRKDGKISIAKEA-N-L---V
MtPrx19    108 -SLR--GYEVIDDIKDELENR--CPG-VVSCADILAMAATEAV-FYAGG-PV--YNIPKGRKDGRRSKIEDT-R-N---L
MtPrx20     99 -SLR--GLDVVNQIKTAIESA--CPN-TVSCADILALAAQASS-VLAQG-PS--WTVPLGRRDGLTANRTLA-N-Q-N-L
MtPrx21     92 -GVK--GFEVIERAKAQLEAS--CPG-VVSCADIVALAARDAI-VMANG-PA--YQVPTGRRDGFVSDKSLA-G-N---M
MtPrx22    102 -SAR--GFEVIDKIKSAVEKV--CPG-AVSCADILTITARDSV-EILGG-PT--WDVKLGRRDARTASKSAA-N-N-D-I
MtPrx23    141 -SAR--GFDVVDKIKTSVENS--CPS-VVSCADILALAAEASV-SLSGG-PS--WNVLLGRRDGLIANQSGA-N-T-S-I
MtPrx24    117 -SLR--GFEFIDSLKAKIEAV--CPN-VVSCADILAVAARDSV-ATLGG-PI--WGVRLGRRDSTTANFNAA-N-S-D-L
MtPrx25    101 -SAR--GFEVIDEIKYALEKE--CPH-TVSCADILAIAARDST-VLAGG-PN--WEVPLGRRDSLGASLSGS-N-N-N-I
MtPrx26    100 -SIR--GLDVINQIKTAVENA--CPN-TVSCADILALSAEISS-DLANG-PT--WQVPLGRRDSLTANNSLA-A-Q-N-L
MtPrx27     98 -SAR--GFEVIDRIKSSVESS--CSG-VVSCADILAIVARDSV-HLSGG-PF--WYVQLGRRDGLVSNKTLA-N-N-A-I
MtPrx28     96 -SAR--GFEVIDAIKTSVEAA--CSA-TVSCADILALATRDGI-ALLGG-PS--WVVPLGRRDARTASQSAA-N-S-Q-I
MtPrx29    101 -SLR--GFEVIDAIKSKVESV--CPG-VVSCADIVAIAARDSV-VNLGG-PF--WKVKLGRRDSKTASLNDA-N-S-GVI
MtPrx30     96 -SLK--RLDVVNKIKTAVEEE--CPN-TVSCADILTIAAEVSS-ILGGG-PS--WPIPLGRRDSLTANITLA-N-Q-N-L
```

S8

```
MtPrx32   117 -SLR--GYEIIESVKAQLELL--CPN-VVSCADILALAARDSV-VALGG-PN--WLVRLGRRDSTTADFNAA-N-S-D-L
MtPrx33    95 -SLR--GFDVIDKIKSEVEKL--CPN-TVSCADILAVAARDSV-VALGG-LS--WTVQLGRRDSTTASFGLA-N-S-D-L
MtPrx34    97 -SAR--GFNVIDDIKANVEKA--CPG-VVSCADILTLAARDSV-VHLGG-PS--WNVGLGRRDSITASRSDA-N-N-S-I
MtPrx35   101 -SIR--GLDIVNQIKAAIELA--CPS-VVSCADILALGANVSS-VLALG-PD--WEVPLGRRDSFNANQSLA-N-S-S-L
MtPrx36    91 -SLR--GFDVIDTIKTELESL--CPN-TVSCADILSVAARDSV-VALGG-PS--WTVQLGRRDSITASLSLA-N-S-D-L
MtPrx37    97 -SAR--GFNVIDDIKASVEKA--CPK-VVSCADILALAARDSV-VHLGG-PS--WDVGLGRRDSITASRSDA-N-N-S-I
MtPrx38    98 -LAG-DGFDTVIKAKAALDAVPQCRN-KVSCADILALATRDVI-NLAGG-PS--YTVELGRFDGLVSRSSDV-N-G-R-L
MtPrx39    98 -SLR--GFELIDDIKSQLEDM--CPN-TVSCSDILALAARDGV-AELGG-QR--WNVLLGRRDSTTANLSEA-N-T---L
MtPrx40   104 -SLK--GFEIIDQIKNFVESE--CPG-VVSCADILTIAARDAV-ILVGG-PY--WDVPVGRKDSVTASFELA-N-T-N-L
MtPrx41    95 -LPG-DGFDTVIKAKQAVESV--CPG-VVSCADILAIATRDVI-ALLGG-PS--FSVELGRRDGLNSKASNV-E-A-N-L
MtPrx42    98 -LAG-DGFDTVIQAKAAVIAVPLCQN-KVSCADILAMATRDVI-ALAGG-PY--YEVELGRFDGLRSKDSDV-N-G-K-L
MtPrx43    96 -SAR--GFEVIDAIKTSVEAA--CSA-TVSCADILALATRDGI-ALLGG-PS--WIVPLGRRDARTASQSAA-N-T-Q-I
MtPrx44    97 -SAR--GFEVIDTIKTSVETA--CKA-TVSCADILALATRDGI-ALLGG-PS--WAVPLGRRDARTASQSAA-N-S-Q-I
MtPrx45   117 -SVR--GFEVIDEIKSKLEQA--CPR-TVSCADIVALAAKGST-VLSGG-PN--WELPLGRRDSKTASLRGS-N-K-N-I
MtPrx46   101 -SAR--GFEVIEEIKSAVEKE--CPQ-TVSCADILTLAARDST-VLTGG-PS--WDVPLGRRDSLGASISGS-N-N-N-I
MtPrx47   102 -GLR--NFRYIDTIKEAVERE--CPG-VVSCSDILVLSAREGI-VSLGG-PY--IPLKTGRRDGRKSRVDLL-E-A-Y-L
MtPrx48   110 MRTE--ALKTIDDIRALVHKE--CGR-IVSCADITVLAGREAV-FLSGG-PN--FPVPLGRKDGTSFSIKGT-S-N---L
MtPrx49    98 -SAR--GFEVVDEIKDAVIKA--CGKPVVSCADILAIAARDSV-VALGG-PS--WKVKLGRRDSKTASRADA-D-SGN-I
MtPrx50   101 -SLH--GFEVIDEAKAQLEVV--CPQ-TVSCADILTFATRDSILKLSGGTIN--YDVPSGRRDGRVSISDEV-P-K-N-I
MtPrx52    98 -SIR--GFDVIDKIKSDLENA--CPS-TVSCADILTLAARDAV-YQSKG-PF--WAVPLGRRDGTTASESDA-N---N-L
MtPrx53    97 -TLR--GYEFIDTIKSLVEKE--CPG-VVSCADILTLTARDSI-HAIGG-PY--WKVPTGRRDGIISKAADTFT-S---L
MtPrx54    99 -SVR--GFDVIDNIKTAVENV--CPG-VVSCADILAIAATDSV-AILGG-PT--WNVKLGRRDATTASQSDA-N-T-A-I
MtPrx55    94 -SLH--AFYVIDNAKKALEAV--CPG-VVSCADILALAARDAV-TLSGG-PN--WEVPKGRKDGIISKATET-R-Q---L
MtPrx56    97 -GMR--NFKYVSTIKAALEKE--CPL-TVSCADIVALSARDGI-ARLGG-PN--FEMKSGRKDSKESYVKVV-E-Q-F-I
MtPrx57    96 -SAR--GFEVIDTIKTSVEAS--CNA-TVSCADILALAARDGV-FLLGG-PT--WVVPLGRRDARTASQSAA-N-S-Q-I
MtPrx58    98 -SLR--GFDIIDKIKSDLEYA--CPN-TVSCADILTLAARDAV-YQSRG-PF--WAVPLGRRDGTTASESEA-N---N-L
MtPrx60   104 -GLG--GFVLIDRIKTVLESR--CPG-IVSCADILHLAARDAA-KMAGA-PG--YPIFTGRKDGMKSDAASV---D---L
MtPrx61   102 -SLR--GFELIDDIKSTLETM--CPN-VVSCADILTVAARDAV-VLLGG-QS--WNVPLGRRDSTTASLDAS-N-S-D-I
MtPrx63    96 -SVK--GFEVIDNIKNSVEAS--CNA-TVSCADILALAARDGV-VLLGG-PS--WTVPLGRRDARTANQSAA-N-S-Q-I
MtPrx64   147 -SLK--GFDVIETIKAKLEEA--CPG-VVSCADILVLAARDSV-VLAGG-PF--YPLNPGRRDGSNSFADIATD-E---L
MtPrx65   101 -SLR--GFEVINEAKAQIEAA--CPK-TVSCADILAFAARDSARKVSGGRID--YSVPSGRRDGRVSIFDEV-T-Q-N-L
MtPrx66    88 -GLR--GFEVIDNAKSQIEAI--CPG-VVSCADILALAARDAV-DLSDG-PS--WPVPTGRKDGRISLSSQA-S-N---L
MtPrx67    95 -SVR--GFEVIDKAKANVEKV--CPG-VVSCADIVAVAARDAS-FAVGG-PS--WTVKLGRRDSTVASKSQA-N-S-D-L
MtPrx70    96 -SIR--GFEVVDQIKAAVTKA--CKRDVVSCADILAIAARDSV-AILGG-KQYWYQVLLGRRDSRFASRDAA-N-T-N-L
MtPrx71   100 -SIR--GLQVVNRIKTDVEKA--CPN-TVSCADILALAARISS-VLSKG-PG--WIVPLGRRDSLTANRTLA-N-Q-N-L
MtPrx74   114 -DLRMEGFETIRKAKEVVEKK--CPT-VVSCADILAIAARDFV-HLAGG-PY--YQVKKGRWDGKISMASRV-G-S-N-I
```

```
MtPrx75     107 LREE--ALQTIENIRAIVRKQ--CPR-VVSCADILVIAAREAV-RQFGG-PD--IDVPLGRKDSLNFSVNSP-D-N---L
MtPrx76      95 -SVR--GFEIIDKAKSEVEKI--CPG-VVSCADILAVAARDAS-FAVGG-PS--WTVKLGRRDSTTASKSLA-N-T-D-L
MtPrx78      91 -TLR--GFNLIDDIKSNLEQQ--CPQ-TVSCADILTIATRDAI-ALSNG-PT--YNVLTGRRDGLVSNGFNV---T---I
MtPrx79     106 -LPG-DSFDLIVRIKTALELS--CPN-TVSCSDILATATRDLL-IMLGG-PH--YNVYLGRRDGRASVSSFV-D-G-F-L
MtPrx81      98 -SAR--GFEVVDEIKDAVIKA--CGKPVVSCADILAVAARDSV-VALGG-PS--WKVKLGRRDSKTASRADA-D-SGN-I
MtPrx82     129 -TLK--GFDKVDLIKEEVEQA--CPG-VVSCADILALAARDSV-FLGGG-PF--YPVLTGRRDSLQSFFQEAAD-Q---I
MtPrx83     145 -LLK--GTDFIDDIKAKLEEQ--CPG-IVSCTDTIAFSVNEGM-FLSGL-PR--TAPLGGRRDALYSLASIAED-D-N-L
MtPrx84      99 -SAR--GFDVIDNIKTAVENV--CPG-VVSCADILAIAAADSV-AILGG-PT--WNVKLGRRDAKTASQSAA-N-T-A-I
MtPrx85     109 -SLR--GFDVINEIKSELEVV--CPQ-TVSCADILATAARDSV-LLSGG-PT--WEVQMGRKDSITASKAGA-N-N-N-I
MtPrx86      96 -SLR--GFEVVDQIKAAVIKA--CKRPIISCADILAIAARDSV-AILGG-HKYWYQVLLGRRDSRFASRDAA-N-I-N-L
MtPrx87     101 -SLR--GFEVINEAKAQIEAA--CPK-TVSCADILAFAARDSARKVSGGRID--YSVPSGRRDGRVSIFDEV-T-Q-N-L
MtPrx88      92 -TVR--GYNLIDEIKRILENA--CPS-TVSCADIISLATRDSV-VLAGG-PS--YNVPTGRRDGLVSTVNDV---H---L
MtPrx89      90 -SVR--GYDLIDDVKEAIEAA--CPS-TVSCADIVALATRDAV-ALSGG-PK--YNIPTGRRDGLIANRDDV---D---L
MtPrx90      97 -SLR--GFEVIDKIKYLLEKE--CPL-TVSCADILAMVARDAV-ELRGG-PR--WEVWLGRKDSLESSFSGA-N-L-F-I
MtPrx91      94 -LSG-DGFEVVTRAKNMLELE--CPG-VVSCADILAAAARDLV-VSVGG-PF--YELDLGRRDSLESKSIDA-E-N-K-Y
MtPrx92      96 -SLH--AFFIIDNAKKALEAA--CPG-VVSCADILAFAARDAV-FLSGG-PS--WDIPKGRKDGRISKASET-I-Q---L
MtPrx93     100 -AIR--NRETINDIKSVLEEE--CPG-QVSCADIIVLAAKVSV-SLSGG-PS--IQVPFGRKDSRTSSSKEA-D-A-K-L
MtPrx94     111 -SLR--SFEVVDEVKEALEKA--CPG-VVSCADIIIMASRDAV-ALTGG-PD--WEVRLGRLDSLTASQEDS-D-N-I-M
MtPrx95      99 -SLR--SFEVVDEVKEALEKA--CPG-VVSCADIIIMASRDAV-ALTGG-PD--WEVRLGRLDSLTASQDNS-S-N-I-M
MtPrx97      96 -SAR--GFEVIDTIKTSVEAS--CNA-TVSCADILALAARDGV-FLLGG-PT--WVVPLGRRDARTASQSAA-N-S-Q-I
MtPrx98      99 -SAR--GFDVIDNIKTAVENV--CPG-VVSCADILAIAAADSV-AILGG-PT--WNVKLGRRDAKTASQSAA-N-T-A-I
consensus   321  sir  gfevid ik  ve    Cp  vvSCaDilalaardsv  l gg p   w vplGRrDa  a  s a n     l
```

```
MtPrx01    169 PSPTFNFSQLISNFKSQGLNVKDLVALSGGHTIGFARCTTFRNRIY----N----------E-TNIDPIFAASLRK--TC
MtPrx02    165 PPPFFNFSQLITNFKSHGLNLKDLVVLSGGHTIGFSKCTNFRDRIF----N----------D-TNIDTNFAANLQK--TC
MtPrx03    167 PSQLFNLSQLVSNFESQGLNLKDLVALSGAHTIGMAKCSTFRERIY----N----------D-TNIDPDFATCLQD--NC
MtPrx04    159 PAPFHNITQLRQIFANKKLTLHDLVVLSGAHTIGVGHCNLFSNRLF----NFTGKGD---QD-PSLNPTYANFLKT--KC
MtPrx05    185 PAPFSNFTTLQTLFANQGLDMKDLVLLSGAHTIGISLCTSFSNRLY----NFTGKGD---QD-PSLDSEYAKNLKTF-KC
MtPrx06    159 PAPGDSVDEQKQKFATKGLNTQDLVTLVGGHTIGTTACQFFSNRLR----NFTTNGA---AD-PSIDPSFLSQLQT--LC
MtPrx07    163 PPPTFNVNQLTQLFAKKGLTQDEMVTLSGAHTIGRSHCSAFSKRLY----NFSSTSI---QD-PSLDPSYAALLKR--QC
MtPrx08    158 PAPTFNISQLQQSFSQRALSVEDLVALSGGHTLGFSHCSSFQNRIQ----NFNATHD---VD-PSLHQSFAAKLKS--IC
MtPrx09    163 PSPFDPLDTIVSKFTNVGLNLTDVVSLSGAHTIGRARCALFSNRLF----NFSGTGS---PD-STLETGMLTDLQN--LC
MtPrx10    166 PAPFNSLDQLKSAFAAQGLSTTDLVALSGAHTFGRARCTFITDRLY----NFSSTGK---PD-PTLNTTYLQELRK--IC
MtPrx100   182 PMGHENITSLIEYFQSKGLNVLDLVVLSGAHTIGRTSCGSIQYRLY----NYKGTGK---PD-PSLDTKYLNFLQR--KC
MtPrx107   158 VDTSFTMDEMLKLFSSKGLSLLDLVVLSGAHTIGTAHCNTFRGRFQ----Q-DRNGSLRLID-QTIDTNYADQLIK--QC
MtPrx11    166 PAPFNTTDQLKAAFAAQGLDTTDLVALSGAHTFGRAHCSLFVSRLY----NFNGTGS---PD-PTLNTTYLQQLRT--IC
MtPrx110   162 PLFTDDLTTLISHFSKKNLSPKEMVTLSGAHTIGQAQCFTFRGRIY----N----------NASDIDAGFASTRQR--GC
MtPrx111   161 PAPFFTLDQLKAAFLVQGLNTTDLVTLSGAHTFGRAKCSTFINRLY----NFNSTGN---PD-QTLNTTYLQTLRE--IC
MtPrx114   158 VDTSFTMDEMLKLFSSKGLSLLDLVVLSGAHTIGTAHCNTFRGRFQ----Q-DRNGSLRLID-QTIDTNYADQLIK--QC
MtPrx116   149 PPPTFPVKVLSQFFMTKGITTEEMVALLGAXTIGVAHCGFFSNRLS----SSGGK-----PD-PTMDPTLDAKLVK--LC
MtPrx12    177 PAPFNSLDQLKAAFASQGLSTTDLVALSGAHTFGRAHCSLFVSRLY----NFSNTGS---PD-PTLNATYLQQLRN--IC
MtPrx13    162 PAPTLNVGQLIQSFAKRGLGVKDMVTLSGGHTLGFSHCSSFEARLH----NFSSVHD---TD-PRLNTEFALDLKN--KC
MtPrx14    161 PGPHFNLNQLNNMFNLHGLSQTDMVALSGAHTIGFSHCNRFSNRIY----GFSPRSR---ID-PSLNLQYAFQLRQ--MC
MtPrx15    166 PSPAFNLTQLKSNFDNQGLDATDLVALSGAHTIGRGQCRFFVDRLY----NFSNTGN---PD-PTLNTTYLQTLRT--IC
MtPrx16    166 PGPSSSLDQLKSSFAAQGLNTVDLVALSGAHTLGRARCLFILDRLY----DFDNTGK---PD-PTLDPTYLKQLQK--QC
MtPrx17    166 PGPNFSLDRLKSAFAAQGLNTVDLVALSGAHTFGRARCLFILDRLY----NFNNTGK---PD-PTLDTTYLQQLRN--QC
MtPrx18    173 PQGHENITGLIGFFQERGLDMLDLVTLSGSHTIGRSTCYSVMNRIY----NFNGTGK---PD-PSLNIYYLKMLRK--RC
MtPrx19    173 PSPSFNASELITQFGQHGFSAQEMVALSGAHTLGVARCSSFKNRLS----Q---------VD-PALDTEFARTLSR--TC
MtPrx20    165 PAPFNTLVQLKAAFTAQGLNTTDLVALSGAHTFGRAHCAQFVGRLY----NFSSTGS---PD-PTLNTTYLQQLRT--IC
MtPrx21    157 PDVNDSIQQLKTKFLNKGLTEKDLVLLSAAHTIGTTACFFMRKRLY----EFFPFG----SD-PTINLNFLPELKA--RC
MtPrx22    168 PAPTSSLNQLISRFNALGLSTKDLVALSGGHTIGQARCTTFRAHIY----N----------D-SNIDTSFARTRQS--GC
MtPrx23    207 PNPTESLANVTAKFAAVGLNTSDLVALSGAHTFGRGQCRFFNQRLF----NFSGTGK---PD-PTLNSTYLATLQQ--NC
MtPrx24    183 PSPFLNLSGLIAAFKKKGFSADEMVALSGAHTIGKAKCAVFKNRIY----N----------E-SNINPYYRRSLQN--TC
MtPrx25    167 PAPNNTFQTILTKFKLQGLDIVDLVALSGSHTIGKSRCTSFRQRLY----NQTGNGK---QD-FTLDQYYAAELRT--QC
MtPrx26    166 PAPTFNLTQLKSSFDNQNLTTTDLVALSGGHTIGRGQCRFFVDRLY----NFSNTGN---PD-STLNTTYLQTLQA--IC
MtPrx27    164 PSPFDSLDTIISKFDNVGLSVKDVVTLSGAHTIGRARCTFFSNRLF----NFSGTQE---PD-NSLEYEMLTELQN--LC
MtPrx28    162 PGPSSDLSTLTTMFRNKGLTLNDLTVLSGAHTIGQAECQFFRTRIY----N----------E-TNIDTNFATLRKS--NC
MtPrx29    168 PPPFSTLNNLINRFKAQGLSTKDMVALSGAHTIGKARCTVYRDRIY----N----------D-TNIDSLFAKSRQR--NC
MtPrx30    162 PGPFSTLDQLKASFLVQGLNTTDLVTLSGAHTFGRARCSAFINRLY----NFSGTGN---PD-PTLNTTYLQTLRL--IC
```

```
MtPrx32   183 PSPFLDLDGLIAAFKKKGFTAEEMVALSGAHTIGKAKCGLIRNRIY----N----------E-SNIDPNYAKSLQAFLPC
MtPrx33   161 PGPGSDLSGLINAFNNKGFTPKEMVALSGSHTIGEASCRFFRTRIY----N----------E-NNIDSSFANSLQS--SC
MtPrx34   163 PAPFLNLSALKTNFANQGLSAKDLVALSGAHTIGLARCVQFRAHIY----N----------D-SNVDSLFRKSLQN--KC
MtPrx35   167 PGPRFLLDELKTSFLNQGLDTTDLVALSGAHTIGRGVCLLFNDRMY----NFNNTGV---PD-PTLNTTLLQSLQA--IC
MtPrx36   157 PGPGSDLSGLITSFDNKGFTPKEMVALSGSHTIGQASCRFFRTRIY----N----------D-DNIDSSFATSLQA--NC
MtPrx37   163 PAPFFNLSTLKTNFANQGLSVEDLVALSGAHTIGLARCVQFRAHIY----N----------D-SNVDPLFRKSLQN--KC
MtPrx38   167 PQPSFNLNQLNTLFANNGLTQTDMIALSGAHTLGFSHCDRFSNRI---------QTP---VD-PTLNKQYAAQLQQ--MC
MtPrx39   163 PAPFLNLDGLITAFAKKGFTAEEMVTLSGAHTIGLVRCRFFRARIY----N----------E-TNIDPAFAAKMQA--EC
MtPrx40   170 PTPDESLVSIIPKFLYQGLSVTDMVSLVGSHTIGMARCQNFRSRIYG---DYESTSV----K-NPISDNQFNNLKS--IC
MtPrx41   162 PKPTFNLNQLNTIFSKHGLSEKDMIALSGAHTVGFSHCDQFTNRLY--------SSQ---VD-PTLDPTYAQQLMS--GC
MtPrx42   167 PEPGFNLNQLNTLFKHHGLTQTEMIALSGAHTVGFSHCNKFTNRVY----NFKTTSR---VD-PTLDLKYAAQLKS--MC
MtPrx43   162 PSPASDLSTLTKMFQNKGLTLRDLTVLSGAHTIGQAECQFFRNRIY----N----------E-TNIDTNFATLRKA--NC
MtPrx44   163 PGPSSDLSTLTRMFQNKSLTLNDLTVLSGAHTIGQTECQFFRNRIH----N----------E-ANIDRNLATLRKR--NC
MtPrx45   183 PPPNATIEGLLTFFKRQGLDEVDLVALSGAHTIGVAKCATFKQRLY----NQNGNNQ---PD-SNLEKTFYFGLKS--MC
MtPrx46   167 PAPNNTFQTILTKFKLKGLNIVDLVALSGSHTIGDSRCTSFRQRLY----NQTGNGK---SD-FTLDQNYAAQLRT--RC
MtPrx47   168 PDHNESISAVLDKFGAMGIDTPGVVALLGAHSVGRTHCTKLVHRLY------PEV------D-PALNPEHIPHMLK--KC
MtPrx48   176 PQPFNKTDVTLKVFAAQNFDVTDVVALSGAHTFGRAHCGTFFNRLS--P-----------AD-PTLDKTLAQNLKN--TC
MtPrx49   166 PGPAFSLSQLIKNFDNQGLNEKDLVALSGAHTIGFSRCFLFRDRIY----K----------D-NNINAYFAKQLQN--VC
MtPrx50   169 PSPFLNADQLIANFAQKGLSIDEMVTLSGAHSIGVSHCSSFSNRLY----SFSDTIS---QD-PSMDPSFAESLKT--KC
MtPrx52   163 PSPFEPLENITAKFISKGLEKKDVAVLSGAHTFGFAQCFTFKPRLF----DFGGSGK---SD-PSLDSSLLQNLQK--VC
MtPrx53   163 PAPFHNLTVLLTLFGNVGLDANDLVLLSGAHTIGVSHCSTISTRLY----NFTGKGD---QD-PDLDNEYAKNLKTF-KC
MtPrx54   165 PRPTSNLNILTSMFKNVGLSTKDLVALSGAHTIGQARCTTFRVRIY----N----------E-TNIDTSFASTRQS--NC
MtPrx55   159 PAPTFNISQLQQSFSQRGLSLQDLVALSGGHTLGFAHCSSFQNRIH----KFSPKQA---VD-PSINPSFASNLQS--KC
MtPrx56   163 PNHNDSISSVLSSFQAIGIDVEATVALLGAHSVGRVHCMNLVHRLY------PTV------D-PTLDPTHAAYLKR--RC
MtPrx57   162 PSPFSDLSTLTTMFSAKGLTASDLTVLSGAHTIGQGECQFFRNRIY----N----------E-TNIDTNFATLRKS--NC
MtPrx58   163 PSPFEPLENITAKFISKGLEKKDVAVLSGAHTFGFAQCFTFKPRLF----DFGGSGK---SD-PSLDSSLLQNLQR--VC
MtPrx60   168 PSPSISWQESLAYFKSKGLNVLDMTTLLGAHTLGQTHCSYIVDRLY----NYNGTGN---SD-PSMDATFRDTLRG--LC
MtPrx61   168 PAPSLNLDGLIATFARKNFTALEMVTLSGAHTIGDARCTSFRGRIY----N----------E-TNIDPSFAESKRL--LC
MtPrx63   162 PRPSFNLTRLTTMFLAKGLTASDLTVLSGAHTIGQGECRLFRTRIY----N----------E-TNIDTNFATLRKS--NC
MtPrx64   213 PSPYADLTQTRASFKSRGFDEREMVTLLGAHSIGVIPCKFFENCLY----NFSGTNE---PD-PSLDTQFLNVLRS--KC
MtPrx65   169 PPPTFSAEQLIDNFDRKGLSVDEMVTLSGAHSIGVSHCSSFSKRLY----SFNLTFP---QD-PSMDPNFARLLKS--KC
MtPrx66   153 PSPLEPVSVHRQKFAAKGLNDHDLVTLLGAHTIGQTDCRFFSYRLY----NFTTTGN---AD-PTINQAFLAQLKA--IC
MtPrx67   161 PKFTDDLTTLIAHFTNKGLTLKDMVTLSGAHTIGQAQCFTFRDRIY----N----------NASDIDAGFASTRRR--GC
MtPrx70   165 PPPFFNFSQLITNFKSHGLNLKDLVVLSGGHTIGFSKCTNFRNRIY----N----------D-TNIDTNFAANLQK--TC
MtPrx71   166 PAPFFNLSQLKSSFAAQGLNTVDLVALSGAHTFGRARCSLFVDRLY----NFSNTGE---PD-PTLDTTYLKQLQN--EC
MtPrx74   182 PRANSTVDELIKIFNSKGLTIQDMVALSGAHTIGFAHCKNFLTRLY----NYRGKGQ---PD-PDMNPKLLKALRM--YC
```

```
MtPrx75    173 PVPFARTDELLTVFGSKKFDATDVVALSGAHTFGQAHCPTMFNRVI--D-----------SD-PPIEPNFKKQLEA--TC
MtPrx76    161 PLFTDDLTTLISHFNKKNLTPRDMVTLSGAHTIGQAQCFTFRGRIY----N----------NASDIDAGFANTRQR--GC
MtPrx78    155 PGPFSNVSQALNFFTSKGLTLEDMVTLMGAHTIGFAKCIFFQSRLS----SFNGN-----ID-PTMDPNLDAFLVE--KC
MtPrx79    173 PKPSMTMTQIVSIFTKRGFTVEEMVALSGAHTVGFSHCSEISSDIY----N--NSSG---SG-SGYNPRFVEGLKK--AC
MtPrx81    166 PGPAFSLSQLIKNFDNKGLNEKDLVALSGAHTIGFSRCLLFRDRIY----N----------D-KNIDANFAKQLQD--IC
MtPrx82    195 PRPDDNITRTLHLFNLRGFKARETVSLLGEHNIGKIGCDFIQQRLY----DFQGTGQ---PD-PSIPLDFLSQMRQ--NC
MtPrx83    212 PMPNWPMEKMVDLFTKKGFTIEEMVILLGAHSIGVAHCDVFMERIY----NYADTRK---PD-PLLPFPIVNELQQ--IC
MtPrx84    165 PAPTSNLNTLTSMFSAVGLSSKDLVTLSGAHTIGQARCTNFRARIY----N----------E-TNINAAFASTRQS--NC
MtPrx85    175 PGPNSTVDMLVAKFENVGLTLQDMVALSGAHTIGKARCSTFSSRLR------SNSVS---DG-PYVNAEFVSSLKR--LC
MtPrx86    165 PPAFFNFSQLIANFQSQGLNLKDLVVLSGGHTIGFSRCTNFRSRIF----N----------D-TNINTNFAANLQK--TC
MtPrx87    169 PPPTFSAEQLIDNFDRKGLSVDEMVTLSGAHSIGVSHCSSFSKRLY----SFNATFP---QD-PSMDPDFARLLRS--KC
MtPrx88    156 PGPESSISQTLQAFKSKGMTLEEMVTLLGAHTVGFAHCSFIGKRL--------GS-----ND-SSMDPNLRKRLVQ--WC
MtPrx89    154 PGPNIPIGALSQFFAAKGITTEEMVTLLGAHTVGVAHCGFFASRLS----SVRGK-----PD-PTMDPALDTKLVK--LC
MtPrx90    163 PAPNSSLETLINNFKQQGLDIEDLVVLSGSHTIGRARCLSFRQRIYETKQEYHHAYD----R-YKRYTTFRRILQS--IC
MtPrx91    161 PLPTMTNSQVIDIFTSKGFTVQEMVALAGAHTIGFSHCKQFSNRLF----NFSKTTE---TD-PKYNPEYAAGLKK--LC
MtPrx92    161 PSPSFNISQLQKSFSQRGLSMEDLVALSGGHTLGFSHCSSFRNRIH----NFDATHD---VD-PSLNPSFASKLKS--IC
MtPrx93    166 PSPTVTVDEFLSIFKSKGMNIQESVAILGAHTLGVGHCLSIVGRLY--N-QNQQI------G-NNMNLGYETSLRL--AC
MtPrx94    177 PSPRANASTLIDLFQRFNLTVKDLVALSGSHSIGQGRCFSIMFRLY----NQSGSGK---PD-PALDPAFRLELDK--LC
MtPrx95    165 PSPRTNATALITLFQKYNLSVKDLVALSGSHSIGKARCFSIMFRLY----NQSGSGK---PD-PAIDHVFRAELDK--LC
MtPrx97    162 PSPFSDLSTLTKMFTDKGLTASDLTVLSGAHTIGQGECQFFRNRIY----N----------E-TNIDTNFATLRKL--NC
MtPrx98    165 PAPTSNLNTLTSMFSAVGLSSKDLVTLSGAHTIGQARCTTFRARIY----N----------E-TNIDTSFASTRQS-NC
consensus  401 p p   l  li  F  hglt  dlvalsgahtiG arC  f  rly    n          d psid  fa  l     C
```

```
MtPrx01     232 -PR-N---G---G--------DNN---LTPL------------------------------DF-T--P-TRVENTYYRDLL
MtPrx02     228 -PK-I---G---G--------DDN---LAPF------------------------------DS-T--P-NKVDTSYYKALL
MtPrx03     230 -PL-H---G---G--------DDN---FEAL------------------------------DFLT--P-QVFDNSYYKNLV
MtPrx04     229 -QG-L---S---D--------TTT---TVEM------------------------------DPNS--S-TTFDNDYYPVLL
MtPrx05     256 -KN-I---N---D--------NTT---IVEL------------------------------DPGS--R-NTFDLGYYSQVV
MtPrx06     229 -PQ-----N---S--------GAT-N-RIAL------------------------------DTGS--Q-NKFDNSYYANLR
MtPrx07     233 -PQ-G---N---T--------NQN-L-VVPM------------------------------DPSS--P-GTADVGYYNDIL
MtPrx08     228 -PL-K---N---K--------AKN---AGTT------------------------------MDPS--A-TNFDNTYYKLIL
MtPrx09     233 -PQ-T---G---D--------GNT---TAVL------------------------------DRNS--T-DLFDNHYYKNLL
MtPrx10     236 -PN-G---G---P--------PNN---LANF------------------------------DPTT--P-DKFDKNYYSNLQ
MtPrx100    252 -RW-------------------AS-E-YVDL------------------------------DATT--P-KKFDRMYYINLE
MtPrx107    230 -PI-----N---A--------QPS-V-AVNI------------------------------DPET--S-MLFDNQYYRNLL
MtPrx11     236 -PN-G---G---P--------GTN---LTNF------------------------------DPTT--P-DKFDKNYYSNLQ
MtPrx110    226 -PS-S---STTSN--------DQK---LAAL------------------------------DLVT--P-NSFDNNYFKNLI
MtPrx111    231 -PQ-N---G---T--------GNN---LTNL------------------------------DLTT--P-NQFDNKFYSNLQ
MtPrx114    230 -PI-----N---A--------QPS-V-AVNI------------------------------DPET--S-MLFDNQYYRNLL
MtPrx116    217 -K------S---N--------VDG---ATFL------------------------------DQNT--S-FVIDQEIYKQIL
MtPrx12     247 -PN-G---G---P--------GTP---LASF------------------------------DPTT--P-DKFDKNYYSNLQ
MtPrx13     232 -PK-P---N---N--------NQN---AGQF------------------------------LDST--A-SVFDNDYYKQLL
MtPrx14     231 -PI-----R---V--------DPR-I-AINM------------------------------DPVS--P-QKFDNQYFKNLQ
MtPrx15     236 -PN-G---G---P--------GST---LTDL------------------------------DPAT--P-DTFDSAYYSNLR
MtPrx16     236 -PQ-N---G---P--------GNN---VVNF------------------------------DPTT--P-DKFDKNYYNNLQ
MtPrx17     236 -PQ-N---G---T--------GNN---RVNF------------------------------DPTT--P-DTLDKNFYNNLQ
MtPrx18     243 -KK-------------------DL-D-LVHL------------------------------DVIT--P-RTFDTTYYTNLK
MtPrx19     237 --------T---S--------GDN---AEQP------------------------------FDAT--R-NDFDNVYFNALL
MtPrx20     235 -PN-G---G---P--------GTN---LTNF------------------------------DPTT--P-DKFDKNYYSNLQ
MtPrx21     226 -PK-----D---G--------DVN-I-RLAM------------------------------DEGS--D-LKFDKSILKNIR
MtPrx22     231 -PK-T---SGS-G--------DNN---LAPL------------------------------DLAT--P-TSFDNHYFKNLV
MtPrx23     277 -PQ-N---G---S--------GNT---LNNL------------------------------DPSS--P-NNFDNNYFKNLL
MtPrx24     246 -PR-N---G---G--------DNN---LANL------------------------------DSTT--P-AFFDSAYYRNLL
MtPrx25     237 -PR-S---G---G--------DQN---LFFL------------------------------DYVT--P-TKFDNNYFKNLL
MtPrx26     236 -PN-G---G---P--------GTN---LTDL------------------------------DPTT--P-DTFDSNYYSNLQ
MtPrx27     234 -PQ-D---G---D--------GNT---TTVL------------------------------DPYS--F-DQFDNNYFKNLL
MtPrx28     225 -PT-S---G---G--------DIN---LAPL------------------------------DSVS--P-VTFDNNYYNDLV
MtPrx29     231 -PR-K---SGTIK--------DNN---VAVL------------------------------DFKT--P-NHFDNLYYKNLI
MtPrx30     232 -PQ-N---S---T--------GNN---LANL------------------------------DLTT--P-NHFDNKYYSNLQ
```

```
MtPrx32   248 -PK-S---G---G--------DNN---LASL-----------------------------DATT--P-NFFDNAYYRNLL
MtPrx33   224 -PR-T---G---G--------DLN---LSPL-----------------------------DTTS--P-NTFDNAYFKNLQ
MtPrx34   226 -PR-S---G---N--------DNV---LEPL-----------------------------DHQT--P-THFDNLYFKNLL
MtPrx35   237 -PDIG---V---L--------GTN---LTNL-----------------------------DVST--P-DTFDSNYYSNLQ
MtPrx36   220 -PT-T---G---G--------DDN---LSPL-----------------------------DTTT--P-NTFDNSYFQNLQ
MtPrx37   226 -PR-S---G---N--------DNV---LEPF-----------------------------DYQT--P-THFDNLYFKNLL
MtPrx38   232 -PR-----N---V--------DPR-I-AINM-----------------------------DPTT--P-RTFDNVYYKNLQ
MtPrx39   226 -PF-E---G---G--------DDN---FSPF-----------------------------DSSKPEA-HDFDNGYYQNLV
MtPrx40   240 PPI-G---G---G--------DNN---ITAM-----------------------------DYVT--P-NLFDNSFYQLLL
MtPrx41   228 -PR-----N---V--------DPN-I-VLAL-----------------------------DTQT--E-HTFDNLYYKNLV
MtPrx42   237 -PR-----N---V--------DPR-V-AVDM-----------------------------DPVT--P-HAFDNVYFKNLQ
MtPrx43   225 -PL-S---G---G--------DTN---LAPL-----------------------------DSVS--P-VTFDNNYYRDLV
MtPrx44   226 -PT-S---G---G--------DTN---LAPF-----------------------------DSVT--P-TKFDNNYYKDLI
MtPrx45   253 -PR-S---G---G--------DNI---ISPL-----------------------------DFGS--P-RMFDNTYYKLLL
MtPrx46   237 -PR-S---G---G--------DQN---LFVL-----------------------------DFVT--P-VKFDNNYYKNLL
MtPrx47   233 -PD-S---I---P--------DPKAVQYVRN-----------------------------DRGT--P-MILDNNYYRNIL
MtPrx48   240 -PN-----A---N--------SGN---TANL-----------------------------DIRT--P-ATFDNKYYLDLM
MtPrx49   229 -PR-E---G---G--------DSN---LAPL-----------------------------DSVT--S-AKFDVAYYSQLI
MtPrx50   239 -PP-P---P---S--------NTN-P-IVML-----------------------------DVAT--P-NRLDNLYYEGLI
MtPrx52   233 -PNQA---D---S--------DSN---LAPL-----------------------------DPVT--T-NTFDNTYYKNVL
MtPrx53   234 -KN-I---N---D--------QTT---LIEM-----------------------------DPGS--R-NTFDLGYFKQVV
MtPrx54   228 -PK-T---SGS-G--------DNN---LAPL-----------------------------DLHT--P-TSFDNCYYRNLV
MtPrx55   229 -HI-K---N---K--------VKN---SGSP-----------------------------LDST--A-TYFDNAYYKLLL
MtPrx56   228 -PT-P---N---P--------DPKAVQYVRN-----------------------------DLKT--P-MIIDNNYYKNIL
MtPrx57   225 -PL-S---G---G--------DTN---LAPL-----------------------------DTLT--P-TSFDNNYYKNLV
MtPrx58   233 -PNQA---D---S--------DTN---LAPL-----------------------------DPVT--S-NTFDNTYYRNVL
MtPrx60   238 -PPRTKKGQ---S--------DPL-V-YLNP-----------------------------DSGK--N-YIFRESYYKRIL
MtPrx61   231 -PF-N---G---G--------DNN---ISTL-----------------------------SNS---S-INFDNTYYNDLV
MtPrx63   225 -SF-S---S--DN--------DTN---LAPL-----------------------------DTLT--P-TSFDNNYYKNLV
MtPrx64   283 -NE-T---D---A--------LST---SASA----------YSSHASPSSLVEEQQEITTDSGE--SLSNFGTLYYRRLL
MtPrx65   239 -PP-P---Q---S-------QSIN-P-TVVL-----------------------------DGST--P-NDLDNMYYKRLK
MtPrx66   223 -PK-----N---G--------DGL-R-RVAL-----------------------------DKDS--P-AKFDVSFFKNVR
MtPrx67   225 -PS-L---SSTTN--------NQK---LAAL-----------------------------DLVT--P-NSFDNNYFKNLI
MtPrx70   228 -PK-I---G---G--------DDN---LAPF-----------------------------DS-T--P-SRVDTKYYKALL
MtPrx71   236 -PQ-N---G---P--------GNN---RVNF-----------------------------DPTT--P-DTLDKNFYNNLQ
MtPrx74   252 -PN-F---G---G--------NTD-I-VAPF-----------------------------DATT--P-FIFDHAYYGNLQ
```

```
MtPrx75     237 -PN-----E---E--------SLN---AVNL-----------------------------DVRT--P-NTFDNMYYINLL
MtPrx76     225 -PS-S---RTTSN--------DQK---LAAL-----------------------------DLVT--P-NSFDNNYFKNLI
MtPrx78     223 -GS-----R---G--------NET---SVFL-----------------------------DQKT--P-FDFDNEFYNQIV
MtPrx79     241 -GD-Y---K---K--------NPT-L-SVFN-----------------------------DIMT--P-NKFDNVYFQNLP
MtPrx81     229 -PR-E---G---G--------DSN---LAAL-----------------------------DCVT--P-AKFDEAYYMELI
MtPrx82     265 -PD-N---N---K--------NNV---SSNGMFSTFTVSKPMNVHHSNNKGMSYKQALSSAVSS--G-ASFDTHYYQRLL
MtPrx83     282 -AN-P---G---T--PLF---RNP---VVNF-----------------------------DET---P-ALLDNLFFKNMV
MtPrx84     228 -PK-A---SGS-G--------DNN---LAPL-----------------------------DLQT--P-SSFDNNYFKNLV
MtPrx85     243 -SG-Q---D---N--------SNR---IAHL-----------------------------DLVT--P-ATFDNQYYINLL
MtPrx86     228 -PR-I---G---G--------DDN---LAPF-----------------------------DS-T--P-SRVDTKYYKALL
MtPrx87     239 -PP-P---Q---SQQSQSQIQNLD-S-TVAF-----------------------------DGST--P-NDLDNMYYKRLK
MtPrx88     220 -GV-----E---G--------KDP---LVFL-----------------------------DQNT--S-FVFDHQFYNQIL
MtPrx89     222 -K------S---N--------SDG---AAFL-----------------------------DQNT--S-FTVDNEFYKQIL
MtPrx90     236 -PV-T---G---R--------DDK---FAPL-----------------------------DFQT--P-KRFDNQYFINII
MtPrx91     231 -QN-Y---Q---K--------DTS-M-SAFN-----------------------------DVMT--P-SKFDNMYFKNLK
MtPrx92     231 -PI-I---N---Q--------VKN---AGTT-----------------------------LDAS--S-TTFDNTYYKLIL
MtPrx93     234 -PT-V---I---P--------MTN-LTFVPN-----------------------------DM-T--P-TIFDNQYYRDIM
MtPrx94     247 -PL-D---V---D--------QNK---TGNL-----------------------------DST---P-VIFDNQYFKDLV
MtPrx95     235 -PR-D---V---D--------QNK---TGNL-----------------------------DAT---P-VIFDNQYFKDLV
MtPrx97     225 -PL-S---G---G--------DTN---LAPL-----------------------------DTLT--P-TNFDNNYYKNLV
MtPrx98     228 -PN-T---SGS-G--------DNN---LAPL-----------------------------DLQT--P-TSFDNNYFKNLV
consensus   481  p                     n   l  l                             d  t  p   fdn yyrnll
```

```
MtPrx01     260 -YKRGVLHSDQQLF-K---GQ---GSESDKLVQLYSKNT-F-----AFASDFKTSLIKMGN-I-KPLTG-RQ-GEIRLNC
MtPrx02     256 -YKRGLLHSDQELF-K---GD---GSQSDRLVQLYSKNS-Y-----AFAYDFGVSMIKMGN-I-KPLTG-KK-GEIRQNC
MtPrx03     259 -SKIGLFHSDQELF-K---GD---GSESDMLVELYSRDS-D-----AFARDFKASMIKMGN-I-KPLTG-NF-GEIRTYC
MtPrx04     258 -QNKGLFTSDAALL-T---TK-Q----SRNIVNELVSQN-------KFFTEFSQSMKRMGA-I-EVLTG-SN-GEIRRKC
MtPrx05     285 -KRRGLFESDSALL-T---NS-V----TKALVTQFLQGSLE-----NFYAEFAKSIEKMGQ-I-KVKTG-SQ-GVIRKHC
MtPrx06     258 -NGRGILQSDQALW-N---DA-S----TKTFVQRYLG-L-RGLLGLTFNVEFGNSMVKMSN-I-GVKTG-VD-GEIRKIC
MtPrx07     263 -ANRGLFTSDQTLL-T---NT-G----TARKVHQNARNP-Y-----LWSNKFADAMVKMGQ-V-GVLTG-NA-GEIRTNC
MtPrx08     257 -QQKGLFSSDQALL-D---SP-K----TKQLVSKFAASQ-K-----AFFDAFAKSMIKMSS-I-N---G-GQ--EVRKDC
MtPrx09     262 -NGKGLLSSDQILI-S---TD-EANSTSKPLVQSYNDNA-T-----LFFGDFVKSMIKMGN-I-NPKTG-SD-GEIRKSC
MtPrx10     265 -GKKGLLQSDQELF-S---TS---GADTISIVNKFSADK-N-----AFFDSFEAAMIKMGN-I-GVLTG-KK-GEIRKHC
MtPrx100    278 -KKMGLLTTDQLLY-S---DP-R----TSQLVSALTT------SS-VFEHQFAVSMSKFGV-I-DVLTGDDE-GEIRTNC
MtPrx107    259 -DRKVLFQSDSVLM-N---ND-D----TRKLVEDFANDQ-E-----LFFDNWGVSFVKLTS-I-GVKTD-EE-GEIRRSC
MtPrx11     265 -VKKGLLQSDQELF-S---TS---GSDTISIVNKFATDQ-K-----AFFESFKAAMIKMGN-I-GVLTG-KQ-GEIRKQC
MtPrx110    258 -QKKGLLQSDQVLF-S-G-GS------TNSFVSEYSNNP-T-----TFKSDFATAMIKMGD-I-EPLTG-SA-GVIRSIC
MtPrx111    260 -SHKGLLQSDQELF-S---TP---NADTIAIVNSFSSNQ-A-----LFFENFRVSMIKMAN-I-SVLTG-NE-GEIRLQC
MtPrx114    259 -DRKVLFQSDSVLM-N---ND-D----TRKLVEDFANDQ-E-----LFFDNWGVSFVKLTS-I-GVKTD-EE-GEIRRSC
MtPrx116    244 -LKRGILQIDQKLT-L---DK-S----TSLFVSNFASNG-E-----KFVNNFATAMIKMGK-I-GLLIG-NE-GEVRKNC
MtPrx12     276 -VKKGLLQSDQELF-S---TS---GADTISIVNNFATDQ-K-----AFFESFKAAMIKMGN-I-GVLTG-NQ-GEIRKQC
MtPrx13     261 -AGKGVFSSDQSLV-G---DY-R----TRWIVEAFARDQ-S-----LFFKEFAASMLKLGN-I-R---G-SDNGEVRLNC
MtPrx14     260 -QGKGLFTSDQVLF-T---DS-R----SKATVNLFASNP-K-----AFESAFINAITKLGR-V-GVKTG-NQ-GEIRFDC
MtPrx15     265 -IQKGLFQSDQVLS-S---TS---GADTIAIVNSFNNNQ-T-----LFFEAFKASMIKMSR-I-KVLTG-SQ-GEIRKQC
MtPrx16     265 -GKKGLLQSDQELF-S---TP---GADTISIVNNFGNNQ-N-----VFFQNFINSMIKMGN-I-GVLTG-KK-GEIRKQC
MtPrx17     265 -GKKGLLQSDQELF-S---TP---GADTISIVNSFANSQ-N-----VFFQNFINSMIKMGN-I-DVLTG-KK-GEIRKQC
MtPrx18     269 -RKAGLLSTDQLLF-S---DK-R----TSPFVDLFAT------QPFVFTSQFAVSMVKLGN-V-QVLTRPNE-GEIRVNC
MtPrx19     263 -RKNGVLFSDQTLY-S---SP-R----TRNIVNAYAMNQ-A-----MFFLDFQQAMVKMGL-I-DIKQG-SN-GEVRSNC
MtPrx20     264 -VKKGLLQSDQELF-S---TS---GADTISIVNKFSTDQ-N-----AFFESFKAAMIKMGN-I-GVLTG-TK-GEIRKQC
MtPrx21     255 -EGFAVLASDARLN-D---DF-V----TKSVIDSYFNPI-NPTFGPSFENDFVQSMVKMGQ-I-GVKTG-SV-GNIRRVC
MtPrx22     262 -DSKGLLHSDQQLF-N-G-GS------TDSIVHEYSLYP-S-----SFSSDFVTAMIKMGD-I-SPLTG-SN-GEIRKQC
MtPrx23     306 -KNQGLLQTDQELF-S---TN---GAATISIVNNFASNQ-T-----AFFEAFVQSMINMGN-I-SPLIG-SQ-GEIRSDC
MtPrx24     275 -FKRGLLHSDQELY-N-G-GS------TDYKVLAYARNP-Y-----LFRFDFAKAMIKMGN-I-SPLTG-NQ-GQIRKYC
MtPrx25     266 -AYKGLLSSDEILL-T---KN----QESAELVKLYAERN-D-----LFFEQFAKSMIKMGN-I-SPLTG-SR-GNIRTNC
MtPrx26     265 -VGNGLFQSDQELF-S---TN---GSDTISIVNSFANNQ-T-----LFFENFVASMIKMGN-I-GVLTG-SQ-GEIRTQC
MtPrx27     263 -NGKGLLSSDQILF-S---SDEETTSTTKQLVQYYSENE-R-----IFFMEFAYAMIKMGN-I-NPLIG-SE-GEIRKSC
MtPrx28     254 -ANKGLLHSDQALF-N-GVGS------QVSLVRTYSRNN-I-----AFKRDFAAAMVKMSR-I-SPLTG-TN-GEIRKNC
MtPrx29     263 -NKKGLLHSDQELF-N-G-GS------TDSLVKSYSNNQ-N-----AFESDFAIAMIKMGN-N-KPLTG-SN-GEIRKQC
MtPrx30     261 -NLNGLLHSDQVLL-S---TP---NADTIAIVNSFSNNQ-S-----LFFLNFRVSMIKMAN-I-GVLTG-DE-GEIRLQC
```

```
MtPrx32     277 -NKKGLLHSDQQLY-N-G-GS------TDYKVSAYANNP-L-----LFSIDFANAMIKMGN-I-SPLTG-DQ-GQIRKYC
MtPrx33     253 -NQKGLFHSDQVLF-D-E-VT------TKSQVNSYVRNP-L-----SFKVDFANAMFKMAN-I-GPLTG-SS-GQVRKNC
MtPrx34     255 -AKKALLHSDQELF-N-G-SS------TDNLVRKYATDN-A-----KFFKAFAKGMVKMSS-I-KPLTG-SN-GQIRTNC
MtPrx35     267 -AGNGLFQSDQELF-S---TP---GADTIAIVNSFSSNQ-T-----LFFEAFKASMIKMGN-I-GVLTG-TQ-GEVRTHC
MtPrx36     249 -SQKGLFSSDQALF-N-G-GS------TDSDVDEYSSDS-S-----SFATDFANAMVKMGN-I-NPITG-SN-GQIRTNC
MtPrx37     255 -AKKTLLHSDHELF-NIG-SS------TNNLVRKYATNN-A-----EFFKAFAEGMVKMSSSI-KPLTG-SN-GQIRINC
MtPrx38     261 -QGKGLFTSDQILF-T---DT-R----SRNTVNSFATNG-N-----VFNANFITAMTKLGR-V-GVKNA-RN-GKIRTDC
MtPrx39     257 -KSKGLIHSDQQLFGN-G-TS------TNAQVRRYSRNF-G-----RFKKDFADAMFKMSM-I-SPLTG-TE-GEIRTNC
MtPrx40     270 -KGEGVLNSDQEMY-S---SV-F-GIETRELVKKYAADS-L-----AFFQQFSDSMVKMGN-I-TNSESFIT-GEVRKNC
MtPrx41     257 -NGKGLLSSDQVLF-T---DD-A----SRSTVVEFANDG-S-----KFFEAFVVAIKKLGR-V-GVKTG-KE-GEIRRDC
MtPrx42     266 -KGKGLFTSDQVLF-T---DS-R----SKAAVNAFASSN-K-----IFHANFVAAMTKLGR-V-GVKNS-HN-GNIRTDC
MtPrx43     254 -ANKGLLNSDQALF-N-GVGS------PVSLVRAYSING-F-----AFRRDFAFAMVKMSR-I-SPLTG-TN-GEIRKNC
MtPrx44     255 -ANKGLLHSDQVLF-N-GGGS------QISLVRKYSRDG-A-----AFSRDFAAAMVKMSK-I-SPLTG-TN-GEIRKNC
MtPrx45     282 -RGKGLLNSDEVLL-T---GS-V--KETRDLVKKYEQDE-S-----LFFQQFALSMIKLGN-I-RPLTG-FN-GEVRKNC
MtPrx46     266 -ANKGLLSSDEILL-T---KN----QVSADLVKKYAESN-D-----LFFEQFAKSMVKMGN-I-TPLTG-SR-GEIRKRC
MtPrx47     265 -DNKGLLIVDHQLA-H---DK-R----TKPYVKKMAKSQ-E-----YFFKEFSRAITLLSE-N-NPLTG-TK-GEIRKQC
MtPrx48     268 -NKQGLFTSDQDLN-I---DS-R----TKGLVNDFAVNQ-G-----LFFEKFVNAFIKVSQ-I-NVLVG-NQ-GEIRGKC
MtPrx49     258 -KKKGLLHSDQELL-K---GG-----YTGALVRKYRRDT-R-----TFYKDFAKSMIKMGN-I-KPLTG-KQ-GEVRYNC
MtPrx50     269 -NHRGLLTSDQTLL-S---SQ-S----TQESVLSNANYG-S-----NWATKFAQAMVHMGS-I-DVLSG-YD-GEIRKHC
MtPrx52     263 -SNSGLLQSDQALL-G---DN-----TTSALVTNYSKWP-I-----LFFRDFAVSVEKMGR-I-GILAG-QQ-GQIRKNC
MtPrx53     263 -KRRGLFQSDAALL-K---SS-T----TRSILAQHLQSN-E-----KFFTEFGRSMEKMGR-I-NVKIG-TE-GEIRKHC
MtPrx54     259 -QNKGLLHSDQQLF-N-G-GS------TNSIVSGYFNNQ-N-----SFFSDFATAMIKMGD-I-KPLTG-SN-GEIRKNC
MtPrx55     258 -QGKSILSSDQALL-T---HP-T----TKALVSKYAHSQ-M-----EFERAFVKSMIKMSS-ITN---G-GK--QIRLQC
MtPrx56     260 -QHKGLLTVDEELA-T---DP-R----TSPYVKKMAADN-G-----YFNEQFSRAVQLLSE-N-NPLIG-DQ-GEIRKDC
MtPrx57     254 -ASKGLFHSDQALF-N-N-GS------QDNLVRSYSTNG-A-----TFSRDFAVAMVKLSK-I-SPLTG-TN-GEIRKNC
MtPrx58     263 -SNSGLLQSDQALL-G---DS-----TTASLVNYYSKWP-I-----LFFRDFAVSVEKMGR-I-GVLTG-QQ-GQIRKNC
MtPrx60     272 -RNEAVLGIDQQLL-F---GD-D----TKEITEEFAAGF-E-----DFRRSFAQSMFKMGN-I-KVLTG-NQ-GEIRRSC
MtPrx61     259 -SKKGLLHSDQQLL-N-G-LS------TSNQVIAYTTDN-E-----SFKRDFANVMIKMGM-I-SPLTG-SD-GQIRQNC
MtPrx63     255 -ASKGLFHSDQVLF-N-N-GS------QDNLVRSYSTNE-A-----AFSTDFAAAMVKLSK-I-SPLTG-TN-GEIRKNC
MtPrx64     332 -QGKGILYEDQQLM-E---GE-K----TRYWVQ-YASNR-T-----LFHQDFALAMMKLSD-I-RVLTK-PM-GQIRCSC
MtPrx65     270 -NNRGLLTSDQTLL-N---SG-L----TRRMVLKNARHA-A-----IWNVKFAKAMVHMGS-I-DVLTG-SE-GEIRERC
MtPrx66     252 -DGNGILESDQRLW-E---DS-A----TRRVVENYGGNF-RGLLGLRFDFEFPKAMIKLSS-V-DVKTG-ID-GEIRKVC
MtPrx67     257 -QKKGLLQSDQVLF-G-GGGS------TDSIVSEYSKNP-T-----TFKSDFAAAMIKMGD-I-QPLTG-SA-GIIRSIC
MtPrx70     256 -NKQGLLHSDQELF-K---GD---GSQSDRLVQLYSKNS-Y-----AFAYDFGVSMIKMGN-I-KPLTG-KK-GEIRCNC
MtPrx71     265 -VKKGLLQSDQELF-S---TP---NADTTSIVNNFANNQ-S-----AFFESFKKAMIKMGN-I-GVLTG-KK-GEIRKQC
MtPrx74     282 -NKMGLLASDQALA-L---DP-R----TKSLVQDFAKDK-Q-----KFFQAFASAMDKMSL-V-KVVRG-KKHGERRRDC
```

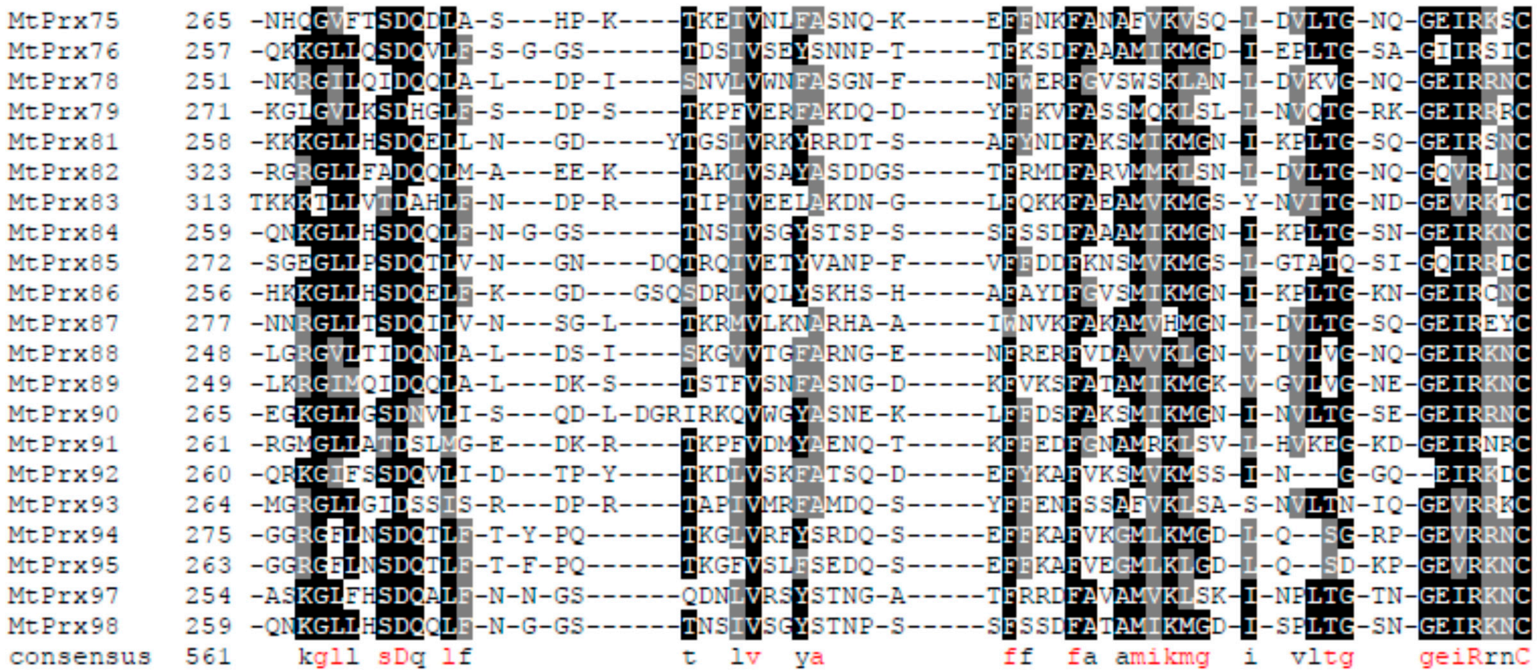

Figure S4. Alignment of *M. truncatula* class III peroxidases (partial sequences) showing in red the conserved residues described in [37]. The alignment was performed with Kalign and the conserved residues were shaded using BoxShade (see Materials and Methods section).

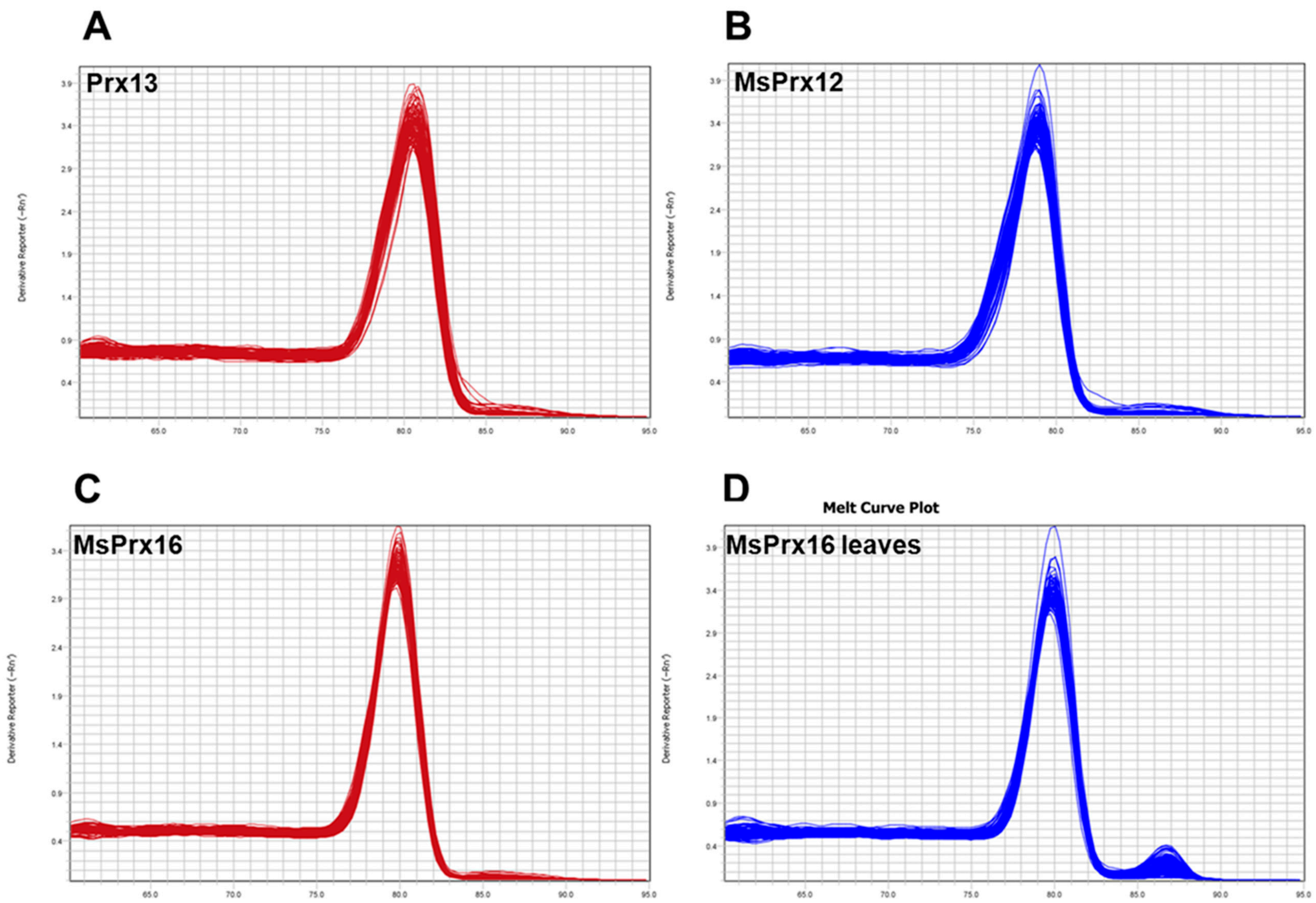

Figure S5. Melt curve analysis of the three class III peroxidases Prx 13 (**A**); MsPrx12 (**B**); MsPrx16 (**C**) in roots and stems of alfalfa; In (**D**) the melt curve analysis of MsPrx16 in alfalfa leaves shows two peaks, therefore the gene was not retained for study in these organs.

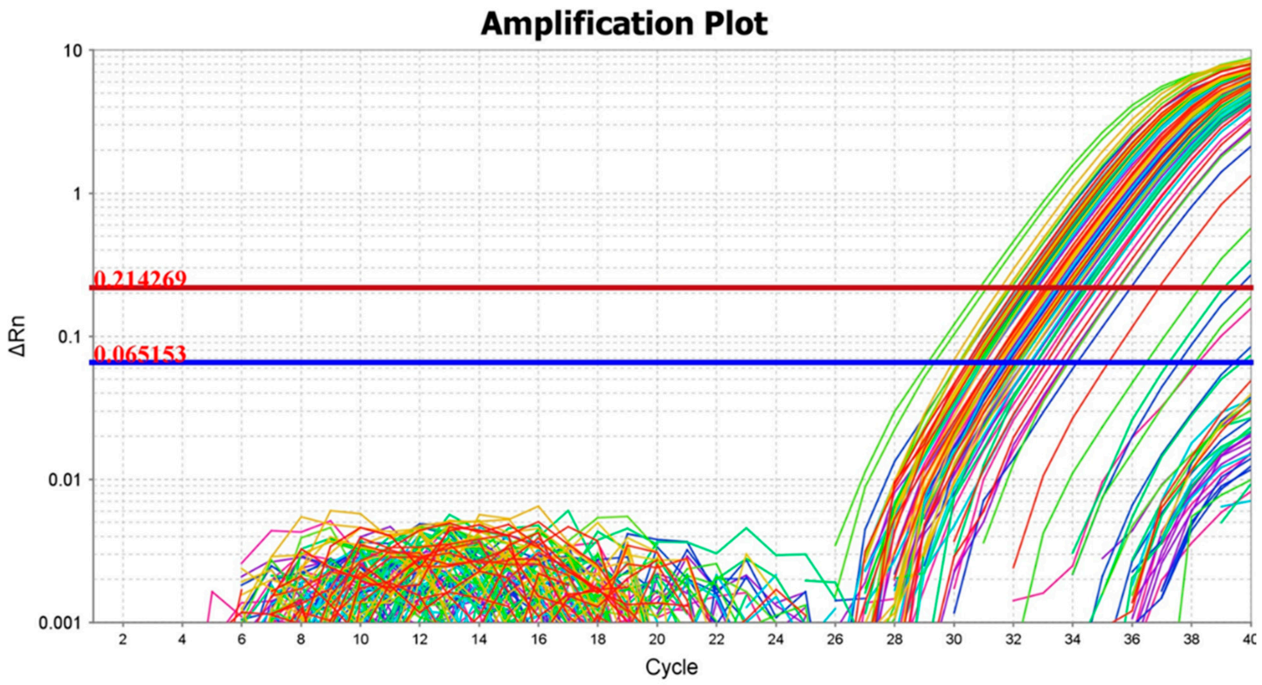

Figure S6. Amplification curves for Medtr4g078885.1 in alfalfa roots.

Table S1. Characteristics of putative dirigent/dirigent-like proteins from *M. truncatula*. Details concerning the gene accession, the protein length, the subfamily, the localization, the signal peptide (SP) length and the *N*-glycosylation sites are provided. The positions corresponding to putative *N*-glycosylation sites followed by a proline, which are very unlikely to be modified, are indicated in italics. "S" is secreted, "other" indicates a localization that is neither chloroplastic, nor mitochondrial, nor apoplastic (as computed by TargetP).

Gene Accession	Length	Subfamily	Location	SP (length)	*N*-Glycosylation
Medtr4g122130.1	180	f	S	28	6-33-174
Medtr4g122110.1	180	f	S	26	6-33-174
Medtr1g056370.1	186	e	S	20	88-169
Medtr7g093850.1	194	b/d	S	28	16
Medtr7g093820.1	194	b/d	S	28	16
Medtr3g034030.1	186	e	S	24	*56*-179
Medtr7g093830.1	193	b/d	S	27	15-132
Medtr7g093870.1	218	b/d	S	24	23-133
Medtr7g093790.1	191	b/d	S	25	130
Medtr4g049550.1	179	f	S	28	31-103
Medtr5g096120.1	189	b/d	S	22	*54*-64-129-172-183
Medtr3g105630.1	189	b/d	S	23	91-112
Medtr3g105640.1	188	b/d	S	23	65-90-111-182
Medtr7g070390.1	192	b/d	S	22	*62*-132
Medtr4g013770.1	192	b/d	S	23	2-59-94-131
Medtr7g021300.1	389	e	other	-	-
Medtr8g073770.1	190	-	S	26	52-90
Medtr8g073850.1	190	-	S	26	52-90

Table S1. *Cont.*

Gene Accession	Length	Subfamily	Location	SP (length)	*N*-Glycosylation
Medtr8g106405.1	170	a	other	-	39-68
Medtr8g099135.1	185	a	S	21	51-64-121
Medtr8g099115.1	189	a	S	21	68-143
Medtr8g106450.1	180	a	S	29	49-78
Medtr0433s0040.1	191	b/d	other	24	*7*-*35*-*77*
Medtr1g046500.1	250	e	S	26	51
Medtr1g054525.1	192	b/d	S	26	63-134-137
Medtr1g115510.1	353	e	S	32	8-*58*-97
Medtr1g115515.1	236	e	M	28	52
Medtr1g046800.1	250	e	S	26	*51*
Medtr4g013345.1	196	b/d	S	22	*65*-79-136
Medtr4g062520.1	303	e	S	32	*94*
Medtr4g062460.1	257	e	S	31	*68*-82-139
Medtr4g013310.1	197	b/d	S	23	*68*-82-139
Medtr4g013330.1	199	b/d	S	25	*66*-139
Medtr4g013350.1	197	b/d	S	23	*66*-80-137
Medtr4g073950.1	192	b/d	S	25	*60*-132-186
Medtr4g013355.1	180	b/d	S	22	24-42-72
Medtr4g013325.1	199	b/d	S	23	*68*-82-139
Medtr4g013335.1	196	b/d	S	22	*67*-136
Medtr4g062470.1	304	e	S	31	*96*
Medtr4g078885.1	186	e	S	24	179
Medtr4g074020.1	99	b/d	S	23	-
Medtr4g049570.1	178	f	S	26	30-47
Medtr1g046490.1	236	e	other	-	*7*-*35*-*77*
Medtr4g013320.1	199	b/d	S	22	*68*-82-139
Medtr4g013385.1	191	b/d	S	24	44-60-74-130

Table S2. Expression values ± standard deviations relative to the heat map in Figure 2 and retrieved from the *Medicago* eFP browser.

Tissue	Medtr4g 122130.1	Medtr1g 056370.1	Medtr7g 093850.1	Medtr7g 093820.1	Medtr4g 078885.1	Medtr7g 093830.1	Medtr7g 093790.1	Medtr4g 049550.1	Medtr7g 070390.1	Medtr8g 073770.1	Medtr1g 046500.1	Medtr1g 054525.1	Medtr4g 062520.1	Medtr4g 013320.1	Medtr4g 013385.1	Medtr4g 073950.1
Root	457.1 ± 52.16	841.34 ± 142.83	2503.74 ± 469.78	614,16 ± 192.12	280.76 ± 41.02	614.16 ± 192.12	1068.5 ± 63.19	8.39 ± 9.64	243.23 ± 43.96	157.59 ± 12.9	968.42 ± 51.19	791.6 ± 31.06	3237.18 ± 249.53	85.07 ± 1.53	12734.9 ± 633.43	3484.67 ± 195.25
Stem	6.37 ± 1.33	8.51 ± 8.08	2264.75 ± 543.39	94.12 ± 15.05	6439.02 ± 414.88	94.12 ± 15.05	645.49 ± 44.05	16.65 ± 0.86	1004.8 ± 95.31	420.88 ± 47.27	31.59 ± 11.04	3712.7 ± 196.55	18.24 ± 11.12	605.5 ± 36.19	78.1 ± 45.67	1131.03 ± 91.34
Leaf with Petiolules	33.1 ± 21.38	32.87 ± 2.22	227.26 ± 59.28	41.52 ± 29.85	1382.79 ± 87.38	41.52 ± 29.85	1537.27 ± 12.81	12.8 ± 7.22	109.73 ± 14.28	1065.89 ± 95.95	43.67 ± 10.87	7096.96 ± 574.28	19.19 ± 20.77	45.5 ± 22.39	241.64 ± 28.35	393.18 ± 60.55
Vegetative Bud	34.6 ± 9.11	7.92 ± 4.84	1733.22 ± 564.59	31.01 ± 19.39	8153.66 ± 409.89	31.01 ± 19.39	346.94 ± 35.58	6.37 ± 6.16	452.48 ± 76.4	358.24 ± 6.13	7.5 ± 1.48	3165.97 ± 176.84	24.77 ± 9.56	503.5 ± 45.36	32.38 ± 11.49	540.19 ± 14.34
Flower	78.2 ± 8.04	14.06 ± 15.31	263.08 ± 16.3	53.55 ± 11.79	575.5 ± 50.95	53.55 ± 11.79	465.62 ± 12.89	45.09 ± 12.49	967.92 ± 72.69	213.1 ± 32.99	22.23 ± 21.12	2567.76 ± 283.83	173.03 ± 22.02	113.97 ± 26.42	23.35 ± 10.54	467.92 ± 25.02
Pod	29.0 ± 8.5	23.48 ± 16.33	245.41 ± 48.69	35.58 ± 23.24	660.9 ± 16.2	35.58 ± 23.24	322.14 ± 76.69	1071.18 ± 137.22	5645.75 ± 1142.7	374.17 ± 49.66	13.46 ± 10.92	2270.72 ± 324.81	56.15 ± 12.72	465.53 ± 48.51	16.28 ± 10.23	936.18 ± 89.01
Nodule Mature (4w)	2141.12 ± 75.73	82.13 ± 10.25	3451.47 ± 1054.2	338.52 ± 180.98	680.17 ± 201.03	338.52 ± 180.98	502.83 ± 31.29	27.23 ± 16.34	51.76 ± 13.23	149.31 ± 27.88	238.94 ± 17.7	986.46 ± 184.97	1166.64 ± 246.66	50.43 ± 8.71	1425.33 ± 278.31	5843.43 ± 348.56

Table S3. Details of *M. truncatula* peroxidases showing the accession numbers, length, occurrence of XYLAT elements and best hits in *A. thaliana*. Accession numbers and length were retrieved from Phytozome v10.2. MtPrx116 (partial amino acid sequence retrieved from PeroxiBase, no prediction from Phytozome, no EST found) was omitted from the Table. The (+) and (−) signs indicate XYLAT elements on the (+)- and (−)-strand respectively.

Name	Gene Accession	Length (amino acids)	XYLAT	*A. thaliana* Best Hit (e value)
MtPrx01	Medtr5g074860.1	325	-	AtPrx52 (8e − 121)
MtPrx02	Medtr5g074740.1	323	-	AtPrx52 (4e − 114)
MtPrx03	Medtr3g467600.1	324	-	AtPrx52 (7e − 111)
MtPrx04	Medtr7g107520.1	320	-	AtPrx03 (4e − 120)
MtPrx05	Medtr2g088770.1	326	1873 (−)	AtPrx39 (1e − 160)
MtPrx06	Medtr2g008710.1	325	-	AtPrx25 (3e − 144)
MtPrx07	Medtr3g092990.1	327	-	AtPrx03 (5e − 97)
MtPrx08	Medtr5g022870.1	316	1061 (−)	AtPrx64 (3e − 122)
MtPrx09	Medtr4g125940.1	330	-	AtPrx59 (3e − 145)
MtPrx10	Medtr2g029800.1	353	1770 (+)	AtPrx22 (8e − 129)
MtPrx11	Medtr2g029820.1	355	-	AtPrx54 (5e − 132)
MtPrx12	Medtr2g029815.1	352	111 (+); 1733 (−)	AtPrx54 (2e − 130)
MtPrx13	Medtr1g101830.1	322		AtPrx66 (1e − 164)
MtPrx14	Medtr4g127670.1	323	1518 (−)	AtPrx16 (1e − 173)
MtPrx15	Medtr2g029850.1	352	-	AtPrx54 (1e − 125)
MtPrx16	Medtr2g029750.1	354	-	AtPrx22 (1e − 127)
MtPrx17	Medtr2g029740.1	355	-	AtPrx22 (2e − 130)
MtPrx18	Medtr4g133800.1	335	-	AtPrx07 (6e − 105)
MtPrx19	Medtr1g077000.1	326	-	AtPrx47 (4e − 169)
MtPrx20	Medtr2g029830.1	355	-	AtPrx54 (2e − 130)
MtPrx21	Medtr2g008160.1	323	-	AtPrx43 (2e − 147)
MtPrx22	Medtr5g083860.1	325	-	AtPrx52 (3e − 161)
MtPrx23	Medtr7g093370.1	373	-	AtPrx53 (4e − 166)
MtPrx24	Medtr4g029200.1	338	-	AtPrx52 (1e − 122)
MtPrx25	Medtr8g076820.1	332	-	AtPrx72 (0.0)
MtPrx26	Medtr2g029860.1	353	-	AtPrx54 (3e − 129)
MtPrx27	Medtr2g040000.1	332	-	AtPrx59 (2e − 132)
MtPrx28	Medtr2g084020.1	318	-	AtPrx52 (6e − 133)
MtPrx29	Medtr5g074970.1	326	-	AtPrx67 (1e − 140)
MtPrx30	Medtr2g029730.1	345	1436 (+)	AtPrx53 (1e − 127)
MtPrx32	Medtr4g029190.1	341	-	AtPrx52 (6e − 118)
MtPrx33	Medtr7g072480.1	316	-	AtPrx52 (5e − 95)
MtPrx34	Medtr3g072190.1	318	-	AtPrx52 (2e − 126)
MtPrx35	Medtr2g029910.1	353	-	AtPrx54 (5e − 129)
MtPrx36	Medtr7g072510.1	312	-	AtPrx52 (2e − 131)
MtPrx37	Medtr3g072210.1	238	-	AtPrx52 (3e − 91)
MtPrx38	Medtr4g095450.1	323	-	AtPrx38 (9e − 162)
MtPrx39	Medtr1g115900.1	330	1460 (+)	AtPrx52 (1e − 112)
MtPrx40	Medtr5g049280.1	338	-	AtPrx11 (1e − 169)

Table S3. *Cont.*

Name	Gene Accession	Length (amino acids)	XYLAT	*A. thaliana* Best Hit (e value)
MtPrx41	Medtr7g029030.1	320	-	AtPrx55 (2e − 137)
MtPrx42	Medtr5g016010.1	328	-	AtPrx35 (3e − 153)
MtPrx43	Medtr2g084010.1	318	-	AtPrx52 (1e − 129)
MtPrx44	Medtr2g084000.1	319	1691 (−)	AtPrx52 (2e − 126)
MtPrx45	Medtr1g022970.1	347	-	AtPrx9 (1e − 172)
MtPrx46	Medtr5g021060.1	331	-	AtPrx72 (0.0)
MtPrx47	Medtr4g132110.1	335	-	AtPrx42 (0.0)
MtPrx48	Medtr6g043240.1	352	1982 (+)	AtPrx12(1e − 117)
MtPrx49	Medtr4g029180.1	321	-	AtPrx52 (1e − 95)
MtPrx50	Medtr1g038680.1	332	-	AtPrx39 (2e − 89)
MtPrx52	Medtr5g017850.1	326	-	AtPrx10 (4e − 132)
MtPrx53	Medtr4g046713.1	326	1928 (+)	AtPrx03 (5e − 144)
MtPrx54	Medtr3g094650.1	322	1941 (+)	AtPrx52 (2e − 159)
MtPrx55	Medtr1g025980.1	318	318 (+)	AtPrx64 (3e − 169)
MtPrx56	Medtr1g066380.1	325	-	AtPrx21 (3e − 177)
MtPrx57	Medtr2g084080.1	317	-	AtPrx52 (3e − 137)
MtPrx58	Medtr5g017860.1	326	892 (−)	AtPrx10 (1e − 130)
MtPrx60	Medtr2g099175.1	336	-	AtPrx26 (4e − 134)
MtPrx61	Medtr7g026990.1	323	460 (+)	AtPrx52 (3e − 112)
MtPrx63	Medtr2g084090.1	318	-	AtPrx52 (2e − 132)
MtPrx64	Medtr0147s0010.1	393	-	AtPrx48 (5e − 111)
MtPrx65	Medtr1g086320.1	333	590 (+)	AtPrx66 (6e − 82)
MtPrx66	Medtr4g114210.1	320	-	AtPrx25 (1e − 172)
MtPrx67	Medtr4g083710.1	321	23 (−); 222 (−)	AtPrx05 (9e − 163)
MtPrx70	Medtr5g074760.1	323	-	AtPrx52 (3e − 114)
MtPrx71	Medtr2g028980.1	355	-	AtPrx53 (4e − 125)
MtPrx74	Medtr4g087965.1	346	-	AtPrx19 (3e − 156)
MtPrx75	Medtr6g043460.1	348	-	AtPrx12 (1e − 100)
MtPrx76	Medtr4g074990.1	320	-	AtPrx5 (6e − 156)
MtPrx78	Medtr6g027440.1	319	-	AtPrx57 (2e − 105)
MtPrx79	Medtr6g008075.1	334	-	AtPrx63 (3e − 152)
MtPrx81	Medtr4g029170.1	321	-	AtPrx52 (8e − 128)
MtPrx82	Medtr2g067440.1	389	-	AtPrx48 (1e − 96)
MtPrx83	Medtr5g033470.1	377	-	AtPrx28 (3e − 64)
MtPrx84	Medtr3g094630.1	322	346 (+)	AtPrx52 (5e − 173)
MtPrx85	Medtr4g122640.1	336	-	AtPrx40 (4e − 136)
MtPrx86	Medtr5g074710.1	323	-	AtPrx52 (2e − 114)
MtPrx87	Medtr1g086490.1	342	-	AtPrx28 (5e − 82)
MtPrx88	Medtr7g086870.1	312	1026 (−)	AtPrx44 (1e − 120)
MtPrx89	Medtr7g086820.1	315	-	AtPrx44 (4e − 129)
MtPrx90	Medtr5g014100.1	332	-	AtPrx20 (2e − 121)
MtPrx91	Medtr8g062780.1	329	711 (+); 757 (−)	AtPrx65 (7e − 134)
MtPrx92	Medtr8g075100.1	319	-	AtPrx64 (6e − 123)
MtPrx93	Medtr4g132490.1	327	-	AtPrx29 (6e − 108)

Table S3. *Cont.*

Name	Gene Accession	Length (amino acids)	XYLAT	*A. thaliana* Best Hit (e value)
MtPrx94	Medtr4g030890.1	415	-	AtPrx17 (0.0)
MtPrx95	Medtr4g031140.1	343	-	AtPrx17 (5e − 175)
MtPrx97	Medtr2g084110.1	317	-	AtPrx52 (2e − 133)
MtPrx98	Medtr3g094670.1	322	-	AtPrx52 (2e − 134)
MtPrx100	Medtr2g437770.1	343	-	AtPrx07 (6e − 131)
MtPrx107	Medtr3g105790.1	324	-	AtPrx46 (1e − 144)
MtPrx110	Medtr4g074980.1	294	1040 (+)	AtPrx5 (3e − 128)
MtPrx111	Medtr2g029560.1	344	-	AtPrx54 (3e − 131)
MtPrx114	Medtr3g105790.1	324	-	AtPrx46 (1e − 144)

Table S4. Expression values ± standard deviations relative to the heat map in Figure 4 and retrieved from the *Medicago* eFP browser.

Tissue	MtPrx01	MtPrx03	MtPrx04	MtPrx06	MtPrx07	MtPrx08	MtPrx10	MtPrx13	MtPrx14	MtPrx15
Root	1594.56 ± 194.77	36.09 ± 9.1	8838.53 ± 1522.58	2148.74 ± 101.46	2591.25 ± 94.57	633.48 ± 51.82	15,320.5 ± 1278.95	5164.14 ± 436.11	6269.31 ± 545.36	21,339.63 ± 1506.78
Stem	10.63 ± 13.43	23.55 ± 21.13	74.02 ± 11.68	417.75 ± 24.32	20.09 ± 19.02	17,797.36 ± 858.95	661.28 ± 36.66	6996.69 ± 1232.39	30.47 ± 5.48	6690.71 ± 794.09
Leaf with Petiolules	7.94 ± 5.82	24 ± 16.48	24.66 ± 26.79	1062.55 ± 105.58	48.35 ± 14.63	4504.04 ± 277.34	1467.57 ± 160.8	2271.08 ± 198.85	47.51 ± 15.45	4962.9 ± 307.51
Vegetative Bud	17.56 ± 11.62	19.42 ± 9.01	43.96 ± 4.46	95.68 ± 43.47	32.34 ± 10.15	5183.08 ± 184.67	1846.61 ± 97.33	2084.12 ± 232.6	58.57 ± 8.46	10,255.53 ± 894.87
Flower	4.65 ± 2.7	31.54 ± 16.89	17.18 ± 10.51	788.98 ± 26.77	41.36 ± 22.05	3097.78 ± 365.62	90.06 ± 23.35	2383.17 ± 120.43	27.52 ± 14.16	1528.73 ± 109.39
Pod	86.96 ± 35.93	4.25 ± 1.91	86.25 ± 27.64	33.29 ± 9.95	33.08 ± 21.42	22,513.1 ± 1346.44	301.69 ± 75.01	6703.42 ± 1258.03	52.16 ± 18.58	9044.48 ± 447.21
Nodule Mature (4w)	1556.12 ± 334.65	13,258.56 ± 956.71	205.58 ± 69.52	293.35 ± 219.11	1998.81 ± 93.56	98.29 ± 28.85	9445.96 ± 679.62	399.55 ± 104.79	4180.66 ± 738.72	18,453.83 ± 727.15
**Tissue**	**MtPrx16**	**MtPrx18**	**MtPrx19**	**MtPrx20**	**MtPrx22**	**MtPrx23**	**MtPrx25**	**MtPrx27**	**MtPrx29**	**MtPrx30**
Root	15,663.03 ± 787.73	436.61 ± 21.3	12,407.7 ± 972.52	118.97 ± 21.53	2488.86 ± 127.76	1149.55 ± 104.15	3204.13 ± 236.1	4413.84 ± 262.51	6049.6 ± 490.06	20.81 ± 12.4
Stem	102.04 ± 17.6	7.64 ± 3.52	1071.46 ± 26.6	2457.01 ± 259.21	59.91 ± 13.41	490.2 ± 53.18	842.07 ± 68.86	52.5 ± 34.38	2591.73 ± 133.62	13.97 ± 14.73
Leaf with Petiolules	56.4 ± 25.67	11.3 ± 9.89	659.27 ± 64.09	6532.35 ± 540.54	66.06 ± 20.95	39.46 ± 20.81	187.16 ± 19.38	32.94 ± 27.16	297.57 ± 45.04	25.97 ± 1.11
Vegetative Bud	108.52 ± 24.16	18.08 ± 10.26	416.96 ± 6.66	4743.75 ± 119.62	58.28 ± 8.49	83.18 ± 15.43	185.39 ± 37.25	24.9 ± 10.97	539.91 ± 20.19	4.01 ± 1.46
Flower	24.27 ± 14.78	97.49 ± 16.47	7002.84 ± 163.01	827.91 ± 16.2	51.03 ± 16.83	248.74 ± 29.13	240.59 ± 49.82	8.63 ± 8.48	1610.46 ± 281.5	20.31 ± 26.04
Pod	46.7 ± 4.19	23.72 ± 14.55	462.52 ± 10.3	2802 ± 192.84	97.26 ± 22.91	10,166.21 ± 3760.63	264.29 ± 11.39	159.68 ± 63.58	354.71 ± 98.77	116.08 ± 25.15
Nodule Mature (4w)	7693.85 ± 482	37.78 ± 19.76	1214.38 ± 383.15	1305.82 ± 40.34	1728.34 ± 158.91	215.51 ± 39.84	557.9 ± 91.8	1903.93 ± 505.47	1432.26 ± 129	16.4 ± 9.55

Table S4. *Cont.*

Tissue	MtPrx36	MtPrx37	MtPrx38	MtPrx39	MtPrx41	MtPrx42	MtPrx43	MtPrx44	MtPrx47	MtPrx52
Root	6503.73 ± 294.23	76 ± 19.23	19,933.83 ± 1144.04	4591.92 ± 273.31	4988.4 ± 499.32	2626.66 ± 191.6	391.07 ± 37.65	3838.35 ± 79.5	10,583.07 ± 691.13	9628.54 ± 1011.28
Stem	14.92 ± 9.13	1769.5 ± 102.46	7259.35 ± 446.77	16.9 ± 4.67	183.57 ± 3.81	11,770.56 ± 753.09	56.45 ± 13.28	30.33 ± 27.81	2739.68 ± 112.86	19.51 ± 8.71
Leaf with Petiolules	16.38 ± 10.39	4982.21 ± 216.17	684.68 ± 47.49	6.23 ± 5.8	3648.11 ± 229.22	2764.65 ± 182.8	31.08 ± 11.95	39.41 ± 9.09	76.47 ± 24.68	13.63 ± 13.5
Vegetative Bud	9.46 ± 10.58	1668.85 ± 188.02	4429.07 ± 290.77	1.42 ± 0.39	279.3 ± 3.39	8579.15 ± 230.39	145.11 ± 24.69	40.08 ± 10.47	226.57 ± 35.97	19.84 ± 14.14
Flower	4.8 ± 2.6	582.12 ± 97.95	2772.81 ± 382.81	9.27 ± 10.24	58.87 ± 11.58	4376.59 ± 152.68	1053.52 ± 52.82	30.63 ± 26.39	737.37 ± 87.64	12.64 ± 7.58
Pod	4.16 ± 2.87	3752.27 ± 223.42	12,410.9 ± 1444.4	4.48 ± 3.68	68.64 ± 2.47	16,312.2 ± 1202.62	63.18 ± 11.55	34.37 ± 14.58	21,587.23 ± 1484.06	29.33 ± 11.78
Nodule Mature (4w)	2980.48 ± 311.15	351.93 ± 85.25	5289.36 ± 1198.59	152.58 ± 12.74	4559.98 ± 130.46	8266.33 ± 475.92	91.99 ± 36.57	2543.42 ± 517.57	7585.6 ± 503.42	362.31 ± 109.9

Tissue	MtPrx54	MtPrx55	MtPrx56	MtPrx57	MtPrx61	MtPrx63	MtPrx64	MtPrx66	MtPrx67	MtPrx71
Root	37.39 ± 14.36	837.09 ± 83.35	777.52 ± 141.69	85.56 ± 24.38	2862.53 ± 350.41	8.25 ± 6.38	14.46 ± 4.48	3538.31 ± 165.35	29.45 ± 15.3	214.84 ± 64.44
Stem	9057.28 ± 582.76	19.44 ± 11.44	148.12 ± 26.56	31.43 ± 8.56	13.83 ± 8.04	1.87 ± 0.7	16.09 ± 19.33	72.94 ± 4.57	38.43 ± 25.61	38.52 ± 2.6
Leaf with Petiolules	784.98 ± 61.34	11.44 ± 11.36	143.59 ± 16.52	35.43 ± 19.84	6.24 ± 3.12	19.63 ± 10.12	5.09 ± 2.41	20.35 ± 14.83	36.86 ± 22.5	26.53 ± 14.14
Vegetative Bud	2604.66 ± 83.88	8.71 ± 4.83	50.75 ± 3.21	28.4 ± 18.19	29.22 ± 20.16	16.2 ± 5.06	7.98 ± 4.59	16.19 ± 16.19	41.69 ± 24	37.8 ± 34.56
Flower	5620.42 ± 761.83	9.37 ± 9.97	171.02 ± 15.21	39.88 ± 15.82	15.35 ± 9.06	28.7 ± 21.94	11.19 ± 9.27	12.87 ± 11.36	9.44 ± 2.03	17.23 ± 5.32
Pod	1106.2 ± 272.13	23.45 ± 6.87	336.51 ± 84.03	33.69 ± 22.49	59.94 ± 21.76	920.87 ± 226.65	9.34 ± 7.18	11.08 ± 10.93	23.01 ± 16.18	11.01 ± 5.51
Nodule Mature (4w)	23.03 ± 23.74	56.52 ± 15.67	147.02 ± 50.74	45.95 ± 6.24	73.46 ± 22.25	26.86 ± 16.06	8.58 ± 3.89	881.46 ± 92.43	34.49 ± 16.46	36.06 ± 11.83

Table S4. *Cont.*

Tissue	MtPrx75	MtPrx78	MtPrx81	MtPrx83	MtPrx85	MtPrx86	MtPrx90	MtPrx91	MtPrx94	MtPrx110
Root	61.81 ± 31.8	23.88 ± 9.87	5885.43 ± 449.21	25.58 ± 11.67	10.56 ± 6.68	2198.78 ± 317.12	439.32 ± 27.16	31.87 ± 13.04	1017.54 ± 24.87	6.41 ± 6.72
Stem	7.06 ± 4.05	7.79 ± 3.56	8.85 ± 4.69	27.13 ± 13	56.23 ± 7.1	18.38 ± 12.44	12.19 ± 6.22	20.13 ± 21.75	631.45 ± 50.6	1.94 ± 0.27
Leaf with Petiolules	6.57 ± 3.3	20.35 ± 14.95	10.33 ± 10.95	18.93 ± 6.52	289.45 ± 53.47	12.31 ± 11.63	19.91 ± 8.48	31.16 ± 34.99	437.06 ± 97.53	23.14 ± 12.89
Vegetative Bud	9.41 ± 7.3	4.34 ± 4.18	8.83 ± 7.1	14.26 ± 13.19	26.14 ± 19.31	9.39 ± 5.27	14.02 ± 12.25	15.9 ± 11.71	661.99 ± 35.01	10.24 ± 12.02
Flower	4.59 ± 0.82	103.72 ± 26.82	11.29 ± 6.56	4980.06 ± 662.31	6.39 ± 1.56	5.98 ± 2.61	26.76 ± 17.83	276.67 ± 63.12	2079.65 ± 171.48	13.31 ± 15.1
Pod	13.96 ± 5.59	967.16 ± 223.73	2.46 ± 0.42	284.94 ± 71.42	6.94 ± 3.47	17.61 ± 13.42	23.21 ± 16.45	33.27 ± 2.55	1003.83 ± 34.45	13.88 ± 14.3
Nodule Mature (4w)	10.5 ± 3.58	11.96 ± 10.48	253.24 ± 31.77	31.64 ± 12.97	30.69 ± 24.45	127.2 ± 74.25	310.74 ± 65.14	8.91 ± 9.16	2324.17 ± 176.36	2.52 ± 0.58

Table S5. Normalized Relative Expression for *CesA* genes in alfalfa roots. Normalized Relative Expression values ± standard error of the mean and significance (Sign.). Data were normalized using PAB4/TFIIA. Means sharing a letter are not significantly different at α = 0.05 (Tukey post hoc test). ***** The asterisk indicates genes showing non-normal distribution of the variances, but individually ranked using the ANOVA one-way for clarity of presentation. These data have been analysed with the test of Kruskal-Wallis and have shown rejection the null hypothesis (*i.e.*, the mean ranks of the different groups are different).

Time Point	CesA1 *	Sign.	CesA3 *	Sign.	CesA6B *	Sign.	CesA6C *	Sign.	CesA6F *	Sign.	CesA4	Sign.	CesA7B *	Sign.	CesA7A *	Sign.	CesA8 *	Sign.
Ctrl 0 h	0.82 ± 0.12	a	1.32 ± 0.09	d	0.9 ± 0.07	abc	1.52 ± 0.23	cd	1.17 ± 0.06	bc	1.11 ± 0.14	bc	1.63 ± 0.13	c	1.69 ± 0.06	d	1.9 ± 0.16	d
Ctrl 24 h	0.85 ± 0.07	a	0.88 ± 0.01	abcd	0.9 ± 0.06	abc	1.86 ± 0.45	d	1.27 ± 0.17	bc	0.97 ± 0.11	bc	1.08 ± 0.12	c	1.12 ± 0.1	cd	1.32 ± 0.16	cd
Ctrl 72 h	1.09 ± 0.11	ab	1.11 ± 0.07	bcd	1.3 ± 0.05	bc	1.77 ± 0.08	d	2.07 ± 0.26	c	2.29 ± 0.12	c	1.92 ± 0.05	c	1.96 ± 0.35	d	2.6 ± 0.41	d
Ctrl 96 h	0.92 ± 0.13	a	0.97 ± 0.12	abcd	1.21 ± 0.21	bc	1.45 ± 0.14	cd	1.67 ± 0.09	bc	1.93 ± 0.31	bc	1.68 ± 0.19	c	2.08 ± 0.26	d	2.22 ± 0.45	d
Cold-24 h	1.7 ± 0.14	b	1.54 ± 0.34	d	1.47 ± 0.34	c	0.99 ± 0.17	cd	0.88 ± 0.13	abc	1.55 ± 0.22	bc	1.36 ± 0.1	c	1.05 ± 0.23	cd	1.83 ± 0.16	d
Cold-72 h	1.32 ± 0.02	ab	1.42 ± 0.03	d	1.26 ± 0.09	bc	0.94 ± 0.09	cd	0.72 ± 0.04	abc	1.63 ± 0.19	bc	1.5 ± 0.19	c	1.89 ± 0.19	d	1.92 ± 0.3	d
Cold-96 h	1.23 ± 0.08	ab	1.41 ± 0.21	d	1.33 ± 0.13	bc	0.7 ± 0.07	bc	1.07 ± 0.09	abc	1.07 ± 0.16	bc	1.5 ± 0.31	c	1.26 ± 0.1	cd	2.28 ± 0.57	d
Heat-24 h	0.93 ± 0.04	a	0.69 ± 0.05	abc	0.71 ± 0.06	ab	0.8 ± 0.19	bc	1.07 ± 0.15	abc	0.84 ± 0.2	b	0.8 ± 0.25	bc	0.62 ± 0.29	bc	0.39 ± 0.14	bc
Heat-72 h	0.79 ± 0.01	a	0.62 ± 0.04	ab	0.55 ± 0.01	a	0.38 ± 0.03	ab	0.6 ± 0.1	ab	0.26 ± 0.02	a	0.27 ± 0.06	ab	0.32 ± 0.01	ab	0.06 ± 0.01	a
Heat-96 h	0.8 ± 0.02	a	0.54 ± 0.01	a	0.77 ± 0.11	abc	0.3 ± 0.05	a	0.56 ± 0.38	a	0.21 ± 0.05	a	0.26 ± 0.14	a	0.18 ± 0.01	a	0.13 ± 0.06	ab
Salt-24 h	0.9 ± 0.08	a	1.2 ± 0.21	cd	1.06 ± 0.14	bc	1.34 ± 0.3	cd	1.03 ± 0.2	abc	1.12 ± 0.15	bc	1.31 ± 0.12	c	1.5 ± 0.13	d	1.74 ± 0.24	d
Salt-72 h	0.98 ± 0.06	a	0.97 ± 0.09	abcd	1.07 ± 0.05	bc	1.54 ± 0.05	cd	1.35 ± 0.09	bc	1.32 ± 0.16	bc	1.13 ± 0.07	c	1.01 ± 0.23	cd	1.67 ± 0.39	d
Salt-96 h	1.06 ± 0.19	ab	1.07 ± 0.07	bcd	1.07 ± 0.09	bc	1.45 ± 0.04	cd	1.08 ± 0.2	abc	1.5 ± 0.31	bc	1.37 ± 0.36	c	1.46 ± 0.32	d	2.14 ± 0.55	d

Table S6. Normalized Relative Expression for CAD, PAL, genes encoding dirigent-like proteins and class III peroxidases in alfalfa roots. Normalized Relative Expression values ± standard error of the mean and significance (Sign.). Data were normalized using PAB4/TFIIA. Means sharing a letter are not significantly different at α = 0.05 (Tukey post hoc test). * The asterisk indicates genes showing non-normal distribution of the variances, but individually ranked using the ANOVA one-way for clarity of presentation. These data have been analysed with the test of Kruskal-Wallis and have shown rejection the null hypothesis (*i.e.*, the mean ranks of the different groups are different).

Time Point	CAD	Sign.	PAL	Sign.	Prx13	Sign.	MsPrx12 *	Sign.	MsPrx16 *	Sign.	Medtr7g093850.1	Sign.	Medtr1g054525.1	Sign.
Ctrl 0 h	1.09 ± 0.13	ab	1.17 ± 0.28	a	1.13 ± 0.07	ab	1.17 ± 0.17	abc	1.55 ± 0.06	cde	0.56 ± 0.08	a	1.34 ± 0.17	cd
Ctrl 24 h	0.83 ± 0.07	a	0.87 ± 0.13	a	0.84 ± 0.1	a	1.19 ± 0.15	abc	1.12 ± 0.06	cd	0.87 ± 0.17	ab	1.23 ± 0.21	abc
Ctrl 72 h	1.47 ± 0.14	b	1.17 ± 0.24	a	2.52 ± 0.45	c	1.99 ± 0.23	c	2.47 ± 0.43	e	0.38 ± 0.18	a	2.33 ± 0.6	d
Ctrl 96 h	1.24 ± 0.14	ab	1.28 ± 0.45	a	1.83 ± 0.19	bc	1.31 ± 0.19	abc	2.06 ± 0.4	de	0.56 ± 0.08	a	1.34 ± 0.17	cd
Cold-24 h	0.9 ± 0.04	ab	0.82 ± 0.09	a	0.89 ± 0.1	ab	0.62 ± 0.17	a	1.12 ± 0.07	cd	1.17 ± 0.51	ab	0.87 ± 0.07	abc
Cold-72 h	0.97 ± 0.12	ab	0.98 ± 0.11	a	0.91 ± 0.15	ab	0.6 ± 0.03	a	1.51 ± 0.04	cde	1.01 ± 0.17	ab	1.16 ± 0.22	abc
Cold-96 h	0.81 ± 0.1	a	1.5 ± 0.12	a	0.76 ± 0.06	a	0.6 ± 0.07	a	0.79 ± 0.08	c	0.87 ± 0.17	ab	1.23 ± 0.21	abc
Heat-24 h	1.16 ± 0.02	ab	1.04 ± 0.22	a	1.26 ± 0.32	abc	1.42 ± 0.03	bc	0.35 ± 0.06	ab	1.3 ± 0.28	ab	1.12 ± 0.12	abc
Heat-72 h	1.13 ± 0.08	ab	0.98 ± 0.11	a	0.68 ± 0.07	a	0.66 ± 0.13	ab	0.17 ± 0.01	a	3.04 ± 0.5	b	0.55 ± 0.08	ab
Heat-96 h	0.74 ± 0.02	a	0.97 ± 0.24	a	0.62 ± 0.11	a	1.1 ± 0.08	abc	0.39 ± 0.08	b	1.17 ± 0.51	ab	0.87 ± 0.07	abc
Salt-24 h	0.92 ± 0.13	ab	1.14 ± 0.09	a	1.02 ± 0.18	ab	1.17 ± 0.29	abc	1.61 ± 0.2	cde	1.19 ± 0.25	ab	1 ± 0.12	abc
Salt-72 h	0.96 ± 0.12	ab	1.04 ± 0.1	a	0.88 ± 0.12	ab	1.47 ± 0.12	c	1.65 ± 0.13	de	0.36 ± 0.03	a	1.16 ± 0.17	abc
Salt-96 h	1.07 ± 0.08	ab	0.69 ± 0.07	a	0.98 ± 0.1	ab	0.89 ± 0.12	abc	1.64 ± 0.33	cde	1.3 ± 0.28	ab	1.12 ± 0.12	abc

Table S7. Normalized Relative Expression for *CesA* genes in alfalfa leaves. Normalized Relative Expression values ± standard error of the mean and significance (Sign.). Data were normalized using eif4A/TFIIA. Means sharing a letter are not significantly different at α = 0.05 (Tukey post hoc test). * The asterisk indicates genes showing non-normal distribution of the variances, but individually ranked using the ANOVA one-way for clarity of presentation. These data have been analysed with the test of Kruskal-Wallis and have shown rejection the null hypothesis (*i.e.*, the mean ranks of the different groups are different).

Time Point	CesA1 *	Sign.	CesA3 *	Sign.	CesA6B *	Sign.	CesA6C	Sign.	CesA6F *	Sign.	CesA4	Sign.	CesA7B	Sign.	CesA7A	Sign.	CesA8 *	Sign.
Ctrl 0 h	0.82 ± 0.09	a	1.05 ± 0.03	abc	0.81 ± 0.05	ab	0.97 ± 0.04	b	0.82 ± 0.04	a	0.97 ± 0.07	abc	1.21 ± 0.14	cd	0.97 ± 0.06	abc	1.97 ± 0.23	c
Ctrl 24 h	0.94 ± 0.08	ab	0.94 ± 0.12	ab	0.78 ± 0.04	ab	1.19 ± 0.16	b	1.11 ± 0.06	a	0.93 ± 0.04	abc	1.34 ± 0.13	cd	1 ± 0.04	abc	1.43 ± 0.2	c
Ctrl 72 h	0.95 ± 0.06	abc	0.83 ± 0.07	a	0.98 ± 0.05	abcd	1.16 ± 0.22	b	1.24 ± 0.26	a	1.1 ± 0.07	bcd	1.09 ± 0.19	cd	1.18 ± 0.06	abc	1.31 ± 0.12	c
Ctrl 96 h	1.34 ± 0.04	cd	1.12 ± 0.06	abc	1.1 ± 0.01	bcde	1.02 ± 0.11	b	0.89 ± 0.11	a	1.63 ± 0.2	cd	1.4 ± 0.22	cd	1.36 ± 0.27	bc	1.15 ± 0.3	c
Cold-24 h	1.05 ± 0	abc	1.13 ± 0.01	abc	1.45 ± 0.13	de	0.49 ± 0.03	a	0.92 ± 0.1	a	0.98 ± 0.08	abc	1.16 ± 0.15	cd	0.99 ± 0.16	abc	1.38 ± 0.2	c
Cold-72 h	1.53 ± 0.06	d	1.57 ± 0.16	c	1.67 ± 0.06	e	0.98 ± 0.07	b	1.08 ± 0.34	a	1.27 ± 0.27	bcd	1.45 ± 0.28	cd	1.19 ± 0.16	abc	1.39 ± 0.12	c
Cold-96 h	0.94 ± 0.07	ab	1.31 ± 0.06	bc	1.12 ± 0.04	bcde	0.45 ± 0.02	a	0.64 ± 0.06	a	0.93 ± 0.09	abc	1.06 ± 0.09	cd	0.62 ± 0.09	a	1.06 ± 0.23	bc
Heat-24 h	0.85 ± 0.07	a	0.79 ± 0.1	a	0.65 ± 0.05	a	1.09 ± 0.19	b	1.01 ± 0.08	a	0.72 ± 0.08	ab	0.77 ± 0.1	bc	1.09 ± 0.13	abc	0.59 ± 0.15	abc
Heat-72 h	0.91 ± 0.09	a	0.8 ± 0.1	a	0.82 ± 0.07	ab	1.31 ± 0.14	b	1.36 ± 0.15	a	0.66 ± 0.09	ab	0.31 ± 0.05	ab	0.82 ± 0.14	abc	0.29 ± 0.12	ab
Heat-96 h	0.89 ± 0.02	a	0.79 ± 0	a	0.89 ± 0.05	abc	1.2 ± 0.12	b	1.26 ± 0.02	a	0.52 ± 0.05	a	0.27 ± 0.11	a	0.63 ± 0.05	ab	0.19 ± 0.07	a
Salt-24 h	1 ± 0.02	abc	0.94 ± 0.01	ab	0.85 ± 0.04	ab	1.48 ± 0.23	b	0.88 ± 0.13	a	1.04 ± 0.06	bcd	1.35 ± 0.17	cd	1.06 ± 0.16	abc	1.3 ± 0.13	c
Salt-72 h	0.77 ± 0.03	a	0.96 ± 0.08	ab	0.98 ± 0.03	abcd	1.25 ± 0.29	b	1.28 ± 0.33	a	1.21 ± 0.21	bcd	1.62 ± 0.5	cd	1.2 ± 0.16	abc	3.04 ± 1.72	c
Salt-96 h	1.28 ± 0.03	bcd	1.07 ± 0.14	abc	1.41 ± 0.35	cde	1.26 ± 0.01	b	1.1 ± 0.34	a	1.94 ± 0.39	d	2.47 ± 0.73	d	1.53 ± 0.42	c	2.46 ± 0.92	c

Table S8. Normalized Relative Expression for CAD, PAL, genes encoding dirigent-like proteins and class III peroxidases in alfalfa leaves. Normalized Relative Expression values ± standard error of the mean and significance (Sign.). Data were normalized using eif4A/TFIIA. Means sharing a letter are not significantly different at α = 0.05 (Tukey post hoc test). * The asterisk indicates genes showing non-normal distribution of the variances, but individually ranked using the ANOVA one-way for clarity of presentation. These data have been analysed with the test of Kruskal-Wallis and have shown rejection the null hypothesis (*i.e.*, the mean ranks of the different groups are different).

Time Point	CAD	Sign.	PAL *	Sign.	Prx13	Sign.	MsPrx12	Sign.	Medtr7g093850.1 *	Sign.	Medtr1g054525.1	Sign.	Medtr4g078885.1	Sign.
Ctrl 0 h	1.14 ± 0.12	a	0.83 ± 0.11	ab	1.41 ± 0.04	ab	2.02 ± 0.29	ef	1.1 ± 0.3	ab	1.16 ± 0.09	bcd	0.9 ± 0.14	bcd
Ctrl 24 h	0.98 ± 0.13	a	0.58 ± 0.05	a	1.14 ± 0.01	ab	2.09 ± 0.22	ef	2.78 ± 1.04	b	1.12 ± 0.08	bcd	0.8 ± 0.21	abc
Ctrl 72 h	0.93 ± 0.18	a	0.75 ± 0.03	ab	1.04 ± 0.1	ab	1.07 ± 0.05	bcde	0.7 ± 0.58	a	1.91 ± 0.27	d	1.95 ± 0.49	cde
Ctrl 96 h	1.1 ± 0.19	a	1.92 ± 0.41	b	1.67 ± 0.34	b	0.42 ± 0.03	a	1.29 ± 0.42	ab	1.16 ± 0.25	bcd	2.46 ± 0.53	cde
Cold-24 h	0.66 ± 0.12	a	1.6 ± 0.25	ab	1.05 ± 0.15	ab	0.85 ± 0.09	abcd	0.46 ± 0.04	ab	1.09 ± 0.17	abcd	0.19 ± 0.05	a
Cold-72 h	1.34 ± 0.17	a	1.18 ± 0.08	ab	0.74 ± 0.23	a	0.57 ± 0.09	ab	0.63 ± 0.13	ab	0.91 ± 0.1	abc	0.27 ± 0.14	ab
Cold-96 h	0.91 ± 0.12	a	0.9 ± 0.07	ab	0.73 ± 0.09	ab	0.65 ± 0.09	abc	0.86 ± 0.3	ab	1.2 ± 0.05	bcd	0.25 ± 0.05	ab
Heat-24 h	1.21 ± 0.18	a	0.89 ± 0.2	ab	0.97 ± 0.12	ab	1.18 ± 0.13	cdef	0.91 ± 0.27	ab	0.56 ± 0.06	a	2.1 ± 0.35	cde
Heat-72 h	1.15 ± 0.09	a	1.2 ± 0.07	ab	0.72 ± 0.2	a	0.85 ± 0.14	abcd	1.94 ± 0.17	ab	0.55 ± 0.09	a	4.31 ± 1.06	de
Heat-96 h	1.08 ± 0.19	a	2.36 ± 1.2	b	0.72 ± 0.04	ab	0.62 ± 0.06	abc	1.77 ± 0.21	ab	0.64 ± 0.01	ab	5.52 ± 2.53	e
Salt-24 h	1.02 ± 0.19	a	0.59 ± 0.08	a	1.22 ± 0.09	ab	2.25 ± 0.23	f	0.71 ± 0.21	ab	1.08 ± 0.2	abcd	0.87 ± 0.25	abc
Salt-72 h	0.83 ± 0.09	a	0.7 ± 0.01	ab	1.08 ± 0.14	ab	1.67 ± 0.24	def	1.54 ± 0.36	ab	1.04 ± 0.16	abcd	0.8 ± 0.18	abc
Salt-96 h	1.07 ± 0.15	a	1.39 ± 0.47	ab	1.28 ± 0.34	ab	0.82 ± 0.18	abc	2.65 ± 0.67	b	1.53 ± 0.16	bc	2.01 ± 0.76	cde

Table S9. Normalized Relative Expression for genes encoding dirigent-like proteins and class III peroxidases in alfalfa stems. Normalized Relative Expression values ± standard error of the mean and significance (Sign.). Data were normalized using eif4A/PAB4. Means sharing a letter are not significantly different at α = 0.05 (Tukey post hoc test). * The asterisk indicates genes showing non-normal distribution of the variances, but individually ranked using the ANOVA one-way for clarity of presentation. These data have been analysed with the test of Kruskal-Wallis and have shown rejection the null hypothesis (*i.e.*, the mean ranks of the different groups are different).

Time Point	Medtr7g093850.1 *	Sign.	Medtr1g054525.1	Sign.	Medtr4g078885.1	Sign.	Prx13	Sign.	MsPrx12 *	Sign.	MsPrx16	Sign.
Ctrl 0 h	1.97 ± 0.87	ab	1.62 ± 0.25	e	1.94 ± 0.63	b	1.53 ± 0.3	bc	1.7 ± 0.33	f	1.04 ± 0.2	abc
Ctrl 24 h	1.68 ± 0.43	ab	1.35 ± 0.17	de	1.12 ± 0.11	b	0.98 ± 0.03	abc	1.54 ± 0.12	ef	0.85 ± 0.08	abc
Ctrl 72 h	0.51 ± 0.4	a	1.49 ± 0.1	e	0.61 ± 0.13	ab	1.79 ± 0.01	c	0.95 ± 0.06	bcde	1 ± 0.13	abc
Ctrl 96 h	1.77 ± 0.59	ab	1.45 ± 0.17	de	2.2 ± 0.77	b	1.89 ± 0.39	c	1.01 ± 0.14	bcdef	1.38 ± 0.09	bc
Cold-24 h	0.62 ± 0.03	ab	0.68 ± 0.1	abc	0.19 ± 0.05	a	1.1 ± 0.16	abc	0.4 ± 0.01	a	0.46 ± 0.07	a
Cold-72 h	0.39 ± 0.04	ab	0.81 ± 0.01	abcd	0.58 ± 0.11	ab	0.63 ± 0.05	a	0.61 ± 0.07	ab	0.86 ± 0.15	abc
Cold-96 h	1.09 ± 0.1	ab	1.11 ± 0.11	bcde	0.61 ± 0.17	ab	0.68 ± 0.01	a	0.86 ± 0.06	bcd	1.11 ± 0.15	bc
Heat-24 h	1.05 ± 0.21	ab	0.59 ± 0.02	a	0.93 ± 0.18	b	0.72 ± 0.13	a	1.08 ± 0.12	cdef	1.73 ± 0.38	c
Heat-72 h	1.27 ± 0.11	ab	0.46 ± 0.04	a	1.36 ± 0.3	b	0.8 ± 0.11	ab	0.86 ± 0.03	bcd	1.02 ± 0.27	abc
Heat-96 h	3.02 ± 0.66	b	0.64 ± 0.09	ab	2.03 ± 0.56	b	0.6 ± 0.03	a	0.77 ± 0.04	bc	1.17 ± 0.1	bc
Salt-24 h	0.56 ± 0.26	ab	1.23 ± 0.13	cde	1.86 ± 0.36	b	1.04 ± 0.02	abc	1.59 ± 0.02	ef	0.69 ± 0.09	ab
Salt-72 h	1.36 ± 0.26	ab	1.22 ± 0.08	cde	1.13 ± 0.5	b	1.24 ± 0.21	abc	1.36 ± 0.07	def	1.42 ± 0.22	bc
Salt-96 h	2.52 ± 0.19	b	1.48 ± 0.26	de	2.29 ± 0.31	b	1.14 ± 0.25	abc	1.3 ± 0.16	cdef	1.26 ± 0.11	bc

Table S10. List of primers used for the RT-qPCR in this study. The names of the primers are in bold. Details concerning the primer sequences, amplicon length and Tm, PCR efficiencies and regression coefficients are included.

Name	Sequence (5′→3′)	Amplicon Length (bp)	Amplicon Tm (°C)	PCR Efficiency (%)	Regression Coefficient (R^2)
**qPCR Medtr7g093850.1 Fwd**	ATCACCATCGCCACAATCAC	118	77.7	98.3	0.997
**qPCR Medtr7g093850.1 Rev**	TGTGTGACTCCAGCATTTCC				
**qPCR Medtr1g054525.1 Fwd**	TTCCGAAGTTGTGGGAAGAG	98	80.7	101.9	0.998
**qPCR Medtr1g054525.1 Rev**	TCCCTCGTGTGAAAGCAAAG				
**qPCR Medtr4g078885.1 Fwd**	ACTTGTAATCGTTGGTGTAGCAG	108	76.1	110.7	0.985
**qPCR Medtr4g078885.1 Rev**	GCACATCCTCTTTTCGTGTG				
**qPCR Prx13 Fwd**	CAGCCCGTATTTTGAGGATG	92	80.2	97.3	0.998
**qPCR Prx13 Rev**	TGCTTGGTTAGTGGCAGTTG				
**qPCR Prx38 Fwd**	GCTTCAGTTTTGGTGGCATC	137	78.5	93.0	0.995
**qPCR Prx38 Rev**	TTTCTGCACTGTGGAACAGC				
**qPCR Prx42 Fwd**	TCTTGCTATGGCAACTCGTG	132	79.6	96.1	0.989
**qPCR Prx42 Rev**	TTGAATCCAGGCTCAGGAAG				
